# EGFR inhibitors identified as a potential treatment for chordoma in a focused compound screen

**DOI:** 10.1002/path.4729

**Published:** 2016-05-31

**Authors:** Susanne Scheipl, Michelle Barnard, Lucia Cottone, Mette Jorgensen, David H Drewry, William J Zuercher, Fabrice Turlais, Hongtao Ye, Ana P Leite, James A Smith, Andreas Leithner, Peter Möller, Silke Brüderlein, Naomi Guppy, Fernanda Amary, Roberto Tirabosco, Sandra J Strauss, Nischalan Pillay, Adrienne M Flanagan

**Affiliations:** ^1^ University College London Cancer InstituteLondonUK; ^2^ Department of Orthopaedics and Orthopaedic SurgeryMedical University of GrazAustria; ^3^ Cancer Research Technology Discovery LaboratoriesCambridgeUK; ^4^ CRUK–MedImmune Alliance LaboratoryCambridgeUK; ^5^ GlaxoSmithKlineResearch Triangle ParkNCUSA; ^6^ SGC–UNC, UNC Eshelman School of PharmacyUniversity of North Carolina at Chapel HillNCUSA; ^7^ Department of HistopathologyRoyal National Orthopaedic HospitalStanmoreUK; ^8^ Institute of PathologyUlm UniversityGermany; ^9^ University College London Advanced DiagnosticsLondonUK

**Keywords:** chordoma, drug screen, EGFR, ERBB family, AZD8931, resistance

## Abstract

Chordoma is a rare malignant bone tumour with a poor prognosis and limited therapeutic options. We undertook a focused compound screen (FCS) against 1097 compounds on three well‐characterized chordoma cell lines; 154 compounds were selected from the single concentration screen (1 µm), based on their growth‐inhibitory effect. Their half‐maximal effective concentration (EC_50_) values were determined in chordoma cells and normal fibroblasts. Twenty‐seven of these compounds displayed chordoma selective cell kill and 21/27 (78%) were found to be EGFR/ERBB family inhibitors. EGFR inhibitors in clinical development were then studied on an extended cell line panel of seven chordoma cell lines, four of which were sensitive to EGFR inhibition. Sapitinib (AstraZeneca) emerged as the lead compound, followed by gefitinib (AstraZeneca) and erlotinib (Roche/Genentech). The compounds were shown to induce apoptosis in the sensitive cell lines and suppressed phospho‐EGFR and its downstream pathways in a dose‐dependent manner. Analysis of substituent patterns suggested that EGFR‐inhibitors with small aniline substituents in the 4‐position of the quinazoline ring were more effective than inhibitors with large substituents in that position. Sapitinib showed significantly reduced tumour growth in two xenograft mouse models (U‐CH1 xenograft and a patient‐derived xenograft, SF8894). One of the resistant cell lines (U‐CH2) was shown to express high levels of phospho‐MET, a known bypass signalling pathway to EGFR. Neither amplifications (EGFR, ERBB2, MET) nor mutations in EGFR, ERBB2, ERBB4, PIK3CA, BRAF, NRAS, KRAS, PTEN, MET or other cancer gene hotspots were detected in the cell lines. Our findings are consistent with the reported (p‐)EGFR expression in the majority of clinical samples, and provide evidence for exploring the efficacy of EGFR inhibitors in the treatment of patients with chordoma and studying possible resistance mechanisms to these compounds in vitro and in vivo. © 2016 The Authors. *The Journal of Pathology* published by John Wiley & Sons Ltd on behalf of Pathological Society of Great Britain and Ireland.

## Introduction

Chordoma is a rare primary malignant bone tumour showing notochordal differentiation and develops for the most part in the bones of the base of the skull, the vertebral bodies and the sacro‐coccygeal region 1, 2, 3. There are occasional reports of extra‐axial and soft tissue lesions 1, 4. The median survival for patients with chordoma is 7 years 2, 3. Advances in radiation technology with either particles or photons have allowed delivery of higher doses of radiation 2, 3 and can be beneficial for local disease control. However, 30–40% of chordomas metastasise and there are no approved agents for the treatment of patients with inoperable and metastatic chordoma 3. Cytotoxic chemotherapy is not active in this tumour type 3, 5. Imatinib, an inhibitor of platelet‐derived growth factor receptor (PDGFR), has demonstrated limited activity in a phase II study and when used in a compassionate programme 6, 7. However, there are encouraging results, in the form of anecdotal reports, on the response of chordoma to epidermal growth factor receptor (EGFR) 8, 9, 10, 11, 12, 13 and vascular endothelial growth factor (VEGF) inhibitors 5, 12, 13, 14, although data from prospective randomized clinical trials are lacking 5, 14.

Chordoma is characterised by the expression of the transcription factor *T* (*brachyury*) 15 and there is a body of evidence supporting its critical role in this disease 16. Specifically, study of the *T* regulatory network revealed that epidermal growth factor (EGF), transforming growth factor‐α (TGFα) and fibroblast growth factor 1 (FGF1) ligands, amongst others, are direct products of *T*‐mediated transcription 17. These findings are supported by strong immuno‐expression of the phosphorylated proteins in chordoma 18, 19, 20, 21.

Genotype‐directed therapy represents a major strategy for planning new cancer treatments, and this has demonstrated success by improving outcome in close to 70% of patients with non‐small cell lung cancer harbouring *EFGR* mutations, albeit for 1–2 years before developing resistance 22. However, despite chordomas being immunoreactive for the activated form of EGFR (p‐EGFR), they do not harbour *EGFR* mutations and only infrequently other currently potentially tractable targets, such as *PIK3CA* mutations 18, 23, 24, 25. In view of the unmet need for effective treatment of patients with chordoma, we undertook a large‐scale compound screen on three chordoma cell lines and validated the key target in an extended panel of seven cell lines, with the aim of finding therapies and understanding the mechanism by which this disease develops. Such approaches are reported to be more successful than target‐based approaches in identifying drug candidates with clinically relevant mechanisms of action 26, 27, 28.

## Materials and methods

### Cells and cell lines used in the screen

Seven human chordoma cell lines, U‐CH1, U‐CH2, U‐CH7, U‐CH10, MUG‐Chor1, JHC7 and UM‐Chor1, were studied (see supplementary material) and quality controlled by short‐tandem‐repeat (STR) analysis (DNA Diagnostic Centre, London, UK) (see supplementary material, Table S1) and regular *Mycoplasma* testing 29, 30, 31, 32, 33. In the absence of the availability of notochordal tissue, a transient embryonic structure considered to represent the origin of chordoma 15, human dermal fibroblasts (ATCC^®^ PCS‐201‐012™) were used as a non‐neoplastic control cell population. NCI‐N87 (ATCC^®^ CRL‐5822™), a gastric cancer cell line which strongly expresses EGFR and ERBB2 in the absence of downstream mutations 34, 35, served as a positive control to EGFR inhibitors. Cells were cultured according to ATCC guidelines (see supplementary material). All chordoma lines included derive from sacral tumours other than UM‐Chor1 which derives from a clival neoplasm (http://www.chordomafoundation.org/) 29, 30, 31, 32, 33. Cell pellets from the chordoma cell lines were formalin‐fixed and paraffin‐embedded and 3 µm sections cut for immunohistochemistry and FISH 18. Ethical approval was obtained from the Cambridgeshire 2 Research Ethics Service (reference 09/H0308/165) and the UCL Biobank for Health and Disease Ethics Committee.

### Protein kinase inhibitors and compound libraries

In collaboration with Cancer Research Technology Ldt UK (CRT), 1097 compounds were selected for the compound screen (see supplementary material, Table S2). GlaxoSmithKline (GSK) provided 886 small molecule kinase inhibitors comprising 365 ('PKIS') and 521 ('PKIS2') compounds on which there are published data (see supplementary material, Table S2) 36, 37. Also screened were 160 Calbiochem kinase inhibitors (Merck KGaA, Darmstadt, Germany) provided by CRT, an Anticancer Library (*n =* 43) (Selleckchem, Houston, TX, USA), and eight compounds reported to be inhibitors of aldo‐keto reductase family 1 member B10 (AKR1B10; Selleckchem) 38. Six commercially available epidermal growth factor receptor/erythroblastic leukaemia viral oncogene homologue (EGFR/ERBB) family inhibitors, either FDA‐approved or currently in clinical trials 39, 40, 41, 42, were purchased [Selleckchem: erlotinib (OSI‐774), gefitinib (ZD1839), sapitinib (AZD8931), afatinib (BIBW 2992), poziotinib (NOV120101; HM781‐36B)] and lapatinib (Tykerb^®^; GSK).

### Focused compound screen (Figure 1)

Compounds were tested on three chordoma cell lines (U‐CH1, U‐CH2 and MUG‐Chor1) using a non‐randomised plate layout in a 96‐well plate format (80 compounds/plate) at a single concentration of 1 µm (n = 3 minimum). Cells were seeded in medium (90 µl/well) using a Multidrop Combi (MDC; Thermo Fisher Scientific, Loughborough, UK) and cultured for 24 h before the compounds were added. The compounds were diluted from 10 mm stocks using an ECHO 550 (Labcyte, CA, USA) to create 10× compound plates and added (10 µl/well) using a Biomek FX (Beckman Coulter, Brea, CA, USA). Cell survival was assessed following 96 h of compound treatment using the water‐soluble tetrazolium salt (WST1) assay (Roche Diagnostics, Burgess Hill, UK) according to the manufacturer's recommendations.

**Figure 1:**
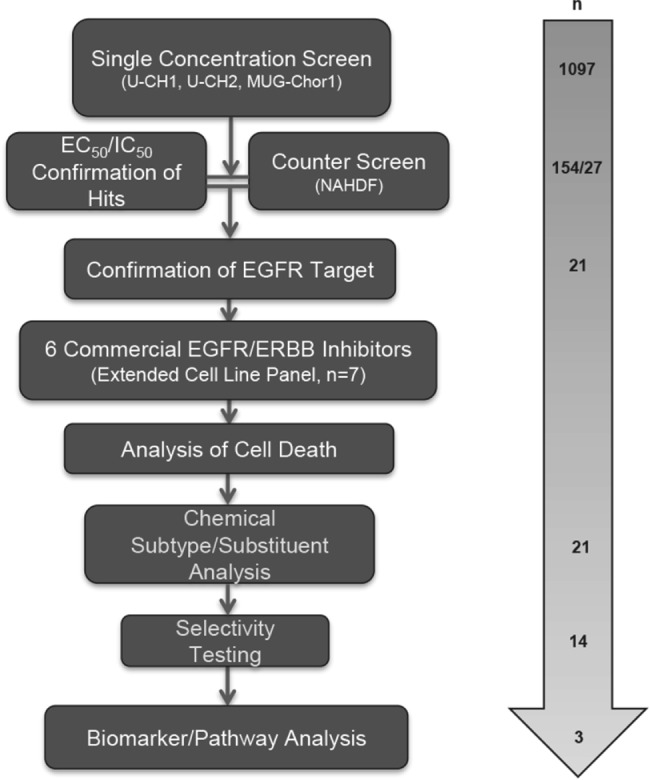
An overview of the screening cascade

### Hit selection

Hit selection thresholds were calculated independently for each cell line. Percentage inhibition for each compound at 1 µm was calculated from raw data relative to the controls on each plate. Thresholds were calculated by combining the results of the different libraries to give a mean percentage inhibition, or by analysing data obtained from the libraries independently (PKIS, PKIS2). From this, the standard deviations (SDs) were calculated for each cell line. Based on the spread of data, a threshold of 2 × SD (PKIS) and 1.5 × SD (other libraries) was applied for each line.

### Hit confirmation

The ‘hits’ of the single concentration screen and their potencies were generated using the half‐maximal effective concentration (EC_50_) in a 10‐point dose–response format, with the highest concentration at 30 µm. Maximum percentage inhibition (MI) was recorded at the highest concentration of each compound. Each compound was tested in three independent experiments with two replicates/experiment. Staurosporine (SRPN; Sigma‐Aldrich, St. Louis, MO, USA) was used as a positive control, with EC_50_ values monitored to ensure reproducibility between each run. An arbitrary threshold of EC_50_ < 5 µm in chordoma cell lines and > 10 µm in dermal fibroblasts was applied to select compounds which selectively killed chordoma cells. Selectivity was defined as the fold difference between a compound's EC_50_ in fibroblasts and chordoma cells.

### Hit validation

#### Analysis of cell death

The Caspase‐Glo^®^ 3/7 Assay (Promega, Southampton, UK) and the CellTiter‐Glo^®^ Luminescent Cell Viability Assay (Promega) were used on separate assay plates to monitor cell viability and to determine induction of apoptosis. Profiling was conducted from the highest concentration (20 µm) in a dose‐dependent manner with a 1:3 serial dilution (minimum two independent experiments/compound). Data analyses were performed using XLfit v. 5.0 (IDBS, Guildford, UK).

#### Biochemical selectivity analysis

Eleven GSK compounds in addition to sapitinib, erlotinib and gefitinib (Selleckchem) were sent for biochemical IC_50_ determination against EGFR, ERBB2 and ERBB4 (Reaction Biology Corp., Malvern, PA, USA) (see supplementary material, Table S3).

### Protein extraction and western blot (WB) analysis

Details of these methods are described in Supplementary materials and methods and primary and secondary antibodies are listed in Table S4 (see supplementary material for both).

### ELISA

Lysates were prepared and experiments performed using Human Total EGFR (cat. no. DYC1854) and Human Phospho‐EGFR (cat. no. DYC1095B) ELISA kits (R&D Systems, Abingdon, UK).

### Combination study of sapitinib with the MET inhibitor crizotinib

The MET inhibitor crizotinib (Xalkori^®^, Pfizer, NY, USA) was tested in combination with sapitinib using a non‐randomized plate layout in a 384‐well format. Details of this experiment are described in Supplementary materials and methods (see supplementary material).

### Immunohistochemistry

Immunohistochemistry was performed on the Leica Bond‐III detection platform, using the Bond Polymer Refine Detection system (Leica). Expression was evaluated as reported previously (see supplementary material) 18.

### FISH analysis of EGFR, ERBB2 and MET

FISH was performed using commercial probes for EGFR/CEP7, HER‐2/CEP17 (Abbott Molecular, Des Plaines, IL, USA) and MET/CEN7 (Zytovision, Bremerhaven, Bremen, Germany) and reported using the Colorado criteria (see supplementary material) 18.

### Analysis for mutations in cancer gene hotspots of chordoma cell lines

The hotspots in 22 cancer‐related genes were analysed for mutations using the Ion AmpliSeq™ Colon and Lung Cancer Research Panel v. 2 (Thermo Scientific) (see supplementary material).

### In vivo studies

Sapitinib (AstraZeneca, Cambridge, UK) was tested at South Texas Accelerated Research Therapeutics (START) on two chordoma mouse models: one model was a cell line‐derived xenograft (U‐CH1) 32, the other a patient‐derived xenograft (SF8894) 43 (see supplementary material).

## Results

### A focused compound screen showed that EGFR/ERBB family inhibitors targeted chordoma cells selectively

**Figure 2:**
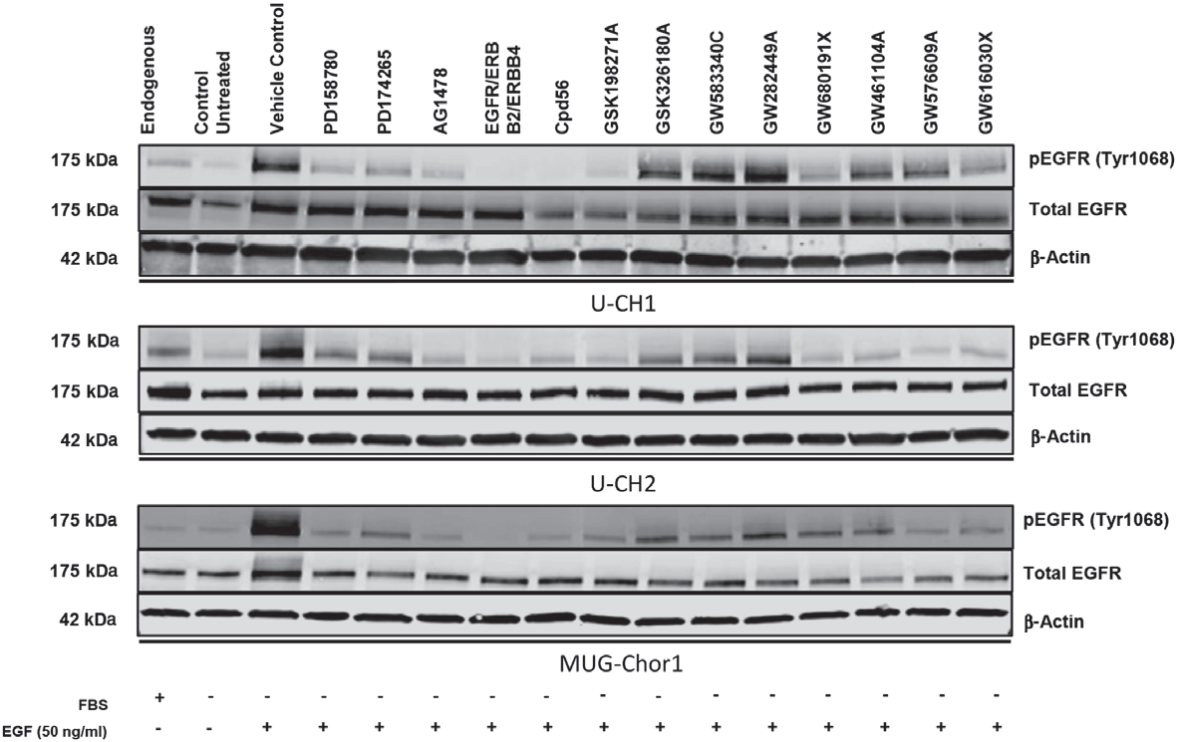
Hit compounds display varying effects on p‐EGFR and EGFR levels. Of 21 EGFR/ERBB hit compounds that selectively targeted chordoma cells, the impact of 13, comprising a selection of hit compounds across the libraries and chemical structures tested (listed in Tables 1, 2), was studied by western blot on three chordoma cell lines (U‐CH1, U‐CH2, MUG‐Chor1). Cells were serum‐starved overnight before being treated with EGFR inhibitors (250 nm) for 4 h and then being exposed to EGF (50 ng/ml) for 15 min.

Of the 1097 compounds screened at a single concentration in three human chordoma cell lines (U‐CH1, U‐CH2 and MUG‐Chor1; see supplementary material, Table S2), 154 met our hit selection criteria (see supplementary material, Table S2), which represented 14% (154/1097) of all compounds (see supplementary material, Table S5). Of these, 27 compounds selectively targeted chordoma cells but not human dermal fibroblasts (Table 1), and 21 of these 27 compounds (78%) represented EGFR/ERBB family inhibitors (Table 1, Figure 2), of which five also represented BRAF inhibitors. The results were reproducible using two different batches of compounds across all cell lines (data not shown). These 21 EGFR/ERBB inhibitors exerted the highest potency and maximum effects on U‐CH1, whereas negligible activity was observed on U‐CH2 (Table 1).

**Table 1:**
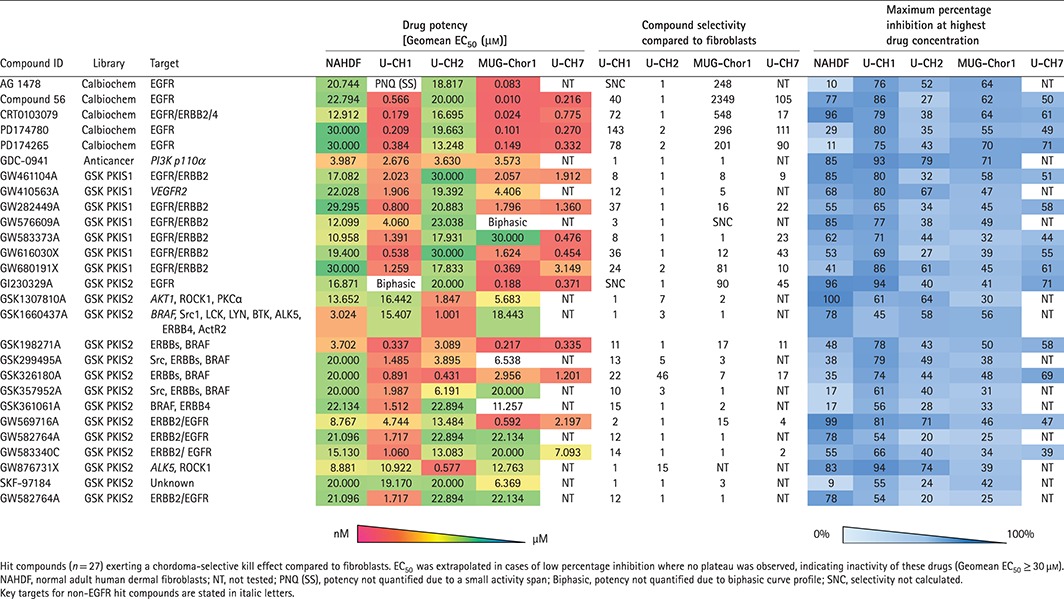
Chordoma‐selective hit compounds (n = 27)

Key targets for the remaining 6/27 non‐EGFR/ERBB compounds (Table 1) included activin receptor‐like kinase 5 (ALK5), phosphatidylinositol‐3‐kinase (PI3K), BRAF, as well as protein kinase‐Bα (AKT1) 44, 45, 46, 47. One compound, pazopanib (Votrient^®^; GSK), an angiogenesis inhibitor, has been FDA‐approved for renal cell carcinoma and soft‐tissue sarcomas 48, 49. Another compound has an unknown target. When these targets were examined for potential enrichment in signalling pathways 50, VEGFR1/2 signalling was identified as the pathway covering most of the non‐EGFR target genes (see supplementary material, Table S6).

### Chemical substituents correlated with EGFR/ERBB inhibitor activity in chordoma cell lines

Twenty‐one EGFR/ERBB inhibitors were selected by GSK for structural analysis on the basis of their phenotypic (viability) potencies (Table 2). A number of these compounds are currently under clinical development. These compounds were found to represent two chemotypes, pyrimidines and two subtypes of quinazolines. The quinazolines were characterised by either large substituents off the aniline group in the 4‐position of the quinazoline ring (hereafter referred to as 'quinazolines large'), or small substituents on the aniline ring in this position ('quinazolines small'). 'Quinazoline small' compounds, including erlotinib, sapitinib and gefitinib, exerted activity on U‐CH1, U‐CH7 and MUG‐Chor1 and showed a trend to greater potency than 'quinazoline large' compounds (Table 2). Neither subtype was active on U‐CH2. Biochemical assessment of the IC_50_ on a selection of these compounds (*n =* 14) on EGFR, ERBB2 and ERBB4 (ERBB3 was not tested) showed that both the size of the substituent group on the aniline ring and the structure of the tail portion of the molecule, which extends towards the solvent front of the kinase, had an impact on the potencies of the three targets (see supplementary material, Table S3).

**Table 2:**
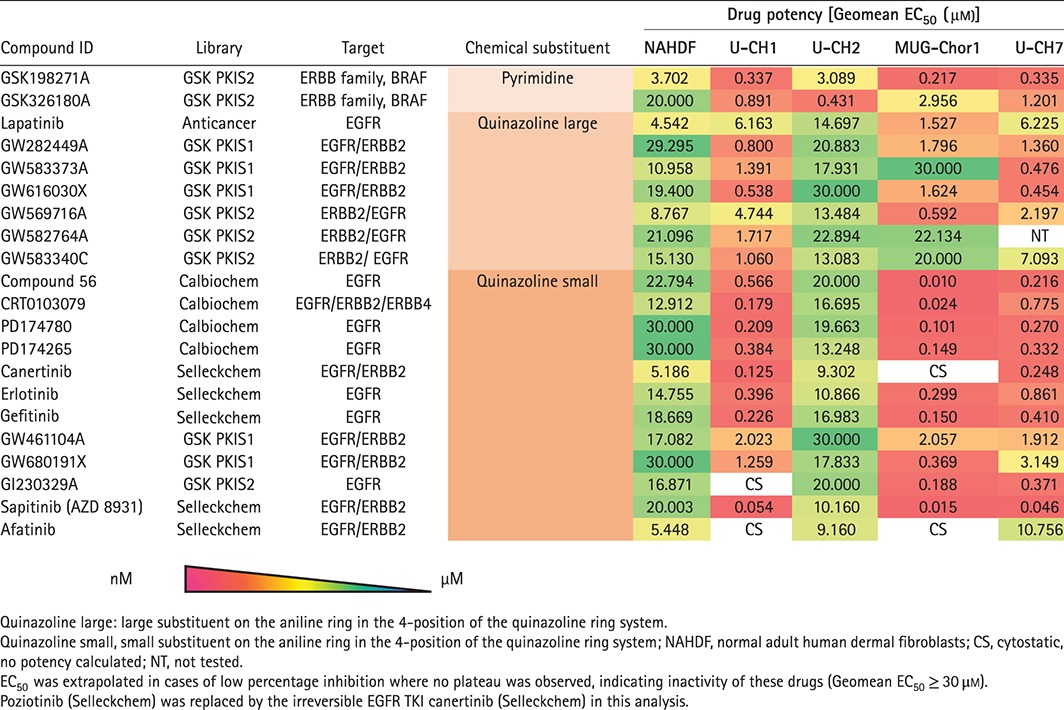
Chemical substituent trend analysis of selected EGFR/ERBB inhibitors (n = 21)

### Commercially available EGFR/ERBB family inhibitors exerted good potency and high selectivity in chordoma cell lines

We next tested a set of six commercially available EGFR/ERBB inhibitors consisting of four reversible (erlotinib, sapitinib, gefitinib and lapatinib) and two irreversible (afatinib and poziotinib) compounds in an extended panel of seven chordoma cell lines, including the three previously tested ones (Table 3). Controls included human dermal fibroblasts (ATCC^®^ PCS‐201‐012™) and the gastric cancer cell line NCI‐N87 (Table 3). Four of the chordoma cell lines, U‐CH1, U‐CH7, UM‐Chor1 and MUG‐Chor1, responded to EGFR inhibition with EC_50_ concentrations < 1 µm, whereas U‐CH2, U‐CH10 and JHC7 were largely resistant (Table 3). Three of four reversible agents, erlotinib, gefitinib and sapitinib, were highly potent, exerting an effect in the nanomolar range in the four ‘responsive’ cell lines. In contrast, lapatinib was potent on UM‐Chor1 (EC_50_ 320 nm and 98% MI), showed only moderate activity (EC_50_ ≥ 1 µm ≤ 3 µm) on MUG‐Chor1 and no activity on the other cell lines (EC_50_ ≥ 3 µm) (Table 3). Of the two irreversible EGFR/ERBB inhibitors, afatinib and poziotinib, the former displayed a significant kill effect on UM‐Chor1 (EC_50_ of 26 nm and 89% MI) and a cytostatic profile on U‐CH1 and MUG‐Chor1. Poziotinib displayed a cytostatic profile on U‐CH1, MUG‐Chor1 and UM‐Chor1: U‐CH7 was resistant to both (Table 3). Sapitinib, a 'quinazoline small' compound, showed the most exciting results, with EC_50_ concentrations in the nanomolar range in the four ‘responsive’ lines comparable to those observed in non‐small cell lung cancer (NSCLC) and head and neck cancer cell lines defined as being sensitive to EGFR 51. In these other cancer models, responses to the drug were also observed in related mouse xenograft models. The other active compounds were two FDA‐approved 'quinazoline small' compounds, gefitinib and erlotinib (Table 3).

**Table 3:**
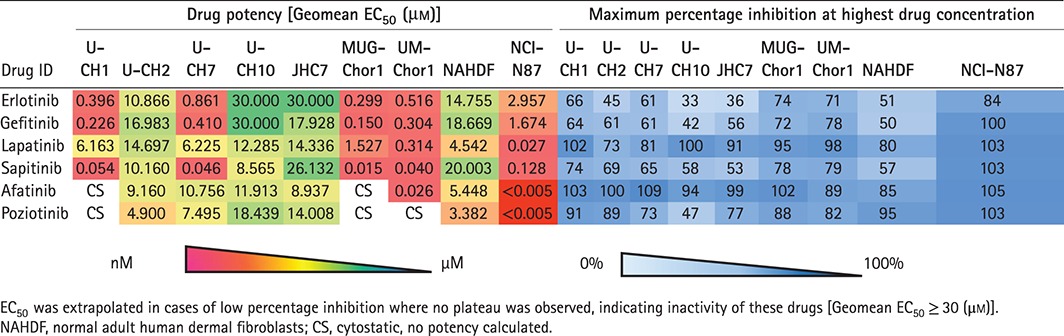
Phenotypic activity of commercially available EGFR/ERBB inhibitors (n = 6)

### EGFR/ERBB family inhibitors suppressed p‐EGFR and downstream effectors in chordoma cell lines and induced apoptosis in sensitive cell lines

**Figure 3:**
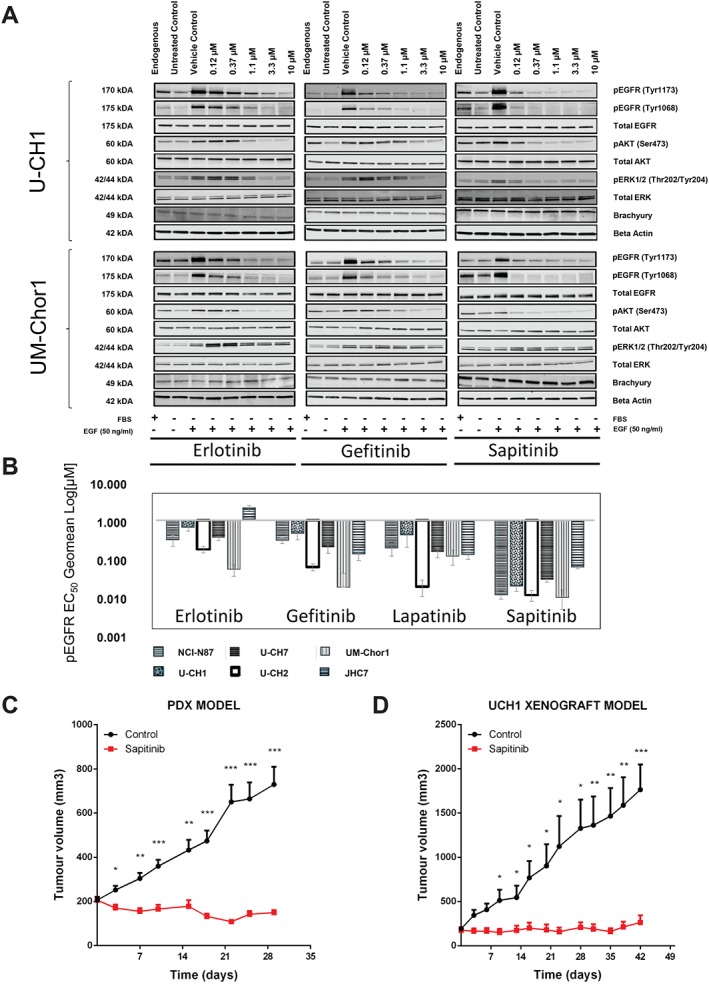
Western blot (A) and ELISA (B) analysis confirm suppression of the biomarker p‐EGFR upon treatment with EGFR TKIs in U‐CH1 and UM‐Chor1. Cells were serum‐starved overnight before they were treated with a range of concentrations of the EGFR inhibitors for 4 h and then EGF‐spiked (50 ng/ml) for 15 min. Endogenous controls (non‐serum‐starved, non‐EGF‐spiked), untreated controls (serum‐starved, non‐EGF‐spiked) and vehicle controls (serum‐starved, treated with 2.5% DMSO, EGF‐spiked) were included. Phospho‐EGFR was measured by western blot and ELISA. Western blot results for U‐CH2, U‐CH7, JCH7 and MUG‐Chor1 are displayed in supplementary material, Figure S2. (C, D) Sapitinib induces a significant growth reduction in the patient‐derived xenograft SF8894 (C) and in the U‐CH1 xenograft (D); *p ≤ 0.05, **p ≤ 0.01, ***p ≤ 0.001

ELISA and western blot data showed that our three most promising compounds (sapitinib > gefitinib > erlotinib) induced a dose‐dependent suppression of the biomarker p‐EGFR at two different phosphorylation sites (Tyr1068 and Tyr1173) in all cell lines, confirming that the drugs hit their key target. Similar results were shown in EGF spiked/serum‐starved (Figure 3; see also supplementary material, Figure S2) and non‐spiked/non‐starved experiments (see supplementary material, Figures S3, S4). Key effectors of EGFR signalling, including p‐AKT (PI3K–AKT–mTOR) and p‐ERK1/2 (Ras–Raf–MAPK–ERK1/2) were also dose‐dependently suppressed in response to these treatments. We did not observe significant effects on p‐STAT3 (see supplementary material, Figures S3, S4). Inhibition of p‐EGFR and downstream targets occurred at lower doses with sapitinib than with the other EGFR inhibitors tested. The endogenous/baseline status for all of the markers investigated is shown in Figure S5 (see supplementary material).

**Figure 4:**
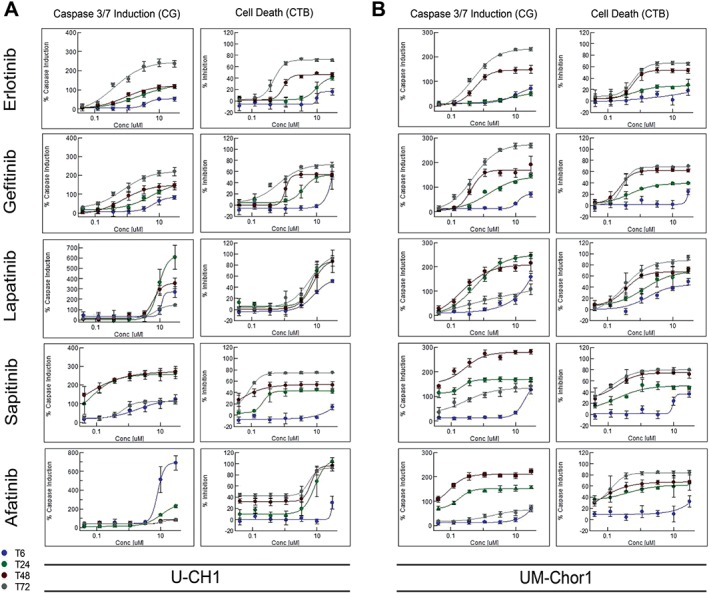
Apoptotic induction in U‐CH1 (A) and UM‐Chor1 (B). The Caspase‐Glo^®^ 3/7 Assay and the CellTiter‐Glo^®^ Luminescent Cell Viability Assay were used on separate assay plates to monitor cell viability and to determine induction of apoptosis upon treatment with erlotinib, gefitinib, sapitinib, afatinib and lapatinib. Read‐outs were performed at four time points (6, 24, 48 and 72 h). Two independent experiments were conducted for each compound (n = 3 for sapitinib and erlotinib). The results for U‐CH7 and MUG‐Chor1 are shown in supplementary material, Figure S6

The EGFR/ERBB inhibitors induced apoptosis in a dose‐dependent manner. This resulted in a decrease in cell viability from 24 h onwards (Figure 4; see also supplementary material, Figure S6). Where a cytostatic profile was generated in response to EGFR inhibitors, such as with lapatinib in the U‐CH1 cell line, minimal caspase 3/7 activity (∼30%) was observed in support of these phenotypic data (Figure 4; see also supplementary material, Figure S6).

### Sapitinib significantly reduced tumour growth in chordoma mouse models

Our most promising compound, sapitinib, significantly reduced tumour growth in two chordoma xenograft models (Figure 3C, D).

### Study of resistance mechanisms in U‐CH2, JHC7 and U‐CH10

Next we sought reasons why our best compounds were not effective in the unresponsive chordoma cell lines. We addressed the issue of EGFR tyrosine kinase inhibitor resistance by investigating known mechanisms of resistance from work in other cancer types. Next‐generation sequencing failed to detect mutations covering the hotspots in 22 tumour‐related genes, which included *EGFR*, *ERBB2* and *ERBB4* and their downstream effectors (*KRAS*, *BRAF*, *PIK3CA*, *AKT1*, *PTEN*, *NRAS* and *MAPK*). Thus, we were not able to detect obvious genetic explanations for sensitivity and/or resistance in our cell line panel.

**Figure 5:**
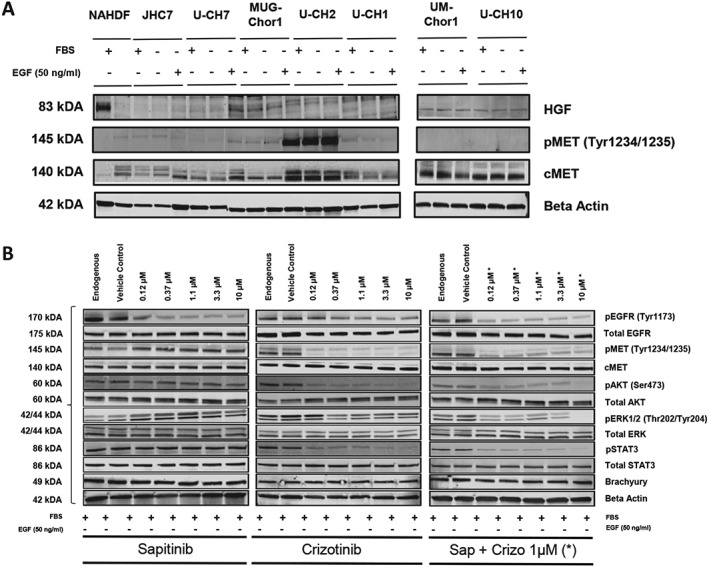
MET expression in the chordoma cell line panel. (A) Western blot analyses for MET‐expression in the cell line panel were conducted on endogenous (non‐serum‐starved, non‐EGF‐spiked), serum‐starved (serum‐starved, non‐EGF‐spiked) and EGF‐spiked (serum‐starved, EGF‐spiked) samples of each chordoma cell line. Normal adult human dermal fibroblasts (NAHDF) served as a control. Both western blots and immunohistochemistry (data shown in supplementary material, Figure 6B) revealed strong p‐MET expression in U‐CH2, a cell line resistant to EGFR TKIs, but not in the other chordoma cell lines. (B) Western blots of U‐CH2 treated with reagents as indicated for 4 h

**Figure 6:**
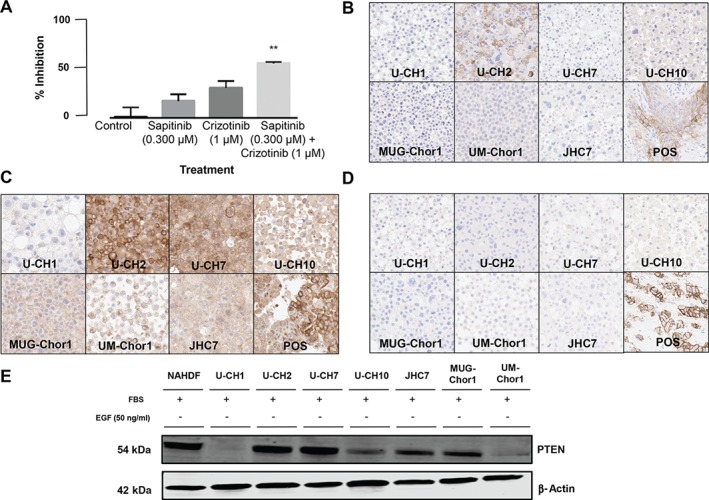
Combination treatment of the EGFR TKI sapitinib and the MET inhibitor crizotinib revealed significant synergy. (A) U‐CH2 cells were plated with a Multidrop Combi in a 384‐well format: after 24 h, cells were treated with crizotinib for 72 h, followed by sapitinib for another 24 h (n = 4 for combination; n = 3 for compounds alone); a combination index (CI) was calculated and evaluated as synergistic (CI < 0.9), additive (CI = 0.9–1.1) or antagonistic (CI > 1.1) 86; we observed a significant synergistic effect when sapitinib (300 nm) was combined with the MET inhibitor crizotinib (1 µm) in U‐CH2 (MI 58%; CI = 0.121; combination versus control, **p = 0.0047). (B) Immunohistochemistry was conducted on formalin‐fixed, paraffin‐embedded pellets of all seven chordoma cell lines, normal adult human dermal fibroblasts (NAHDF) and positive controls (POS): all images were taken at × 20 magnification; results for p‐MET showed strong expression in U‐CH2, concordant with the results obtained in western blot analysis (Figure 5), but not in the other cell lines. (C) PTEN expression was absent in U‐CH1, weak in UM‐Chor1 and positive to varying degrees in the other cell lines. (D) E‐Cadherin was expressed weakly and only focally in U‐CH7 and MUG‐Chor1 and was negative in the remaining five cell lines. (E) Western blots on the chordoma cell line panel (n = 7) and NAHDF confirmed an absence of PTEN in U‐CH1, weak expression in UM‐Chor1 and varying positivity in the other cell lines, as observed in IHC (C)

Activation of ERBB2 and MET can result in bypass resistance pathways. Together with *EGFR*, no amplifications in *ERBB2* and *MET* were identified by FISH in the cell lines (see supplementary material, Table S7). Moreover, no mutations were identified in *MET*. Furthermore, *MET* amplification, assessed by FISH, was only seen in two (non‐clival) of 114 clinical chordomas, 66 of which were located along the spinal axis and 48 in the clivus. However, western blots and immunohistochemistry for p‐MET revealed strong expression in U‐CH2 (Figures 5A, 6B). To test whether this contributed to the resistance observed, we treated U‐CH2 with sapitinib in combination with the MET‐inhibitor crizotinib and observed a significant synergistic effect (Figures 5B, 6A).

The loss and decreased protein expression of *PTEN*, a tumour suppressor gene, has also been linked to EGFR tyrosine kinase inhibitor (TKI) resistance 52, 53. All cell lines apart from U‐CH1 showed variable immunoreactivity with a PTEN antibody, strong in U‐CH2 and U‐CH7, moderate in MUG‐Chor1, JHC7 and U‐CH10 and weak in UM‐Chor1. These data were confirmed by western blots for PTEN (Figure 6C and 6E). Resistance has also been associated with loss of E‐Cadherin expression: it has been proposed that this results in epithelial–mesenchymal transition (EMT) and a related increase in motility 52, 54. Immunohistochemistry for E‐Cadherin showed weak and only focal expression in U‐CH7 and MUG‐Chor1 and was negative in the remaining five cell lines (Figure 6D). The Hippo downstream effector Yes‐associated protein (YAP) 55, 56 has also been reported to confer resistance to EGFR inhibitors 57, 58, 59. Even though YAP was highly expressed in the resistant cell line U‐CH10, strong expression levels were also observed in MUG‐Chor1, a cell line responsive to EGFR TKIs (see supplementary material, Figure S5). Hence, PTEN, E‐Cadherin, and YAP protein expression in the cell lines appear not to show an obvious correlation with the presence and/or absence of resistance to EGFR inhibitors.

## Discussion

In the light of the absence of recurrent genetic alterations in chordoma, we chose a phenotypic screening approach to identify the mechanism(s) by which this disease is driven and/or targets that could potentially be translated into clinical practice 28. We screened three chordoma cell lines (U‐CH1, U‐CH2 and MUG‐Chor1) against 1097 compounds, 1046 of which were small molecule kinase inhibitors. Twenty‐seven compounds remained of interest following exclusion of compounds that failed to mediate a chordoma‐selective cell kill effect. The majority (21/27) of these compounds targeted the EGFR/ERBB family. Gene enrichment analysis for non‐EGFR hit compounds revealed that VEGFR1/2 signalling covers most of their target genes, which is in line with isolated case reports showing activity of VEGF inhibitors in patients with chordoma 12, 13. However, since most of the non‐EGFR hits were multikinase inhibitors and their targets ill‐defined, and/or the compounds not phenotypically as potent as the EGFR/ERBB inhibitors, we focused on EGFR inhibitors in this study. As the majority of these 21 EGFR inhibitors remain under development, we tested six other EGFR inhibitors [erlotinib (Roche/Genentech); gefitinib (AstraZeneca); sapitinib (AstraZeneca); lapatinib (GSK); afatinib (Boehringer Ingelheim, Germany); and poziotinib (Spectrum Pharmaceuticals, Irvine, CA, USA)] that were either FDA‐approved or have been in clinical trials 39, 40, 42, 60. Of the seven chordoma cell lines tested, we demonstrated that four (U‐CH1, U‐CH7, MUG‐Chor1 and UM‐Chor1) were sensitive and three (U‐CH2, U‐CH10 and JHC7) were resistant to EGFR inhibition. The limitations of all *in vitro* screens apply to our study, but we imposed a high level of quality control measurements to ensure the generation of robust data 26, 27, 28.

EGFR was the first tyrosine kinase receptor to be linked to tumourigenesis 52, 61, 62, 63 and therapeutic inhibition of this pathway has yielded varying success in the treatment of malignant disease 39, 52, 62. A common cause for EGFR activation is the presence of mutations and gene amplification, as seen in non‐small cell lung cancer and glioblastoma 22, 39, 64, 65. However, this is not the case for the cell lines in our study, which is consistent with published *EGFR* genetic profiling reports 19, 23 and the unpublished data from ∼30 whole genomes/whole exomes from our laboratory. The absence of *EGFR* mutations in chordomas is shared with other cancers, such as head and neck squamous cell carcinoma and colorectal and pancreatic cancers, which are known to respond to anti‐EGFR therapy to varying degrees 52, 66, 67, 68, 69, 70, 71, 72. Similar to these tumours, chordoma cell lines express the activated form of the receptor and show suppression of the downstream EGFR signalling pathways following treatment with EGFR inhibitors. The clinical relevance of our *in vitro* studies is supported by the documented expression of these markers in patients' samples 18, 19, 20, 21, 73, 74, 75. Specifically, up to 52% of 170 chordoma samples have been reported to express p‐EGFR 18, 19, 20, 21, 76, although as phosphorylated protein is unstable, this is likely to be an underestimate 19, 21, 73, 74. The significance of the finding that erlotinib had a significant kill effect on four of seven cell lines tested is supported by the response to erlotinib seen in a well‐characterised, patient‐derived chordoma xenograft mouse model 77, which is consistent with the significant growth reduction we observed for sapitinib in two xenograft mouse models. There are also a number of well‐documented reports of patients with chordoma showing partial regression and/or clinical improvement following EGFR TKI treatment 8, 9, 10, 11, 12, 13, 78. In contrast, a small non‐randomised phase II clinical trial with the FDA‐approved dual EGFR/ERBB2 inhibitor lapatinib (Tykerb^®^/Tyverb^®^), involving 18 patients with advanced chordoma, showed only a modest clinical success with partial intratumoural response in six of 18 patients, according to Choi 76. This finding is consistent with the failure of lapatinib to exert a kill effect at therapeutic concentrations in all but one of our chordoma cell lines. In an attempt to address differences in the phenotypic kill effect observed in response to different EGFR TKIs in our large compound panel, we undertook analysis of their chemical structures and substituents. The overall finding was that compounds such as sapitinib, with small substituents appended to the aniline ring in the 4‐position of the quinazoline ring system, were more effective than compounds such as lapatinib, with large substituents, albeit these findings derive from a small sample size. However, the complexities of this potency, selectivity and phenotypic response relationship demand further study so that activity can be optimized for chordoma patients.

The expression of p‐EGFR in chordoma may be explained on the basis that epidermal growth factor (EGF) ligands are direct targets of *T* 17, the expression of which is considered to be critical in the growth of this tumour. This could also explain why *T* expression is not suppressed on western blot in response to EGFR inhibitors. It is therefore interesting that early‐phase clinical trials involving vaccines against *T* in patients with lung cancer and chordoma are showing some evidence of clinical activity 79, 80, 81, and it would be of interest to know whether p‐EGFR is suppressed in the clinical samples from these patients. The combination of one these vaccines and EGFR inhibition may be more effective than a monotherapy.

In an attempt to understand why three of seven cell lines were resistant to EGFR inhibitors, we studied the common mechanisms by which EGFR TKI resistance occurs 22, 52, 54. We were unable to detect downstream mutations in *PIK3CA*, *BRAF*, *KRAS*, *MAPK1* and others 22, 52, 54. Nevertheless, as *PIK3CA* alterations have been reported in a minority of chordomas, these could be used to stratify patients for future EGFR inhibitor clinical trials 23. *ERBB2* amplification, another reported resistance mechanism 22, 52, was not identified in the cell lines. Loss of heterozygosity for *PTEN* has been reported frequently in chordoma 19, 23, 24, 52, 53, 82, 83, 84; however, based on our *in vitro* results, it was not possible to predict response to EGFR inhibition based on PTEN expression. Other major causes of EGFR resistance may be explained by the activation of bypass signalling pathways such as MET 22, 52. It is therefore noteworthy that MET signalling was activated in the most resistant cell line, U‐CH2, and that a combination of the MET‐inhibitor crizotinib and the EGFR‐inhibitor sapitinib exhibited a synergistic effect on cell kill in this cell line. The absence of gene amplification and a *MET* mutation in this cell line leaves the mechanism of activation unanswered, although this has not been studied exhaustively, as MET can be activated by various other mechanisms, such as crosstalk with other receptor tyrosine kinases 85. As there are numerous resistance mechanisms to EGFR TKIs, many of which remain unexplained even in common cancers 22, it was beyond the scope of this project to pursue this further.

The collective data from this study show that EGFR inhibitors represent the group of compounds within our extensive screen that were most effective against chordoma cell growth. There have been reports that other therapeutic agents have been found to be active against chordoma but, ultimately, whether some patients with chordoma benefit from EGFR inhibitors alone or in combination with these other agents is likely only to be resolved in a clinical trial 3, 14, 29. We propose that such a study should involve in‐depth biological studies of the tumour samples pre‐ and post‐treatment, with the aim of explaining the mechanism by which some chordomas are primarily resistant or develop secondary resistance to EGFR inhibitors.

## Author contributions

AMF initiated and supervised the project; SS, MB, LC and MJ conducted the experiments; MB and SS outlined the screening cascade; MB conducted the data analysis; DHD and WJZ provided the GSK compounds and biochemical selectivity data and advised on data analysis and data preparation for publication; FT overlooked and advised on compound screening and data analysis; JAS was responsible for compound plate preparation; HY performed FISH analysis; NG performed immunohistochemistry; APL and NP supported data and NGS mutation analysis; APL conducted the MSigDB enrichment analysis; SjS advised on the clinical relevance of the findings; AL provided organizational support and clinical advice; SB and PM established the U‐CH7 primary culture; FA, PM and RT provided clinicopathological expertise; and SS and AMF wrote the manuscript. All authors read and approved the final version of the manuscript.

**SUPPLEMENTARY MATERIAL ON THE INTERNET**

The following supplementary material may be found in the online version of this article:

**Supplementary materials and methods**

**Figure S1.** *T* (*brachyury*) expression in the chordoma cell line panel

**Figure S2.** Western blot data for p‐EGFR and its downstream effectors in MUG‐Chor1, U‐CH7, U‐CH2 and JCH7

**Figure S3.** Western blots of U‐CH1, UM‐Chor1 and U‐CH2 in the absence of EGF spiking

**Figure S4.** Western blot analyses of U‐CH7, MUG‐Chor1 and JHC7 in the absence of EGF spiking

**Figure S5.** Endogenous/baseline status for the markers investigated

**Figure S6.** Apoptosis data for U‐CH7 and MUG‐Chor1

**Table S1.** STR profiles of the cell lines used in the experiments

**Table S2.** Summary of compounds included in the single‐concentration focused compound screen (*n =* 1097)

**Table S3.** Biochemical selectivity data for EGFR, ERBB2 and ERBB4 of selected EGFR/ERBB inhibitors (*n =* 14)

**Table S4.** Antibodies and conditions used for western blot analysis

**Table S5.** Inhibitory hit rates across all compound libraries included in the single‐concentration focused compound screen

**Table S6.** Enrichment in pathways of targets of non‐EGFR hit compounds.

**Table S7.** FISH data of the cell lines included in the study

## Supporting information

Supplementary materials and methods

**Figure S1.** T (brachyury) expression in the chordoma cell line panel. Real‐time quantitative PCR was conducted as described previously (see Supplementary materials and methods) 17; NAHDF, normal adult human dermal fibroblasts. All chordoma cell lines, but not the controls (NCI‐N87, NAHDF), were shown to express high levels of T

**Figure S2.** Western blot data for p‐EGFR and its downstream effectors in MUG‐Chor1, U‐CH7, U‐CH2 and JCH7 (EGF‐spiked, serum‐starved; for a detailed description, see legend to Figure 3)

**Figure S3.** Western blots of U‐CH1, UM‐Chor1 and U‐CH2 in the absence of EGF spiking; cells were treated with EGFR inhibitors for 4 h

**Figure S4.** Western blot analyses of U‐CH7, MUG‐Chor1 and JHC7 in the absence of EGF spiking

**Figure S5.** Endogenous/baseline status for the markers investigated

**Figure S6.** Apoptosis data for U‐CH7 and MUG‐Chor1 (for a detailed description, see legend to Figure 4)

**Table S1.** STR profiles of the cell lines used in the experiments

**Table S2.** Summary of compounds included in the single‐point focused compound screen (n = 1097)

**Table S3.** Biochemical selectivity data for EGFR, ERBB2 and ERBB4 of selected EGFR/ERBB inhibitors (n = 14)

**Table S4.** Antibodies and conditions used for western blot analysis

**Table S5.** Inhibitory hit rates across all compound libraries included in the single‐point focused compound screen

**Table S6.** Enrichment in pathways of targets of non‐EGFR hit compounds. Results for the key targets (n = 5) are displayed in Sheet 1; results, including targets of structurally closely related compounds (n = 13), are given in Sheet 2

**Table S7.** FISH data of the cell lines included in the study
