# Supplementary material for: EGFR inhibitors identified as a potential treatment for chordoma in a focused compound screen
Source: J Pathol. 2016 May 31;239(3):320–34. doi: 10.1002/path.4729 (PMC4922416; doi:10.1002/path.4729)
Supplement: Supplementary file 1 — Supplementary materials and methods [file PATH-239-320-s011.doc]

***Supplementary Materials and Methods***

1. ***Establishing a new chordoma cell line: U-CH7.*** U-CH7 was established from samples obtained from a patient at Royal National Orthopaedic Hospital Stanmore, UK (RNOH). Tumour tissue was acquired from a primary sacral chordoma (90 x 75 x 60 mm) of a 33 year-old Caucasian male after surgery in September 2011. Partial sacrectomy was followed by adjuvant radiotherapy. Histological diagnosis confirmed conventional chordoma not otherwise specified (NOS). The patient is still alive with no evidence of chordoma after 50 months of follow-up. The resected tumour tissue was submitted to Dr. Brüderlein, Ulm, Germany for establishment of the primary cultures according to her previously established methods [30, 31]. After the culture had reached a steady state with approximately 30–40% of physaliferous cells it was returned to the UCL Cancer Institute, London, UK. Cells were cultured in 4:1 Iscove’s Modified Dulbecco’s Medium (IMDM) (Gibco, Paisley, Renfrewshire, UK): RPMI 1640 (Gibco) with 10% FBS (Life Technologies, Inchinnan, Renfrewshire, UK), and 1% Pen/Strep (Gibco). The cell culture was successfully passaged for 55 times over a 36 month period and regular testing showed they remained negative for mycoplasma. STR analysis (DNA Diagnostic Center, London, UK) confirmed the origin of U-CH7 from the tumour from which it was originally derived ***(Suppl. Table 1B)***. U-CH7 was recently validated by Vala Sciences (San Diego, CA, USA) on behalf of the Chordoma Foundation (detailed data available from the authors and the Chordoma Foundation): the cell line expresses high levels of *T (brachyury)* ***(Suppl. Figure 1)***, and will be made available presently as a bioresource from the American Type Culture Collection (ATCC).
2. ***Tissue culture methods.*** Chordoma cell lines were cultured according to previously reported conditions [29-31, 33] (<http://www.chordomafoundation.org/>). Cell lines were either obtained from our collaborators (U-CH1, U-CH2, MUG-Chor1) or from the Chordoma Foundation (JHC7, U-CH10, UM-Chor1). U-CH7 was established as described above. Normal adult human dermal fibroblasts were cultured in Fibroblast Basal Medium (ATCC® PCS-201-030™) and supplemented with Fibroblast Growth Kit-Low serum (ATCC® PCS-201-041™). NCI-N87 cells were grown in RPMI 1640 (Gibco) with 10% FBS (Life Technologies), and 1% Pen/Strep (Gibco). The cells were plated at the following densities: 156 cells/mm^2^ (U-CH1, U-CH7, U-CH10, MUG-Chor1, JHC7, UM-Chor1, and NCI-N87), 78 cells/mm^2^ (U-CH2) and 14 cells/mm^2^ (human dermal fibroblasts).
3. ***Protein Extraction and Western Blot Analysis (WB).*** Cells were lysed with RIPA buffer containing EDTA-free Protease (Roche) and Phosphatase inhibitors (Sigma-Aldrich, Gillingham, Dorset, UK). Total protein concentration was determined using a Pierce™ BCA Protein Assay Kit (Thermo Scientific). Proteins were resolved on an acrylamide gel (Criterion™ TGX (Tris-Glycine eXtended) Stain-Free™ Any KD, Bio-Rad Laboratories Ltd., Hertfordshire, UK) and blotted on nitrocellulose Whatman® Protran® Membranes (GE Healthcare Life Sciences, Buckinghamshire, UK). Filters were developed using an Odyssey Infrared Imaging System (LI-COR Biotechnology, Cambridge, Cambridgeshire, UK). Experiments were performed n=2 (min.).
4. ***Combination Study of Sapitinib with the MET Inhibitor Crizotinib.*** U-CH2 cells were seeded in 70 µl of medium and tested in a 7-point dose response matrix. Profiling was conducted from a maximum concentration (1 µM) in a 1:3 serial dilution. Raw data were corrected for background (media only, 16 replicates per plate). Synergy was determined using the CalcuSyn software version 2.1 (Biosoft, Cambridge, Cambridgeshire, UK). A combination index (CI) was calculated and evaluated as synergistic (CI < 0.9), additive (CI = 0.9 to 1.1), and antagonistic (CI > 1.1) [86]. Statistical analysis was conducted using the Students t-test in GraphPad version 6.0 (Prism, CA). P values ≤ 0.05 (**) were considered significant.
5. ***Real-time quantitative PCR.*** RNA was extracted from frozen cell line pellets using the miRNeasy Mini Kit (Qiagen GmbH, Hilden, Germany), quantified using Nanodrop® spectrophotometry (Thermo Fisher Scientific, Wilmington, DE, USA), and transcribed into cDNA (500 µg of RNA) applying the High Capacity cDNA Reverse Transcription Kit (Applied Biosystems, CA, USA) according to the manufacturers’ instructions. Quantitative real-time PCR (q-RT-PCR) was carried out using Fast SYBR^®^ Green MasterMix (Applied Biosystems) according to the manufacturer’s instructions. Previously published primers were used: *T (brachyury)*: 5’- CCCGTCTCCTTCAGCAAAGTC-3’ forward; 5’-TGGATTCGAGGCTCATACTTATGC-3’ reverse; *GAPDH*: 5’- GGAGTCAACGGATTTGGTCGTA-3’ forward; *GAPDH* reverse: 5’- GGCAACAATATCCACTTTACCAGAGT-3’ reverse. Water samples were included as controls. For analysis of *T* expression in human chordoma cell lines, the 2^-ΔCT^ method was applied, normalised to *GAPDH* expression [17]. U-CH1 was set as the reference for comparison between *T*-expressing chordoma cell lines.
6. ***Immunohistochemistry (IHC).*** Antibodies against PTEN (clone 6H2.1, mouse, Dako, Agilent Technologies, Glostrup, Denmark), phospho (p)-MET (Tyr1234/1235, clone D26, rabbit, Cell Signaling Technology, Leiden, The Netherlands), and E-Cadherin (clone NCH-38, mouse, Dako) were used after pretreatment with Bond TM Epitope Retrieval Solution 2 (Leica Biosystems, Milton Keynes, Buckinghamshire, UK). All antibodies were used in a 1:100 dilution. Immunohistochemical sections were analysed semiquantitatively as reported previously [18]: 0-absence of immunoreactivity; 1-“weak”, if the staining intensity was weaker than the positive control; 2-“moderate”, if the staining was as intense as the positive control; 3-“strong”, if the staining intensity was stronger than the positive control [18]. A minimum of 10% of immunoreactive cells was required to consider a sample as immunoreactive. 10-25% of immunoreactive cells: +; 26-49% of immunoreactive cells: ++; ≥ 50% of immunoreactive cells: +++ [18].
7. ***FISH Analysis.*** EGFR/ERBB2/MET status was scored as the average number of EGFR/ERBB2/MET red/green/green signals per nucleus and as the ratio between EGFR/ERBB2/MET red/green/green signals and CEP7/CEP17/CEN7 green/red/red signals. The FISH-positive group comprised two categories: high-level polysomy of chromosome 7/17/7 (≥ 4 copies of the gene of interest and CEP7/17/7 per cell in ≥ 40% of cells) and amplification (≥ 15 gene copies per cell in ≥ 10% of analysed cells regardless of the number of CEP7/17/7 signals). The FISH-negative group included tumours with low-level polysomy (≥ 2 copies of the gene of interest and CEP7/17/7 per cell in ≤ 40% of cells, and 3 copies of the gene of interest per cell in ≥ 40% of cells) and disomy (≤ 2 copies of the gene of interest and CEP7/17/7 in ≥ 90% of the cells) [18].
8. ***Mutations in Cancer Gene Hotspots.*** Sequencing was performed at UCL Advanced Diagnostics - Sarah Cannon Research Laboratories, London, UK using the Ion Torrent Personal Genome Machine (PGM) (Life Technologies) and Ion PGM^TM^ Sequencing 200 Kit v2 chemistry (Thermo Fisher), as well as a 318v2 chip (Thermo Fisher). Data were analysed using Torrent suite v4.0.2 and Variant Caller v4.0 (r76860) (Thermo Fisher). An in-house developed script was used to group the Variant Caller output into categories (UCL Advanced Diagnostics - Sarah Cannon Research Laboratories). Only variants with a frequency of >2.5% in not less than 100 high quality unbiased reads were qualified as being “detected”.
9. ***In vivo Studies.*** Preclinical studies were conducted through the Chordoma Foundation Drug Screening Pipeline at South Texas Accelerated Research Therapeutics (START) under International Animal Care and Use Committee-approved protocols. Antitumour activity was tested in 2 xenografts: a xenograft of the U-CH1 cell line [32] and the patient-derived xenograft SF8894 [43]. For all models, athymic nude mice (Charles River Laboratories Inc., Wilmington, MA, USA) between 6-8 weeks of age were implanted subcutaneously with tumour fragments from host animals. Once tumours reached approximately 150-250 mm^3^, animals were matched by tumour volume (TV) and randomised to the control and the treatment group. The treatment group was dosed orally with sapitinib (AstraZeneca) 25 mg/kg twice daily (50 mg/kg total dose) for a minimum of 4 weeks (28 days for SF8894, and 42 days for U-CH1). Initial dosing began at Day 0. Animals were observed daily and weighed twice a week. TV and animal weight data were collected electronically using a digital caliper and scale; tumour dimensions were converted to tumour volume using the formula TV (mm^3^) = width^2^ (mm^2^) x length (mm) x 0.52. Endpoints were a mean control TV of approximately 1–2 cm^3^. Percent tumour growth inhibition values were calculated and reported for the treatment group versus the control group using initial and final tumour measurements. Statistical analyses were performed using a two-way analysis of variance (ANOVA) followed by the Dunnett’s multiple comparisons test.
