# Supplementary figures and images for: EGFR inhibitors identified as a potential treatment for chordoma in a focused compound screen
Source: J Pathol. 2016 May 31;239(3):320–34. doi: 10.1002/path.4729 (PMC4922416; doi:10.1002/path.4729)

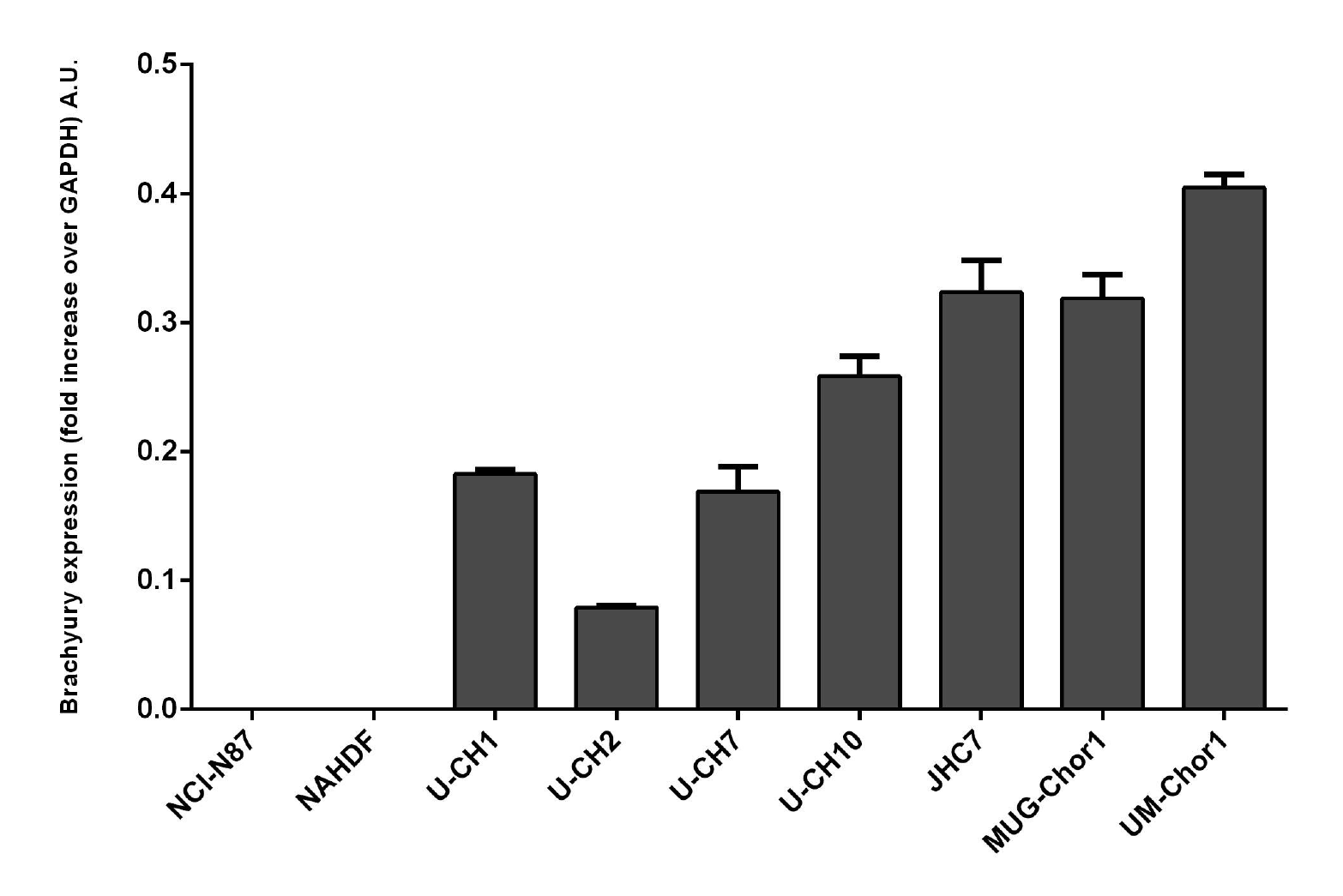

Supplement: Supplementary file 2 — Figure S1. T (brachyury) expression in the chordoma cell line panel. Real‐time quantitative PCR was conducted as described previously (see Supplementary materials and methods) 17; NAHDF, normal adult human dermal fibroblasts. All chordoma cell lines, but not the controls (NCI‐N87, NAHDF), were shown to express high levels of T [file PATH-239-320-s012.tif]

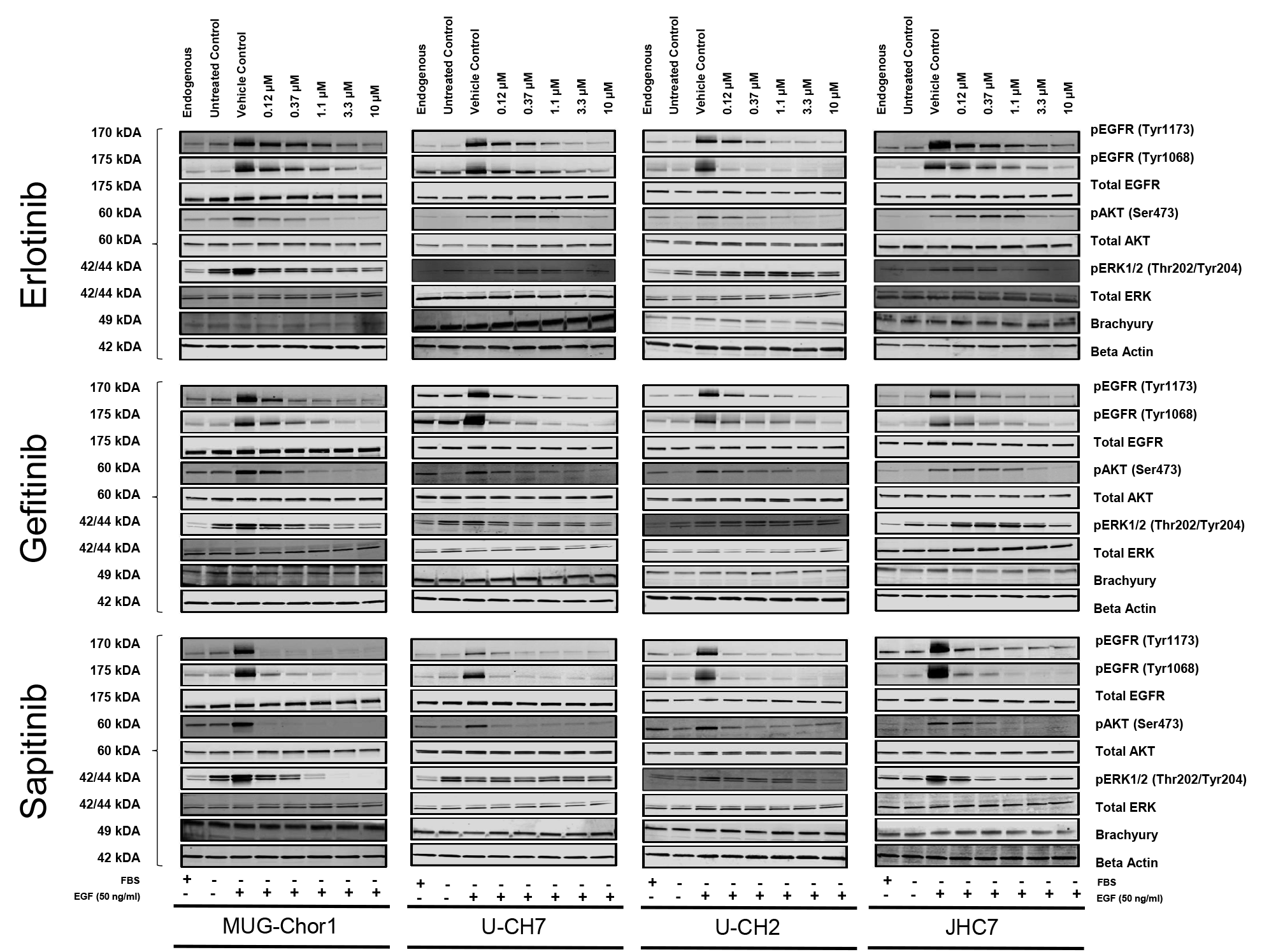

Supplement: Supplementary file 3 — Figure S2. Western blot data for p‐EGFR and its downstream effectors in MUG‐Chor1, U‐CH7, U‐CH2 and JCH7 (EGF‐spiked, serum‐starved; for a detailed description, see legend to Figure 3) [file PATH-239-320-s004.tif]

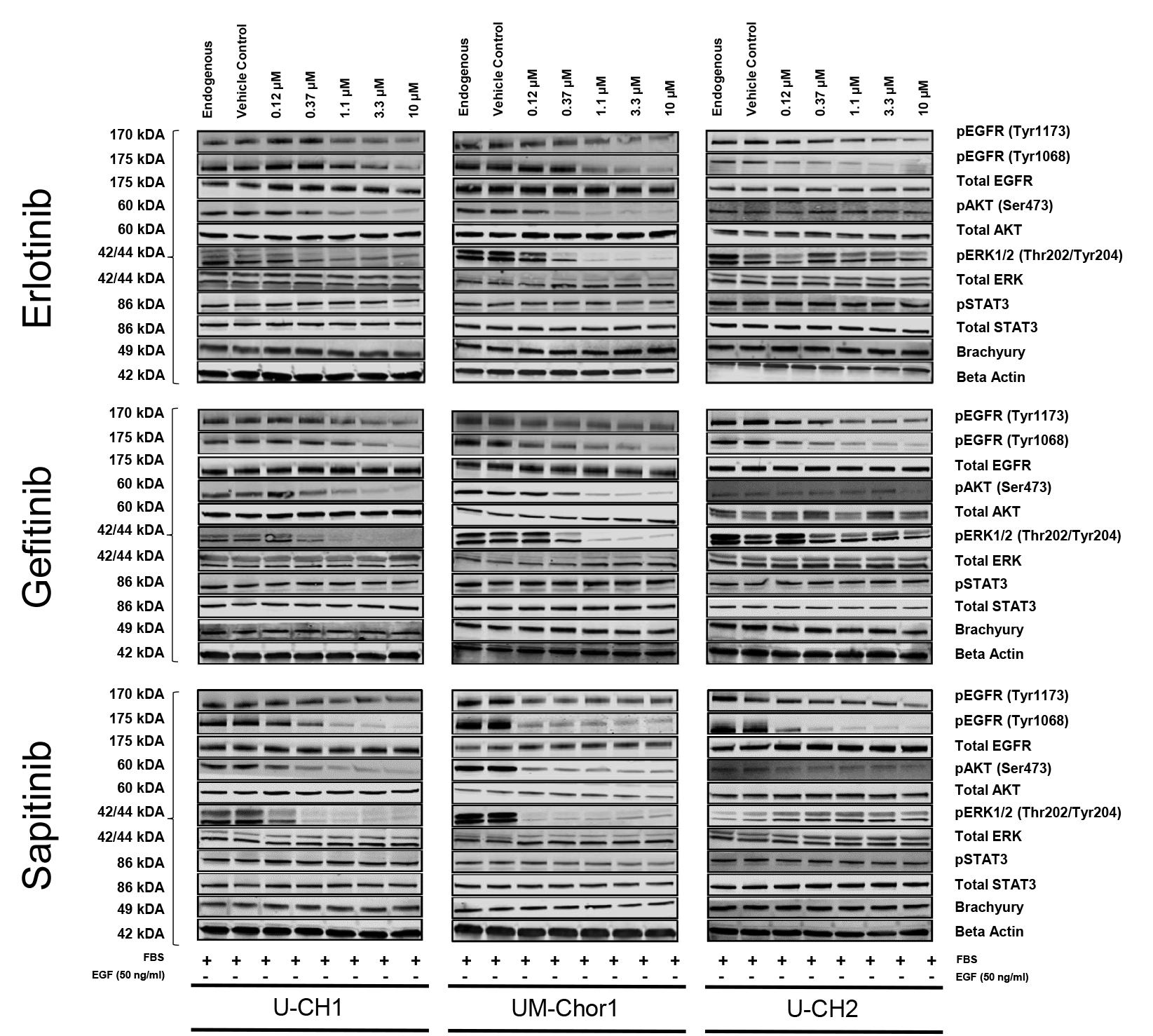

Supplement: Supplementary file 4 — Figure S3. Western blots of U‐CH1, UM‐Chor1 and U‐CH2 in the absence of EGF spiking; cells were treated with EGFR inhibitors for 4 h [file PATH-239-320-s001.tif]

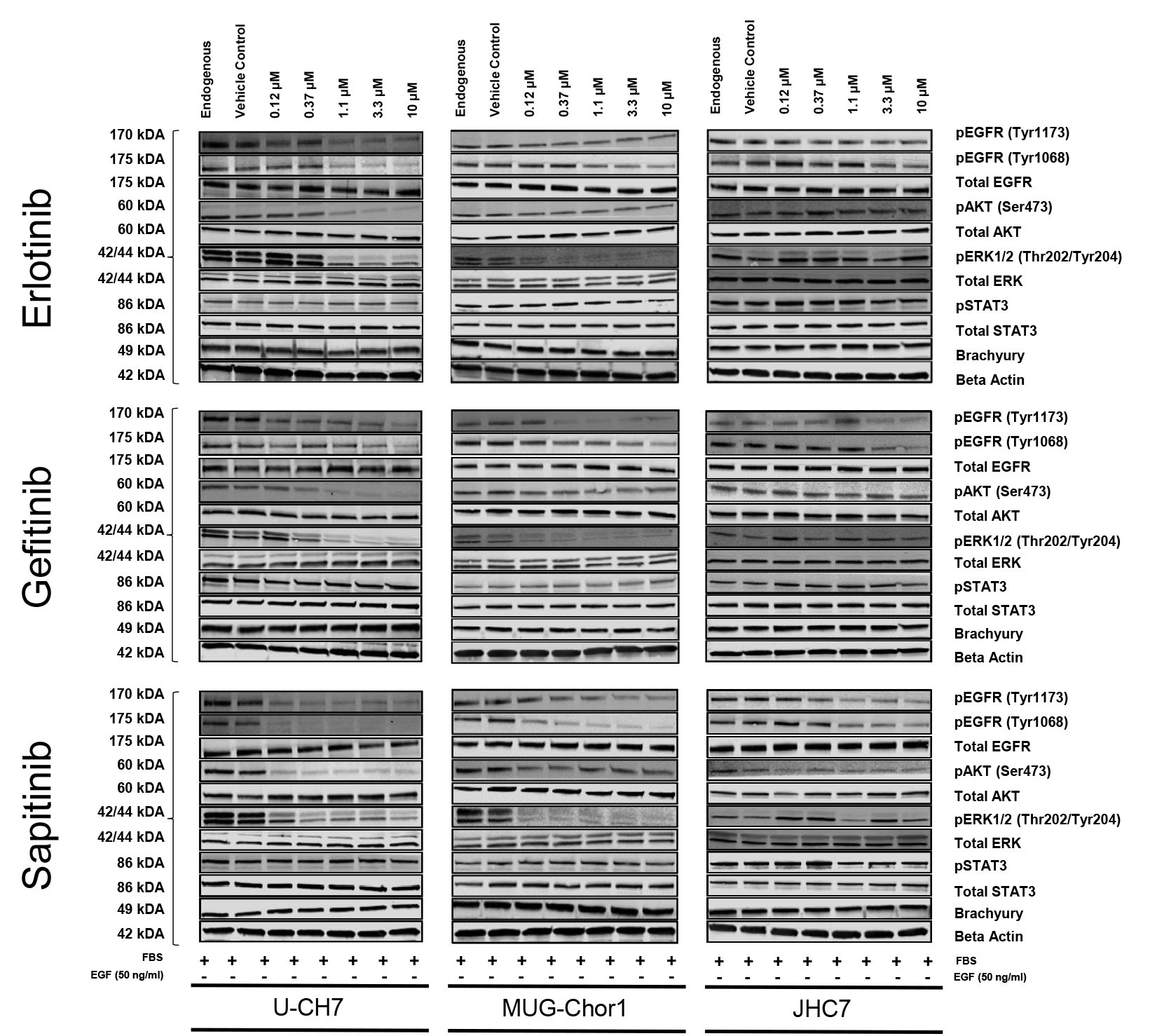

Supplement: Supplementary file 5 — Figure S4. Western blot analyses of U‐CH7, MUG‐Chor1 and JHC7 in the absence of EGF spiking [file PATH-239-320-s008.tif]

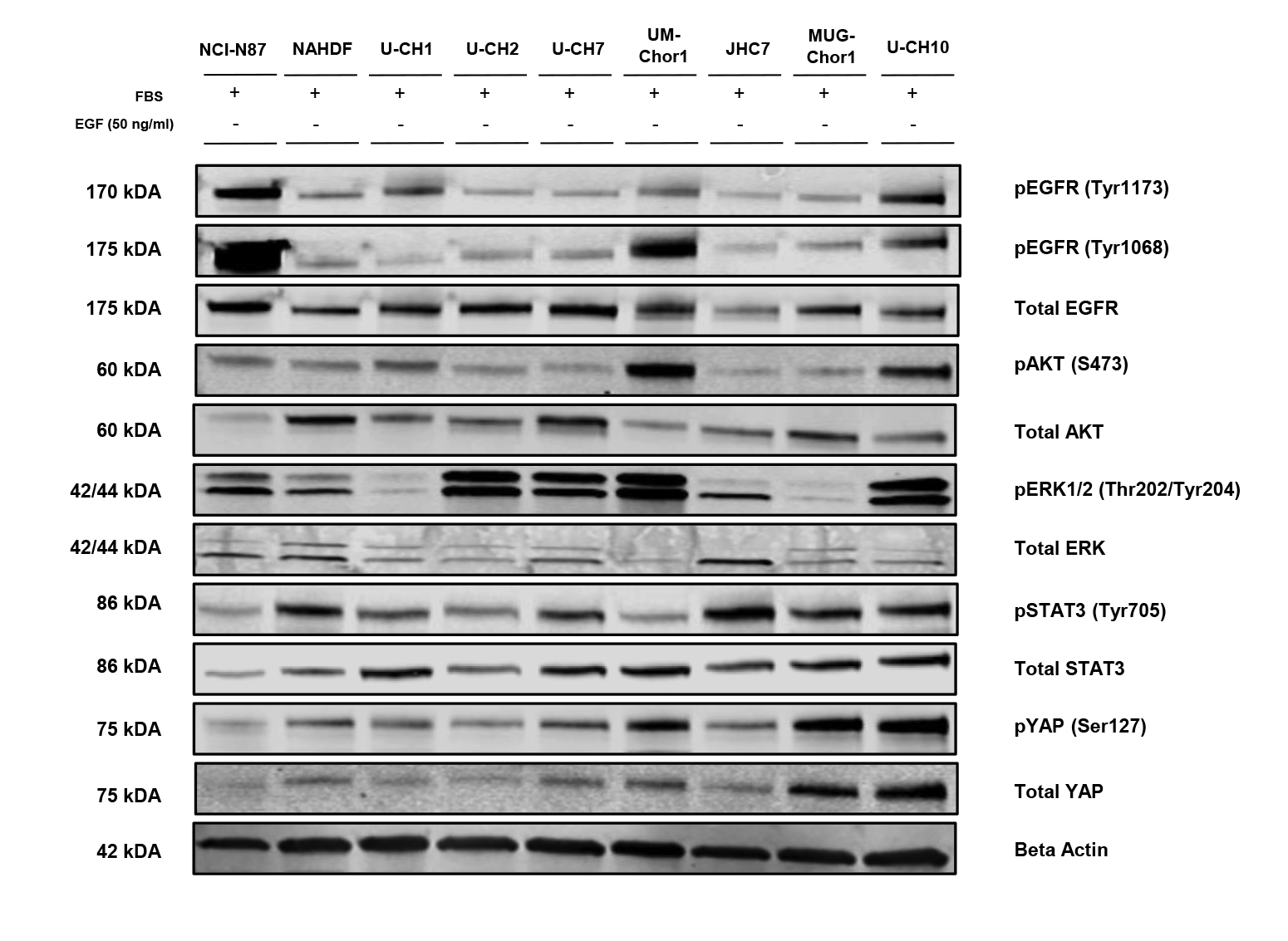

Supplement: Supplementary file 6 — Figure S5. Endogenous/baseline status for the markers investigated [file PATH-239-320-s009.tif]

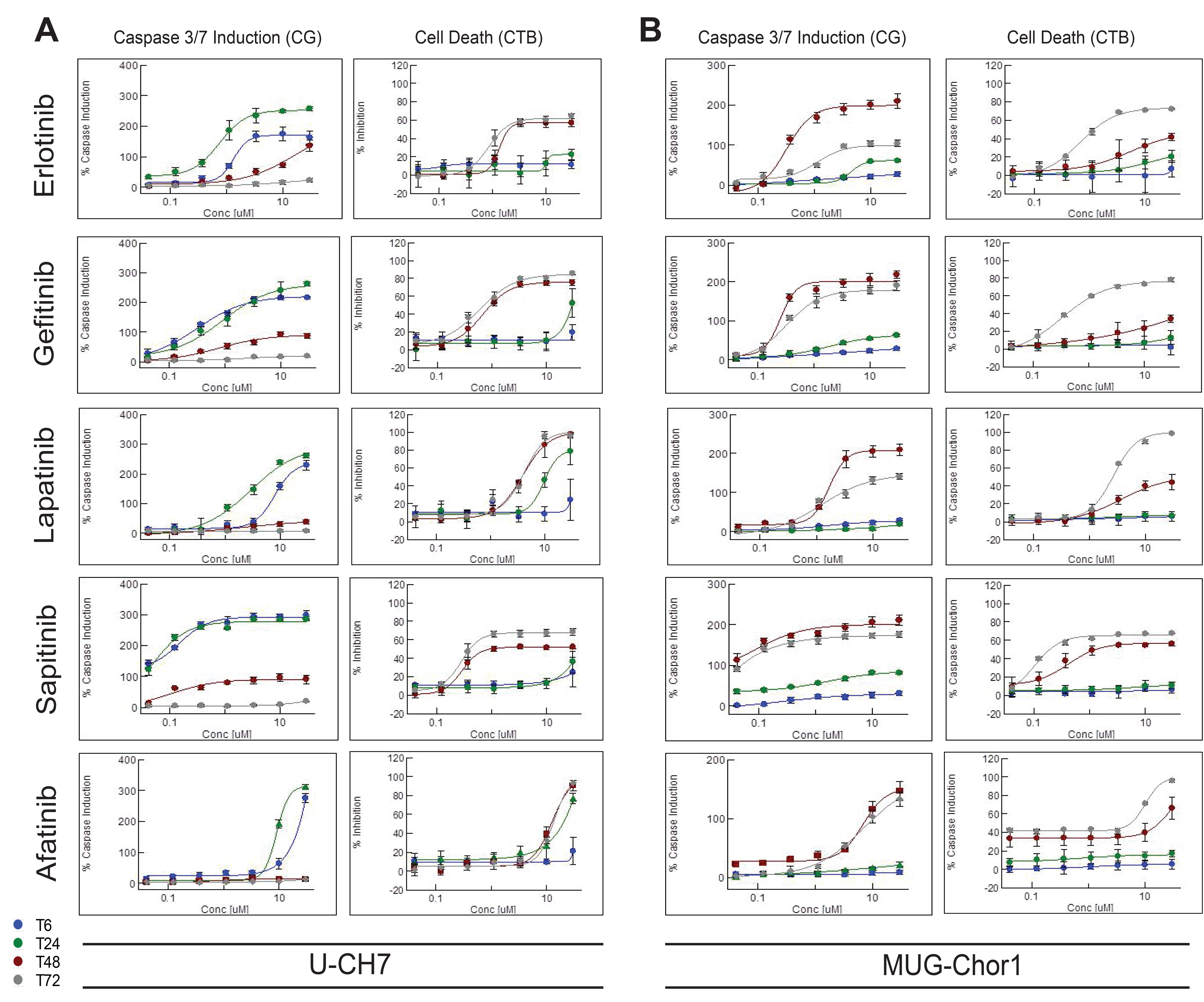

Supplement: Supplementary file 7 — Figure S6. Apoptosis data for U‐CH7 and MUG‐Chor1 (for a detailed description, see legend to Figure 4) [file PATH-239-320-s010.tif]
