# Supplementary material for: EGFR inhibitors identified as a potential treatment for chordoma in a focused compound screen
Source: J Pathol. 2016 May 31;239(3):320–34. doi: 10.1002/path.4729 (PMC4922416; doi:10.1002/path.4729)
Supplement: Supplementary file 8 — Table S1. STR profiles of the cell lines used in the experiments [file PATH-239-320-s013.docx]

**Table S1A.** STR profiles of cell lines used in the experiments 2015

| **STR analysis** | **U-CH1** | | **U-CH2** | | **U-CH7** | | **U-CH10** | | **JCH7** | | **MUG-Chor1** | | **UM-Chor1** | | **NCI-N87** | |
| --- | --- | --- | --- | --- | --- | --- | --- | --- | --- | --- | --- | --- | --- | --- | --- | --- |
| **Marker** | **Allele 1** | **Allele 2** | **Allele 1** | **Allele 2** | **Allele 1** | **Allele 2** | **Allele 1** | **Allele 2** | **Allele 1** | **Allele 2** | **Allele 1** | **Allele 2** | **Allele 1** | **Allele 2** | **Allele 1** | **Allele 2** |
| D3S1358 | 15 |  | 17 |  | 17 |  | 15 | 16 | 17 |  | 14 | 17 | 18 |  | 14 |  |
| TH01 | 7 |  | 9.3 |  | 7 |  | 8 | 9 | 6 | 8 | 9.3 |  | 7 | 9.3 | 7 | 9 |
| D21S11 | 28 | 29 | 29 | 30 | 30 | 33.2 | 29 | 31 | 27 | 31.2 | 29 | 33.2 | 27 | 31 | 30 |  |
| D18S51 | 15 |  | 12 | 18 | 16 |  | 13 |  | 12 |  | 17 | 23 | 14 |  | 17 |  |
| Penta E | 7 | 10 | 12 | 15 | 12 | 15 | 18 |  | 15 |  | 5 | 12 | 7 |  | 5 |  |
| D5S818 | 11 | 12 | 10 | 11 | 12 | 13 | 11 |  | 13 |  | 11 | 12 | 9 | 13 | 12 | 13 |
| D13S137 | 11 | 13 | 11 |  | 10 |  | 9 | 13 | 11 |  | 11 |  | 12 |  | 8 | 11 |
| D7S820 | 9 | 12 | 8 | 12 | 10 | 11 | 10 |  | 7 | 10 | 8 | 11 | 11 |  | 10 | 11 |
| D16S539 | 12 | 13 | 12 |  | 10 | 12 | 11 | 13 | 11 |  | 11 | 14 | 12 |  | 9 | 13 |
| CSF1PO | 10 | 11 | 11 | 12 | 12 |  | 10 | 11 | 11 |  | 11 |  | 11 | 12 | 8 | 12 |
| Penta D | 11 |  | 12 | 13 | 12 | 13 | 9 | 13 | 6 | 11 | 13 |  | 8 | 9 | 12 |  |
| AMEL | X | Y | X |  | X | Y | X |  | X |  | X |  | X | Y | X | Y |
| vWA | 17 |  | 17 |  | 16 | 17 | 16 |  | 17 |  | 15 |  | 15 |  | 15 | 16 |
| D8S1179 | 10 | 15 | 13 |  | 14 | 16 | 8 | 13 | 13 | 14 | 11 | 12 | 12 | 13 | 14 | 15 |
| TPOX | 8 | 11 | 8 |  | 8 | 11 | 11 |  | 10 | 11 | 8 |  | 8 | 9 | 9 | 11 |
| FGA | 20 | 21 | 21 | 22.2 | 22 | 24 | 24 |  | 21 | 23 | 21 | 26 | 19 | 23 | 20 | 21 |
| D19S433 | 14 |  | 15.2 | 17 | 13 | 14 | 13 | 15 | 13 | 13.2 | 13 | 14 | 14 | 16 | 14 | 14.2 |
| D2S1338 | 17 | 18 | 24 |  | 23 |  | 19 |  | 18 | 20 | 18 | 20 | 18 | 20 | 23 | 24 |

**Table S1B.** STR profiles of cell lines used in the experiments 2014

| **STR analysis** | **U-CH1** | | **U-CH2** | | **MUG-Chor1** | | **U-CH7**  **cell line UCL** | | **U-CH7**  **primary tumour** | |
| --- | --- | --- | --- | --- | --- | --- | --- | --- | --- | --- |
| **Marker** | **Allele 1** | **Allele 2** | **Allele 1** | **Allele 2** | **Allele 1** | **Allele 2** | **Allele 1** | **Allele 2** | **Allele 1** | **Allele 2** |
| D3S1358 | 15 |  | 17 |  | 14 | 17 | 17 |  | 16 | 17 |
| TH01 | 7 |  | 9.3 |  | 9.3 |  | 7 |  | 7 |  |
| D21S11 | 28 | 29 | 29 | 30 | 29 | 33.2 | 30 | 33.2 | 30 | 33.2 |
| D18S51 | 15 |  | 12 | 18 | 17 | 23 | 16 |  | 16 | 18 |
| Penta E | 7 | 10 | 12 | 15 | 5 | 12 | 12 | 15 | 12 | 15 |
| D5S818 | 11 | 12 | 10 | 11 | 11 | 12 | 12 | 13 | 12 | 13 |
| D13S137 | 11 | 13 | 11 |  | 11 |  | 10 |  | 10 | 12 |
| D7S820 | 9 | 12 | 8 | 12 | 8 | 11 | 10 | 11 | 10 | 11 |
| D16S539 | 12 | 13 | 12 |  | 11 | 14 | 10 | 12 | 10 | 12 |
| CSF1PO | 10 | 11 | 11 | 12 | 11 |  | 12 |  | 12 |  |
| Penta D | 11 |  | 12 | 13 | 13 |  | 12 | 13 | 12 | 13 |
| AMEL | X | Y | X |  | X |  | X | Y | X | Y |
| vWA | 17 |  | 17 |  | 15 |  | 16 | 17 | 16 | 17 |
| D8S1179 | 10 | 15 | 13 |  | 11 | 12 | 14 | 16 | 14 | 16 |
| TPOX | 8 | 11 | 8 |  | 8 |  | 8 | 11 | 8 | 11 |
| FGA | 20 | 21 | 21 | 22.2 | 21 | 26 | 22 | 24 | 22 | 24 |
| D19S433 | 14 |  | 15.2 | 17 | 13 | 14 | 13 | 14 | 13 | 14 |
| D2S1338 | 17 | 18 | 24 |  | 18 |  | 23 |  | 23 |  |
