# Supplementary material for: EGFR inhibitors identified as a potential treatment for chordoma in a focused compound screen
Source: J Pathol. 2016 May 31;239(3):320–34. doi: 10.1002/path.4729 (PMC4922416; doi:10.1002/path.4729)
Supplement: Supplementary file 9 — Table S2. Summary of compounds included in the single‐point focused compound screen (n = 1097) [file PATH-239-320-s014.docx]

**Table S2.** Summary of compounds included in the single-point focused compound screen (*n* = 1097)

| **No.** | **Supplier ID or commercial name** | **Library** | **Supplier** | **U-CH1** | | **MUG-Chor1** | | **U-CH2** | |
| --- | --- | --- | --- | --- | --- | --- | --- | --- | --- |
|  |  |  |  | **Mean M%I** | **SD** | **Mean M%I** | **SD** | **Mean M%I** | **SD** |
| 1 | S1065* | Anticancer | SelleckChem | 69 | 6 | 56 | 5 | 64 | 9 |
| 2 | S1009* | Anticancer | SelleckChem | 78 | 5 | 37 | 10 | 61 | 20 |
| 3 | S1037 | Anticancer | SelleckChem | 35 | 4 | 10 | 15 | 0 | 19 |
| 4 | S1638 | Anticancer | SelleckChem | 26 | 2 | –4 | 8 | 14 | 3 |
| 5 | S1215* | Anticancer | SelleckChem | 87 | 11 | 104 | 0 | 101 | 1 |
| 6 | S1141 | Anticancer | SelleckChem | 4 | 4 | 15 | 5 | 8 | 6 |
| 7 | S1142* | Anticancer | SelleckChem | 63 | 5 | 75 | 3 | 86 | 3 |
| 8 | S1003* | Anticancer | SelleckChem | 57 | 30 | 29 | 5 | 9 | 7 |
| 9 | S1004 | Anticancer | SelleckChem | 15 | 7 | 10 | 10 | 2 | 7 |
| 10 | S1208* | Anticancer | SelleckChem | 94 | 2 | 42 | 6 | 67 | 4 |
| 11 | S1490* | Anticancer | SelleckChem | 56 | 38 | 59 | 9 | 83 | 7 |
| 12 | S1060* | Anticancer | SelleckChem | 80 | 19 | 71 | 6 | 76 | 7 |
| 13 | S1555* | Anticancer | SelleckChem | 82 | 2 | 59 | 10 | 76 | 5 |
| 14 | S1085* | Anticancer | SelleckChem | 80 | 23 | 83 | 5 | 27 | 15 |
| 15 | S1010 | Anticancer | SelleckChem | 15 | 1 | 14 | 11 | 30 | 12 |
| 16 | S1013* | Anticancer | SelleckChem | 50 | 69 | 98 | 4 | 96 | 2 |
| 17 | S1084 | Anticancer | SelleckChem | 12 | 9 | –7 | 8 | 1 | 31 |
| 18 | S1261 | Anticancer | SelleckChem | 23 | 2 | –6 | 13 | –2 | 7 |
| 19 | S1018 | Anticancer | SelleckChem | –13 | 43 | 10 | 13 | 8 | 4 |
| 20 | S1194 | Anticancer | SelleckChem | 20 | 2 | 36 | 7 | 7 | 11 |
| 21 | S1021 | Anticancer | SelleckChem | 9 | 12 | 11 | 11 | 33 | 4 |
| 22 | S1200* | Anticancer | SelleckChem | 58 | 59 | –12 | 12 | 8 | 2 |
| 23 | S1022* | Anticancer | SelleckChem | 33 | 39 | 32 | 7 | 53 | 6 |
| 24 | S1148* | Anticancer | SelleckChem | 59 | 39 | 49 | 8 | 64 | 2 |
| 25 | S1225 | Anticancer | SelleckChem | 30 | 15 | 36 | 11 | 45 | 1 |
| 26 | S1120* | Anticancer | SelleckChem | 20 | 32 | 28 | 6 | 56 | 4 |
| 27 | S1230* | Anticancer | SelleckChem | 76 | 3 | 40 | 10 | 72 | 7 |
| 28 | S1082* | Anticancer | SelleckChem | 47 | 9 | 47 | 6 | 67 | 13 |
| 29 | S1360* | Anticancer | SelleckChem | 48 | 3 | 27 | 5 | 53 | 4 |
| 30 | S1026 | Anticancer | SelleckChem | 27 | 6 | 17 | 12 | 12 | 3 |
| 31 | S1028* | Anticancer | SelleckChem | 64 | 7 | 37 | 9 | 16 | 17 |
| 32 | S1361 | Anticancer | SelleckChem | –9 | 28 | 4 | 7 | 2 | 4 |
| 33 | S1220* | Anticancer | SelleckChem | 25 | 33 | 6 | 7 | 85 | 2 |
| 34 | S1220* | Anticancer | SelleckChem | 43 | 32 | 67 | 7 | 4 | 4 |
| 35 | S1035 | Anticancer | SelleckChem | 34 | 20 | 8 | 16 | 23 | 18 |
| 36 | S1487* | Anticancer | SelleckChem | 36 | 9 | 26 | 5 | 55 | 6 |
| 37 | S1185 | Anticancer | SelleckChem | 6 | 1 | 5 | 4 | 10 | 7 |
| 38 | S1515* | Anticancer | SelleckChem | 80 | 25 | 86 | 5 | 64 | 14 |
| 39 | S1040 | Anticancer | SelleckChem | 13 | 3 | 11 | 13 | 0 | 13 |
| 40 | S1118 | Anticancer | SelleckChem | 5 | 2 | 6 | 11 | –1 | 9 |
| 41 | S1236 | Anticancer | SelleckChem | 28 | 30 | 3 | 12 | 0 | 6 |
| 42 | S1130 | Anticancer | SelleckChem | 37 | 10 | 17 | 3 | 48 | 6 |
| 43 | S1223* | Anticancer | SelleckChem | 27 | 35 | 45 | 5 | 65 | 7 |
| 44 | 567805, Src kinase inhibitor I | Calbiochem | Merck Millipore | 23 | 4 | 35 | 9 | 23 | 5 |
| 45 | 658552, AG 1478* | Calbiochem | Merck Millipore | 61 | 3 | 35 | 11 | 24 | 9 |
| 46 | 365250, Go6976 | Calbiochem | Merck Millipore | 28 | 12 | 15 | 14 | 24 | 5 |
| 47 | 371970, HA-1077 | Calbiochem | Merck Millipore | 9 | 8 | 18 | 8 | 23 | 11 |
| 48 | 203290, GF103209X | Calbiochem | Merck Millipore | 3 | 9 | 12 | 16 | 29 | 6 |
| 49 | 688000, Y-27632 | Calbiochem | Merck Millipore | 2 | 12 | 9 | 9 | 11 | 10 |
| 50 | 513000, PD 98059 | Calbiochem | Merck Millipore | 8 | 6 | 21 | 13 | 17 | 9 |
| 51 | 658390, Tyrphostin 1 | Calbiochem | Merck Millipore | 7 | 4 | 17 | 10 | 15 | 6 |
| 52 | 658550, AG 1295 | Calbiochem | Merck Millipore | 20 | 7 | 13 | 3 | 18 | 6 |
| 53 | 658401, AG 490 | Calbiochem | Merck Millipore | 11 | 7 | 6 | 3 | 11 | 7 |
| 54 | 440202, LY 294002 | Calbiochem | Merck Millipore | 32 | 4 | 14 | 11 | 41 | 10 |
| 55 | 371963, H-89 | Calbiochem | Merck Millipore | 14 | 8 | 14 | 8 | 25 | 2 |
| 56 | 422708, KN-93 | Calbiochem | Merck Millipore | 7 | 3 | 7 | 8 | 12 | 19 |
| 57 | 559388, SB-202190 | Calbiochem | Merck Millipore | –11 | 9 | –11 | 7 | 11 | 14 |
| 58 | 317200, DMBI | Calbiochem | Merck Millipore | 6 | 9 | –2 | 9 | 13 | 7 |
| 59 | 658551, AG 1296 | Calbiochem | Merck Millipore | 11 | 15 | 9 | 4 | 18 | 4 |
| 60 | 196870, BAY 11-7082 | Calbiochem | Merck Millipore | 4 | 6 | 18 | 12 | 10 | 12 |
| 61 | 420119, SP 600125 | Calbiochem | Merck Millipore | 9 | 6 | 10 | 6 | 19 | 5 |
| 62 | 402085, Indirubin-3'-monoxime | Calbiochem | Merck Millipore | 17 | 5 | –4 | 21 | 8 | 3 |
| 63 | 422000, Kenpaullone | Calbiochem | Merck Millipore | 5 | 9 | 3 | 2 | 14 | 3 |
| 64 | 260961, NU7026 | Calbiochem | Merck Millipore | 12 | 2 | 12 | 8 | 19 | 5 |
| 65 | 328007, FR180204 | Calbiochem | Merck Millipore | –5 | 7 | 15 | 9 | 14 | 4 |
| 66 | 328008 | Calbiochem | Merck Millipore | 1 | 1 | 16 | 6 | 12 | 5 |
| 67 | 676489 | Calbiochem | Merck Millipore | –5 | 11 | 13 | 9 | 11 | 6 |
| 68 | 365251, Go6983 | Calbiochem | Merck Millipore | 13 | 8 | 13 | 3 | 27 | 5 |
| 69 | 401481 | Calbiochem | Merck Millipore | 15 | 19 | 33 | 9 | 40 | 2 |
| 70 | 217696, Cdk1 inhibitor, CGP74514A* | Calbiochem | Merck Millipore | 4 | 20 | 9 | 1 | 61 | 9 |
| 71 | 203600, Bohemine | Calbiochem | Merck Millipore | –11 | 37 | 7 | 6 | 9 | 11 |
| 72 | 234503, Compound 52 | Calbiochem | Merck Millipore | –15 | 36 | 7 | 4 | 11 | 1 |
| 73 | 527450, PKR inhibitor* | Calbiochem | Merck Millipore | 36 | 25 | 66 | 16 | 65 | 6 |
| 74 | 528100, PI-103* | Calbiochem | Merck Millipore | 84 | 6 | 55 | 8 | 65 | 9 |
| 75 | 118500 | Calbiochem | Merck Millipore | 6 | 10 | –6 | 7 | 13 | 11 |
| 76 | 118501 | Calbiochem | Merck Millipore | 13 | 8 | 22 | 5 | 20 | 9 |
| 77 | 121767, AG 1024 | Calbiochem | Merck Millipore | 14 | 8 | 11 | 17 | 12 | 5 |
| 78 | 121790, AGL 2043 | Calbiochem | Merck Millipore | –7 | 14 | 11 | 5 | 17 | 18 |
| 79 | 124011* | Calbiochem | Merck Millipore | 80 | 7 | 21 | 11 | 38 | 60 |
| 80 | 124012, Triciribine | Calbiochem | Merck Millipore | 14 | 6 | 30 | 1 | 2 | 3 |
| 81 | 124018 | Calbiochem | Merck Millipore | 27 | 3 | 11 | 12 | 34 | 1 |
| 82 | 124020 | Calbiochem | Merck Millipore | –5 | 8 | 17 | 8 | 19 | 4 |
| 83 | 126870, Alsterpaullone | Calbiochem | Merck Millipore | 6 | 22 | 25 | 18 | 36 | 15 |
| 84 | 126871* | Calbiochem | Merck Millipore | 60 | 18 | 50 | 11 | 73 | 0 |
| 85 | 128125, Aloisine A (RP107) | Calbiochem | Merck Millipore | –14 | 34 | 14 | 7 | 24 | 6 |
| 86 | 128135, Aloisine (RP106) | Calbiochem | Merck Millipore | –15 | 26 | –3 | 7 | 13 | 3 |
| 87 | 164640, Aminopurvalanol A | Calbiochem | Merck Millipore | –2 | 14 | –2 | 1 | 17 | 23 |
| 88 | 171260 | Calbiochem | Merck Millipore | –13 | 15 | 2 | 9 | 4 | 5 |
| 89 | 189404 | Calbiochem | Merck Millipore | –8 | 25 | 4 | 11 | 16 | 10 |
| 90 | 189405^#^ | Calbiochem | Merck Millipore | 17 | 14 | 10 | 3 | 13 | 12 |
| 91 | 189405^#^ | Calbiochem | Merck Millipore | –14 | 37 | 6 | 3 | 9 | 4 |
| 92 | 189406 | Calbiochem | Merck Millipore | 16 | 9 | 11 | 12 | 32 | 5 |
| 93 | 197221 | Calbiochem | Merck Millipore | 3 | 2 | 6 | 6 | 5 | 1 |
| 94 | 203297, Bisindolylmaleimide IV | Calbiochem | Merck Millipore | –3 | 9 | 11 | 6 | 14 | 3 |
| 95 | 203696, BPIQ-I | Calbiochem | Merck Millipore | 45 | 5 | 32 | 12 | 22 | 10 |
| 96 | 217695 | Calbiochem | Merck Millipore | 12 | 11 | 4 | 3 | 7 | 1 |
| 97 | 217714 | Calbiochem | Merck Millipore | –14 | 5 | 7 | 7 | 7 | 8 |
| 98 | 217720 | Calbiochem | Merck Millipore | 15 | 3 | 12 | 10 | 17 | 6 |
| 99 | 218696, D4476 | Calbiochem | Merck Millipore | –2 | 2 | 16 | 16 | 20 | 11 |
| 100 | 218710, TBCA | Calbiochem | Merck Millipore | 13 | 6 | 14 | 7 | 13 | 11 |
| 101 | 219476 | Calbiochem | Merck Millipore | 6 | 55 | 19 | 13 | 33 | 6 |
| 102 | 219477, NSC 625987 | Calbiochem | Merck Millipore | –8 | 5 | –4 | 11 | 1 | 26 |
| 103 | 219478* | Calbiochem | Merck Millipore | –4 | 6 | –10 | 9 | 66 | 12 |
| 104 | 219479, TG003 | Calbiochem | Merck Millipore | –2 | 4 | –5 | 6 | 9 | 13 |
| 105 | 219491* | Calbiochem | Merck Millipore | 75 | 6 | 30 | 12 | 67 | 2 |
| 106 | 220486 | Calbiochem | Merck Millipore | 10 | 4 | 3 | 5 | 6 | 6 |
| 107 | 234505* | Calbiochem | Merck Millipore | 62 | 4 | 29 | 4 | 29 | 8 |
| 108 | 238803 | Calbiochem | Merck Millipore | 6 | 6 | 13 | 10 | 23 | 10 |
| 109 | 238804, NU6140 | Calbiochem | Merck Millipore | 28 | 5 | 20 | 10 | 30 | 7 |
| 110 | 260962 | Calbiochem | Merck Millipore | –1 | 5 | 5 | 8 | 11 | 16 |
| 111 | 260964 | Calbiochem | Merck Millipore | –5 | 11 | 6 | 9 | 4 | 10 |
| 112 | 266788 | Calbiochem | Merck Millipore | –7 | 10 | 6 | 12 | 3 | 7 |
| 113 | 324673 | Calbiochem | Merck Millipore | 7 | 15 | 22 | 13 | 20 | 5 |
| 114 | 324674* | Calbiochem | Merck Millipore | 62 | 5 | 60 | 7 | 77 | 6 |
| 115 | 324840* | Calbiochem | Merck Millipore | 61 | 5 | 44 | 13 | 26 | 4 |
| 116 | 328009 | Calbiochem | Merck Millipore | 21 | 7 | 11 | 4 | 15 | 3 |
| 117 | 341251, Fascaplysin, synthetic* | Calbiochem | Merck Millipore | 103 | 3 | 107 | 4 | 101 | 0 |
| 118 | 343020 | Calbiochem | Merck Millipore | –9 | 9 | –8 | 11 | 3 | 9 |
| 119 | 343021 | Calbiochem | Merck Millipore | 10 | 16 | –13 | 11 | 4 | 14 |
| 120 | 343022 | Calbiochem | Merck Millipore | –22 | 31 | –18 | 9 | 7 | 13 |
| 121 | 344036 | Calbiochem | Merck Millipore | –27 | 28 | –5 | 8 | 1 | 8 |
| 122 | 361540 | Calbiochem | Merck Millipore | –15 | 37 | –5 | 12 | 9 | 7 |
| 123 | 361541 | Calbiochem | Merck Millipore | 14 | 10 | 7 | 2 | 11 | 6 |
| 124 | 361549 | Calbiochem | Merck Millipore | 34 | 2 | 24 | 10 | 22 | 5 |
| 125 | 361550 | Calbiochem | Merck Millipore | 35 | 6 | 6 | 12 | 35 | 12 |
| 126 | 361551 | Calbiochem | Merck Millipore | 12 | 20 | 3 | 7 | 18 | 10 |
| 127 | 361553 | Calbiochem | Merck Millipore | –10 | 7 | 0 | 11 | –4 | 13 |
| 128 | 361554, TWS119 | Calbiochem | Merck Millipore | –5 | 13 | 9 | 6 | 12 | 13 |
| 129 | 361555 | Calbiochem | Merck Millipore | 11 | 19 | 26 | 15 | 32 | 5 |
| 130 | 371806, GTP-14564 | Calbiochem | Merck Millipore | 10 | 5 | 11 | 5 | 10 | 1 |
| 131 | 371957, Isogranulatimide | Calbiochem | Merck Millipore | 10 | 2 | 12 | 8 | 14 | 5 |
| 132 | 375670, Herbimycin A, *Streptomyces* sp.* | Calbiochem | Merck Millipore | 55 | 9 | 44 | 14 | 77 | 2 |
| 133 | 400090, IC261* | Calbiochem | Merck Millipore | 8 | 45 | 41 | 9 | 70 | 6 |
| 134 | 402081, Indirubin derivative E804 | Calbiochem | Merck Millipore | 44 | 6 | 10 | 9 | 40 | 1 |
| 135 | 407248 | Calbiochem | Merck Millipore | –1 | 16 | –15 | 14 | –2 | 21 |
| 136 | 407601 | Calbiochem | Merck Millipore | –6 | 10 | –13 | 17 | 0 | 13 |
| 137 | 420099 | Calbiochem | Merck Millipore | –13 | 38 | 12 | 2 | 28 | 14 |
| 138 | 420104 | Calbiochem | Merck Millipore | 29 | 67 | 28 | 9 | 28 | 4 |
| 139 | 420121 | Calbiochem | Merck Millipore | –16 | 32 | –3 | 4 | 13 | 12 |
| 140 | 420123 | Calbiochem | Merck Millipore | 6 | 10 | 12 | 6 | 11 | 6 |
| 141 | 420126 | Calbiochem | Merck Millipore | 11 | 8 | 22 | 4 | 44 | 8 |
| 142 | 420129 | Calbiochem | Merck Millipore | 12 | 9 | 3 | 5 | 20 | 4 |
| 143 | 420135 | Calbiochem | Merck Millipore | –5 | 11 | –2 | 8 | –1 | 5 |
| 144 | 420136* | Calbiochem | Merck Millipore | 30 | 8 | 38 | 7 | 72 | 5 |
| 145 | 420298, K-252a, *Nocardiopsis* sp.* | Calbiochem | Merck Millipore | 77 | 6 | 29 | 11 | 69 | 7 |
| 146 | 422706, KN-62 | Calbiochem | Merck Millipore | 7 | 7 | 13 | 9 | 14 | 1 |
| 147 | 428205 | Calbiochem | Merck Millipore | –2 | 9 | 1 | 15 | 29 | 3 |
| 148 | 440203, LY 303511 | Calbiochem | Merck Millipore | 8 | 5 | 9 | 8 | 11 | 9 |
| 149 | 444937 | Calbiochem | Merck Millipore | 1 | 18 | 0 | 14 | 1 | 3 |
| 150 | 444938 | Calbiochem | Merck Millipore | 5 | 4 | –9 | 15 | 14 | 8 |
| 151 | 444939 | Calbiochem | Merck Millipore | 0 | 10 | 2 | 11 | 0 | 20 |
| 152 | 448101 | Calbiochem | Merck Millipore | –25 | 30 | –11 | 14 | 6 | 9 |
| 153 | 454861 | Calbiochem | Merck Millipore | –16 | 19 | –7 | 11 | 15 | 16 |
| 154 | 475863* | Calbiochem | Merck Millipore | –8 | 23 | 0 | 7 | 55 | 21 |
| 155 | 481406 | Calbiochem | Merck Millipore | –3 | 47 | 31 | 12 | 22 | 8 |
| 156 | 506121 | Calbiochem | Merck Millipore | 6 | 4 | 13 | 11 | 11 | 4 |
| 157 | 506126 | Calbiochem | Merck Millipore | 12 | 5 | –19 | 11 | 0 | 1 |
| 158 | 513030, PD 169316 | Calbiochem | Merck Millipore | –19 | 9 | –12 | 9 | 1 | 7 |
| 159 | 513035, PD 158780* | Calbiochem | Merck Millipore | 56 | 2 | 9 | 21 | 13 | 4 |
| 160 | 513040, PD 174265* | Calbiochem | Merck Millipore | 55 | 6 | 30 | 16 | 18 | 2 |
| 161 | 521231 | Calbiochem | Merck Millipore | –16 | 18 | –4 | 5 | 15 | 12 |
| 162 | 521232 | Calbiochem | Merck Millipore | 30 | 4 | 19 | 6 | 29 | 2 |
| 163 | 521233* | Calbiochem | Merck Millipore | 40 | 3 | 51 | 11 | 84 | 2 |
| 164 | 521234 | Calbiochem | Merck Millipore | 24 | 9 | 21 | 7 | 23 | 2 |
| 165 | 521275* | Calbiochem | Merck Millipore | 105 | 4 | 105 | 4 | 100 | 0 |
| 166 | 527455 | Calbiochem | Merck Millipore | –8 | 25 | –8 | 16 | 1 | 12 |
| 167 | 528106 | Calbiochem | Merck Millipore | 1 | 17 | –11 | 10 | 1 | 14 |
| 168 | 528108 | Calbiochem | Merck Millipore | –14 | 37 | –8 | 19 | –2 | 16 |
| 169 | 529574, PP3 | Calbiochem | Merck Millipore | –30 | 27 | –6 | 13 | 1 | 17 |
| 170 | 529581, PP1 analogue II, 1NM-PP1 | Calbiochem | Merck Millipore | 5 | 3 | 5 | 5 | 11 | 5 |
| 171 | 539648, Staurosporine, N-benzoyl-* | Calbiochem | Merck Millipore | 81 | 8 | 44 | 5 | 95 | 0 |
| 172 | 539652 | Calbiochem | Merck Millipore | 7 | 8 | 14 | 10 | 13 | 9 |
| 173 | 539654 | Calbiochem | Merck Millipore | 0 | 6 | –18 | 7 | 10 | 2 |
| 174 | 540500, Purvalanol A | Calbiochem | Merck Millipore | –9 | 5 | 3 | 12 | 14 | 12 |
| 175 | 553210, Rapamycin | Calbiochem | Merck Millipore | 39 | 0 | 6 | 8 | 44 | 2 |
| 176 | 555553 | Calbiochem | Merck Millipore | –9 | 3 | 6 | 5 | 6 | 7 |
| 177 | 555554 | Calbiochem | Merck Millipore | 4 | 7 | 17 | 6 | 23 | 1 |
| 178 | 559387, SB-202747 | Calbiochem | Merck Millipore | –4 | 6 | 1 | 4 | 0 | 2 |
| 179 | 559389, SB-203580 | Calbiochem | Merck Millipore | 3 | 1 | 4 | 11 | 16 | 0 |
| 180 | 559396, SB-220025 | Calbiochem | Merck Millipore | 15 | 7 | 4 | 6 | 31 | 2 |
| 181 | 559402, SB-218078 | Calbiochem | Merck Millipore | 21 | 16 | 5 | 18 | 49 | 1 |
| 182 | 565625, SC-68376 | Calbiochem | Merck Millipore | –17 | 35 | –13 | 13 | –7 | 6 |
| 183 | 567305 SKF-86002 | Calbiochem | Merck Millipore | –28 | 26 | –14 | 12 | –5 | 17 |
| 184 | 567731 | Calbiochem | Merck Millipore | –8 | 8 | –10 | 11 | 1 | 13 |
| 185 | 569397, Staurosporine, *Streptomyces* sp.^#^* | Calbiochem | Merck Millipore | 94 | 14 | 88 | 7 | 100 | 0 |
| 186 | 569397, Staurosporine, *Streptomyces* sp.^#^ | Calbiochem | Merck Millipore | 102 | 5 | 75 | 12 | 100 | 0 |
| 187 | 570250, STO-609 | Calbiochem | Merck Millipore | 21 | 8 | 4 | 2 | 18 | 7 |
| 188 | 572635, SU6656 | Calbiochem | Merck Millipore | 3 | 3 | 18 | 16 | 8 | 7 |
| 189 | 572650, SU9516 | Calbiochem | Merck Millipore | –17 | 2 | –6 | 4 | 7 | 7 |
| 190 | 572660 SU11652 | Calbiochem | Merck Millipore | –18 | 7 | 8 | 17 | 41 | 4 |
| 191 | 574711 | Calbiochem | Merck Millipore | –5 | 2 | 4 | 13 | 10 | 8 |
| 192 | 574712 | Calbiochem | Merck Millipore | –4 | 2 | 5 | 1 | 10 | 7 |
| 193 | 574713 | Calbiochem | Merck Millipore | –7 | 9 | 6 | 4 | –2 | 5 |
| 194 | 616373 | Calbiochem | Merck Millipore | 14 | 14 | 28 | 10 | 15 | 4 |
| 195 | 616451 | Calbiochem | Merck Millipore | –2 | 2 | 21 | 15 | 20 | 1 |
| 196 | 616453 | Calbiochem | Merck Millipore | 18 | 10 | 37 | 10 | 25 | 5 |
| 197 | 658440, AG 112 | Calbiochem | Merck Millipore | 2 | 10 | –14 | 7 | –7 | 8 |
| 198 | 676480 | Calbiochem | Merck Millipore | –25 | 28 | –12 | 12 | –8 | 3 |
| 199 | 676481 | Calbiochem | Merck Millipore | –16 | 5 | –14 | 14 | –5 | 10 |
| 200 | 676485 | Calbiochem | Merck Millipore | –6 | 8 | –9 | 17 | –4 | 12 |
| 201 | 676487 | Calbiochem | Merck Millipore | –19 | 34 | 3 | 12 | –3 | 10 |
| 202 | 220285, Chelerythrine chloride | Calbiochem | Merck Millipore | 3 | 8 | 1 | 11 | 13 | 2 |
| 203 | S1207, AV-951(Tivozanib) |  | SelleckChem | 17 | 12 | –4 | 3 | 30 | 2 |
| 204 | GW632580X | PKIS | GSK | 21 | 19 | 6 | 7 | –1 | 6 |
| 205 | GW620972X | PKIS | GSK | 20 | 21 | 6 | 14 | 9 | 8 |
| 206 | SB-220025-R* | PKIS | GSK | 12 | 8 | –26 | 8 | 33 | 11 |
| 207 | SB-220025-A | PKIS | GSK | 5 | 5 | –30 | 26 | 7 | 6 |
| 208 | SKF-86002-A2 | PKIS | GSK | 5 | 4 | –3 | 15 | 3 | 13 |
| 209 | GW282974X | PKIS | GSK | 13 | 10 | 34 | 39 | 3 | 21 |
| 210 | GW769076X | PKIS | GSK | 3 | 5 | –3 | 9 | 1 | 12 |
| 211 | GW775608X | PKIS | GSK | 6 | 8 | –11 | 3 | 8 | 8 |
| 212 | GW549390X | PKIS | GSK | 11 | 17 | –14 | 52 | 9 | 20 |
| 213 | GW572399X | PKIS | GSK | 5 | 10 | –4 | 9 | –16 | 27 |
| 214 | GW572401X | PKIS | GSK | –3 | 12 | –21 | 12 | –8 | 29 |
| 215 | GW575533A | PKIS | GSK | –1 | 6 | 25 | 26 | –6 | 21 |
| 216 | GW577921A | PKIS | GSK | 11 | 8 | 0 | 9 | 0 | 8 |
| 217 | GW580509X | PKIS | GSK | 13 | 7 | –12 | 3 | 2 | 10 |
| 218 | GW621431X | PKIS | GSK | 1 | 3 | 4 | 17 | 17 | 17 |
| 219 | GW621970X | PKIS | GSK | 4 | 14 | 1 | 2 | –6 | 10 |
| 220 | GW622055X | PKIS | GSK | 5 | 8 | –7 | 16 | –1 | 12 |
| 221 | GW627512B | PKIS | GSK | 4 | 6 | 11 | 8 | 7 | 12 |
| 222 | GW627834A | PKIS | GSK | 17 | 31 | 6 | 3 | 3 | 7 |
| 223 | GW631581B | PKIS | GSK | 0 | 4 | –4 | 13 | –6 | 15 |
| 224 | GW632046X | PKIS | GSK | 10 | 13 | –2 | 3 | 11 | 15 |
| 225 | GW641155A | PKIS | GSK | 5 | 3 | 22 | 15 | –6 | 10 |
| 226 | GW678313X | PKIS | GSK | 11 | 10 | 0 | 10 | 11 | 9 |
| 227 | GSK718429A | PKIS | GSK | 2 | 7 | 3 | 8 | –1 | 9 |
| 228 | GW572738X | PKIS | GSK | 3 | 4 | 4 | 11 | –2 | 10 |
| 229 | GW846105X | PKIS | GSK | 13 | 9 | –3 | 21 | –10 | 18 |
| 230 | SB-347804 | PKIS | GSK | 9 | 4 | 0 | 10 | 17 | 24 |
| 231 | SB-657836-AAA | PKIS | GSK | 5 | 2 | –2 | 5 | 1 | 7 |
| 232 | SB-814597 | PKIS | GSK | 14 | 17 | –2 | 44 | 18 | 36 |
| 233 | GSK586581A | PKIS | GSK | 5 | 7 | 12 | 13 | 12 | 18 |
| 234 | GSK605714A | PKIS | GSK | –5 | 6 | –24 | 12 | –3 | 16 |
| 235 | GSK620503A | PKIS | GSK | 3 | 4 | 13 | 20 | 7 | 12 |
| 236 | GSK625137A | PKIS | GSK | 16 | 12 | –4 | 13 | –1 | 3 |
| 237 | GSK635416A | PKIS | GSK | 2 | 1 | 5 | 22 | 11 | 13 |
| 238 | GSK711701A | PKIS | GSK | 2 | 2 | 2 | 9 | 3 | 9 |
| 239 | GW549034X | PKIS | GSK | 5 | 2 | –2 | 6 | 12 | 12 |
| 240 | GW785404X | PKIS | GSK | –3 | 7 | –7 | 8 | 0 | 15 |
| 241 | SB-734117 | PKIS | GSK | 11 | 7 | 10 | 10 | –15 | 36 |
| 242 | SB-736290 | PKIS | GSK | 11 | 10 | 2 | 3 | 13 | 8 |
| 243 | SB-736302 | PKIS | GSK | 6 | 6 | 7 | 14 | 13 | 24 |
| 244 | SB-738561 | PKIS | GSK | 6 | 9 | 21 | 36 | 4 | 12 |
| 245 | SB-737198* | PKIS | GSK | 6 | 10 | 36 | 23 | 5 | 9 |
| 246 | SB-744941 | PKIS | GSK | 4 | 0 | –1 | 11 | –11 | 22 |
| 247 | SB-750140* | PKIS | GSK | 13 | 8 | 9 | 5 | 40 | 5 |
| 248 | SB-751148 | PKIS | GSK | 11 | 13 | 3 | 6 | 5 | 9 |
| 249 | SB-751399 | PKIS | GSK | 3 | 9 | 22 | 26 | 1 | 13 |
| 250 | SB-333612 | PKIS | GSK | 6 | 6 | –12 | 2 | 0 | 4 |
| 251 | SB-358518 | PKIS | GSK | 8 | 5 | –6 | 8 | 3 | 8 |
| 252 | SB-360741 | PKIS | GSK | 4 | 4 | –6 | 11 | –3 | 18 |
| 253 | SB-361058 | PKIS | GSK | 4 | 7 | –5 | 5 | 7 | 9 |
| 254 | SB-376719 | PKIS | GSK | 2 | 7 | 12 | 15 | 0 | 14 |
| 255 | SB-390523 | PKIS | GSK | 3 | 6 | –16 | 13 | 0 | 9 |
| 256 | SB-390527 | PKIS | GSK | 3 | 4 | 1 | 8 | 9 | 2 |
| 257 | SB-409513 | PKIS | GSK | 14 | 8 | –17 | 11 | 0 | 9 |
| 258 | SB-409514 | PKIS | GSK | 3 | 3 | 1 | 13 | –3 | 8 |
| 259 | SKF-62604 | PKIS | GSK | 11 | 10 | 4 | 28 | –3 | 14 |
| 260 | GW819230X | PKIS | GSK | 1 | 18 | –17 | 30 | 19 | 36 |
| 261 | GSK319347A | PKIS | GSK | 7 | 14 | 2 | 31 | 3 | 34 |
| 262 | SB-725317* | PKIS | GSK | –6 | 10 | 45 | 5 | 1 | 13 |
| 263 | SB-732941 | PKIS | GSK | 1 | 1 | 7 | 6 | 6 | 19 |
| 264 | SB-735465 | PKIS | GSK | 6 | 7 | 3 | 9 | 8 | 19 |
| 265 | SB-735467 | PKIS | GSK | 3 | 7 | 2 | 5 | 2 | 16 |
| 266 | SB-738482 | PKIS | GSK | 21 | 20 | –2 | 3 | 10 | 13 |
| 267 | SB-739452 | PKIS | GSK | 10 | 8 | –1 | 8 | 3 | 8 |
| 268 | SB-741905 | PKIS | GSK | 5 | 7 | –5 | 20 | –2 | 11 |
| 269 | SB-742864 | PKIS | GSK | 6 | 5 | 21 | 15 | 20 | 20 |
| 270 | SB-742865 | PKIS | GSK | 9 | 6 | –3 | 8 | 12 | 5 |
| 271 | GW651576X | PKIS | GSK | 2 | 4 | 4 | 6 | –12 | 27 |
| 272 | GW659893X | PKIS | GSK | 10 | 16 | –9 | 13 | –7 | 8 |
| 273 | GW703087X | PKIS | GSK | 24 | 30 | 7 | 4 | 12 | 19 |
| 274 | GW772405X | PKIS | GSK | 12 | 9 | –4 | 6 | 7 | 16 |
| 275 | GW794726X | PKIS | GSK | 1 | 8 | 4 | 7 | 12 | 7 |
| 276 | GW799251X | PKIS | GSK | 15 | 28 | –15 | 20 | 0 | 4 |
| 277 | GW807930X | PKIS | GSK | 11 | 10 | 1 | 7 | 8 | 10 |
| 278 | GSK1000163A | PKIS | GSK | 9 | 14 | 13 | 17 | 6 | 9 |
| 279 | GSK1007102B* | PKIS | GSK | 43 | 25 | 57 | 54 | 74 | 18 |
| 280 | GSK938890A | PKIS | GSK | 10 | 8 | –11 | 3 | 5 | 8 |
| 281 | GSK943949A | PKIS | GSK | 10 | 3 | 1 | 5 | 15 | 18 |
| 282 | GSK949675A | PKIS | GSK | 19 | 12 | 0 | 44 | 6 | 21 |
| 283 | GW824645A | PKIS | GSK | 10 | 8 | –7 | 13 | –1 | 11 |
| 284 | GW831090X | PKIS | GSK | 9 | 19 | 1 | 12 | –13 | 25 |
| 285 | GW831091X | PKIS | GSK | 7 | 1 | –17 | 9 | 1 | 13 |
| 286 | GW406731X | PKIS | GSK | 11 | 9 | 2 | 20 | –4 | 7 |
| 287 | GW427984X | PKIS | GSK | –2 | 5 | –3 | 14 | –4 | 27 |
| 288 | GW432441X | PKIS | GSK | 13 | 17 | 5 | 14 | 4 | 26 |
| 289 | GW435821X | PKIS | GSK | 7 | 4 | –17 | 3 | –6 | 13 |
| 290 | GW439255X | PKIS | GSK | 9 | 16 | 0 | 12 | –11 | 13 |
| 291 | GW441806A | PKIS | GSK | 6 | 7 | –3 | 6 | 14 | 21 |
| 292 | GW445012X | PKIS | GSK | –6 | 6 | –11 | 25 | 0 | 29 |
| 293 | GW445014X | PKIS | GSK | 6 | 8 | –8 | 14 | –5 | 12 |
| 294 | GW445015X | PKIS | GSK | 9 | 5 | –10 | 6 | –5 | 4 |
| 295 | GW445017X | PKIS | GSK | –2 | 6 | –5 | 14 | –6 | 28 |
| 296 | GW450241X | PKIS | GSK | 8 | 4 | –3 | 10 | –6 | 13 |
| 297 | GW458344A | PKIS | GSK | 2 | 6 | –1 | 4 | 5 | 7 |
| 298 | GW459057A | PKIS | GSK | 8 | 4 | –4 | 8 | 4 | 13 |
| 299 | GW743024X | PKIS | GSK | 7 | 13 | 0 | 9 | 4 | 7 |
| 300 | GW782907X | PKIS | GSK | 3 | 4 | –6 | 10 | 5 | 14 |
| 301 | GW782912X | PKIS | GSK | 0 | 7 | 1 | 11 | 3 | 11 |
| 302 | GW785974X | PKIS | GSK | 6 | 6 | –2 | 23 | 15 | 19 |
| 303 | GW796920X | PKIS | GSK | –2 | 3 | –3 | 8 | 12 | 6 |
| 304 | GW796921X | PKIS | GSK | –7 | 7 | –5 | 7 | –9 | 30 |
| 305 | GW806776X | PKIS | GSK | 2 | 6 | 25 | 15 | 4 | 10 |
| 306 | GW701032X | PKIS | GSK | 8 | 19 | 7 | 24 | –11 | 42 |
| 307 | GW708893X | PKIS | GSK | 16 | 6 | –10 | 15 | 13 | 11 |
| 308 | GW734508X | PKIS | GSK | 1 | 2 | –4 | 7 | 5 | 12 |
| 309 | GW607117X | PKIS | GSK | 11 | 7 | –13 | 5 | 10 | 8 |
| 310 | GW856804X | PKIS | GSK | 11 | 7 | –4 | 4 | –1 | 7 |
| 311 | GSK237700A | PKIS | GSK | 16 | 8 | –11 | 30 | 3 | 7 |
| 312 | GSK237701A* | PKIS | GSK | 40 | 19 | –4 | 12 | 54 | 33 |
| 313 | GSK317314A* | PKIS | GSK | 11 | 12 | 5 | 14 | 57 | 5 |
| 314 | GSK317315A | PKIS | GSK | 6 | 2 | 11 | 4 | 20 | 7 |
| 315 | GSK326090A* | PKIS | GSK | 20 | 5 | 24 | 5 | 38 | 42 |
| 316 | GSK571989A* | PKIS | GSK | 19 | 6 | 0 | 16 | 72 | 7 |
| 317 | GSK579289A* | PKIS | GSK | 32 | 16 | 4 | 7 | 52 | 32 |
| 318 | GSK978744A* | PKIS | GSK | 8 | 6 | 27 | 29 | 50 | 7 |
| 319 | GW843682X | PKIS | GSK | 3 | 6 | 4 | 6 | 0 | 9 |
| 320 | GW852849X | PKIS | GSK | 8 | 16 | –2 | 5 | –9 | 25 |
| 321 | GSK180736A | PKIS | GSK | –2 | 10 | –7 | 6 | 11 | 15 |
| 322 | GSK270822A | PKIS | GSK | –1 | 11 | 0 | 6 | 22 | 17 |
| 323 | GSK299115A | PKIS | GSK | –1 | 9 | 6 | 39 | 6 | 11 |
| 324 | GSK466314A | PKIS | GSK | 2 | 8 | 16 | 19 | 21 | 14 |
| 325 | GSK466317A | PKIS | GSK | 4 | 4 | 11 | 21 | 13 | 9 |
| 326 | GW461104A* | PKIS | GSK | 29 | 22 | 30 | 23 | –4 | 8 |
| 327 | GW569530A | PKIS | GSK | 7 | 8 | 19 | 27 | –9 | 11 |
| 328 | GR105659X | PKIS | GSK | 8 | 5 | 7 | 8 | 14 | 9 |
| 329 | GW275616X | PKIS | GSK | 3 | 11 | 3 | 13 | 11 | 11 |
| 330 | GW278681X | PKIS | GSK | 6 | 5 | 3 | 8 | 10 | 23 |
| 331 | GW301789X | PKIS | GSK | 7 | 13 | –8 | 10 | 1 | 7 |
| 332 | GW442130X | PKIS | GSK | 8 | 4 | –8 | 13 | 5 | 26 |
| 333 | GW679410X | PKIS | GSK | 6 | 9 | –3 | 23 | 15 | 9 |
| 334 | GW680975X | PKIS | GSK | 0 | 8 | 28 | 27 | 11 | 14 |
| 335 | GW682841X | PKIS | GSK | –5 | 8 | –2 | 10 | 5 | 18 |
| 336 | GW695874X | PKIS | GSK | 0 | 6 | 15 | 15 | 0 | 30 |
| 337 | GW711782X | PKIS | GSK | 1 | 3 | 11 | 15 | 7 | 31 |
| 338 | GW410563A* | PKIS | GSK | 12 | 14 | 35 | 5 | 4 | 5 |
| 339 | GW612286X* | PKIS | GSK | 10 | 8 | 37 | 10 | 13 | 4 |
| 340 | GW654652C | PKIS | GSK | 7 | 10 | 9 | 29 | 2 | 16 |
| 341 | GW770220A | PKIS | GSK | 13 | 8 | –16 | 4 | 0 | 3 |
| 342 | GW771127A | PKIS | GSK | 4 | 13 | 4 | 0 | 12 | 21 |
| 343 | GSK953913A | PKIS | GSK | 3 | 12 | 11 | 20 | 3 | 12 |
| 344 | GSK980961A | PKIS | GSK | 15 | 15 | –1 | 18 | 1 | 2 |
| 345 | GW806742X | PKIS | GSK | 9 | 7 | –6 | 9 | 5 | 18 |
| 346 | GW809897X | PKIS | GSK | 7 | 4 | 1 | 8 | 9 | 6 |
| 347 | GW830263A | PKIS | GSK | 8 | 6 | –9 | 3 | 5 | 14 |
| 348 | GW830365A | PKIS | GSK | 5 | 3 | 1 | 5 | 0 | 7 |
| 349 | GW830900A | PKIS | GSK | 9 | 13 | –1 | 14 | 5 | 6 |
| 350 | GSK248233A | PKIS | GSK | 8 | 16 | 2 | 19 | 12 | 15 |
| 351 | GSK269962B* | PKIS | GSK | 14 | 9 | 17 | 2 | 34 | 12 |
| 352 | GW589961A | PKIS | GSK | 9 | 11 | –6 | 21 | –19 | 28 |
| 353 | GW607049C | PKIS | GSK | –5 | 7 | –39 | 22 | –3 | 7 |
| 354 | GW659386A | PKIS | GSK | 5 | 10 | –30 | 8 | –2 | 12 |
| 355 | GW673715X | PKIS | GSK | 2 | 3 | –1 | 11 | –5 | 27 |
| 356 | GW680908A | PKIS | GSK | 7 | 8 | –5 | 9 | 0 | 12 |
| 357 | GW683134A | PKIS | GSK | 21 | 21 | –7 | 18 | –5 | 8 |
| 358 | GW693917A | PKIS | GSK | 5 | 17 | –17 | 8 | 2 | 10 |
| 359 | GW694234A | PKIS | GSK | 3 | 2 | 2 | 19 | –1 | 5 |
| 360 | GW694590A | PKIS | GSK | 1 | 4 | –15 | 7 | –9 | 15 |
| 361 | GW700494A | PKIS | GSK | 2 | 3 | 6 | 13 | 11 | 11 |
| 362 | GW701427A | PKIS | GSK | 0 | 3 | –13 | 6 | 1 | 10 |
| 363 | GW709042A | PKIS | GSK | 7 | 6 | –26 | 10 | 7 | 11 |
| 364 | GSK1023156A | PKIS | GSK | –1 | 7 | –6 | 7 | 3 | 7 |
| 365 | GSK1030058A | PKIS | GSK | 7 | 5 | –9 | 11 | 8 | 8 |
| 366 | GSK1030059A | PKIS | GSK | 9 | 11 | 11 | 5 | –8 | 19 |
| 367 | GSK1030061A | PKIS | GSK | 8 | 6 | –5 | 8 | 9 | 17 |
| 368 | GSK1030062A | PKIS | GSK | 7 | 8 | 4 | 7 | –8 | 13 |
| 369 | GSK204925A | PKIS | GSK | 11 | 8 | –3 | 15 | 10 | 14 |
| 370 | GSK312948A | PKIS | GSK | 8 | 6 | –15 | 20 | –7 | 13 |
| 371 | GW804482X | PKIS | GSK | 9 | 9 | –3 | 7 | –6 | 20 |
| 372 | GW853606X | PKIS | GSK | –5 | 8 | –15 | 17 | 0 | 31 |
| 373 | GW853609X | PKIS | GSK | 6 | 9 | 0 | 6 | 2 | 23 |
| 374 | GW693481X | PKIS | GSK | 7 | 7 | 5 | 15 | 13 | 6 |
| 375 | GW780159X | PKIS | GSK | 9 | 11 | 2 | 11 | –7 | 19 |
| 376 | GW785804X | PKIS | GSK | 2 | 7 | 4 | 2 | 16 | 8 |
| 377 | GW786460X | PKIS | GSK | 6 | 6 | 19 | 13 | 13 | 6 |
| 378 | GSK554170A | PKIS | GSK | 6 | 5 | 7 | 19 | 13 | 13 |
| 379 | GSK561866B | PKIS | GSK | 10 | 5 | 1 | 11 | 8 | 8 |
| 380 | GSK614526A* | PKIS | GSK | 10 | 4 | 0 | 12 | 35 | 15 |
| 381 | GSK619487A | PKIS | GSK | 3 | 11 | 26 | 33 | 18 | 24 |
| 382 | GW876790X | PKIS | GSK | 2 | 9 | 19 | 9 | 26 | 9 |
| 383 | SB-759335-B | PKIS | GSK | 4 | 7 | –7 | 11 | 0 | 11 |
| 384 | SB-400868-A | PKIS | GSK | 15 | 11 | 16 | 12 | –5 | 11 |
| 385 | SB-431533 | PKIS | GSK | 12 | 12 | –7 | 22 | 3 | 24 |
| 386 | SB-431542-A | PKIS | GSK | –4 | 17 | 7 | 4 | 4 | 25 |
| 387 | SKF-86055 | PKIS | GSK | 12 | 20 | –9 | 16 | –2 | 21 |
| 388 | GR269666A | PKIS | GSK | 1 | 9 | –22 | 20 | –2 | 22 |
| 389 | GW282449A* | PKIS | GSK | 37 | 16 | 53 | 9 | 3 | 10 |
| 390 | GW301888X | PKIS | GSK | 18 | 24 | 7 | 6 | 2 | 11 |
| 391 | GW820759X | PKIS | GSK | 7 | 5 | 1 | 17 | –16 | 43 |
| 392 | GW575808A | PKIS | GSK | 9 | 11 | 3 | 7 | 3 | 14 |
| 393 | GW759710A | PKIS | GSK | 15 | 21 | 4 | 5 | –1 | 18 |
| 394 | GW782612X | PKIS | GSK | 20 | 23 | 8 | 30 | 0 | 18 |
| 395 | GW642125X | PKIS | GSK | 8 | 7 | 4 | 15 | 19 | 17 |
| 396 | GW642138X | PKIS | GSK | 14 | 7 | –6 | 17 | 19 | 25 |
| 397 | GW578748X | PKIS | GSK | 19 | 24 | –5 | 11 | 8 | 9 |
| 398 | GW644007X | PKIS | GSK | 1 | 6 | 4 | 9 | 2 | 9 |
| 399 | GW784307A | PKIS | GSK | –5 | 5 | 3 | 12 | 15 | 9 |
| 400 | GW794607X | PKIS | GSK | –3 | 14 | –8 | 13 | –10 | 31 |
| 401 | GW809885X | PKIS | GSK | 12 | 9 | –6 | 8 | 0 | 6 |
| 402 | GW811168X | PKIS | GSK | –9 | 13 | 4 | 5 | –8 | 14 |
| 403 | GW817394X | PKIS | GSK | 17 | 21 | 5 | 8 | 3 | 11 |
| 404 | GW817396X | PKIS | GSK | 6 | 4 | 14 | 26 | –8 | 23 |
| 405 | GW829874X | PKIS | GSK | 1 | 8 | –2 | 8 | 10 | 7 |
| 406 | GW829877X | PKIS | GSK | 6 | 4 | 2 | 15 | 5 | 4 |
| 407 | SB-772077-B | PKIS | GSK | 5 | 1 | 12 | 13 | 21 | 18 |
| 408 | GW284372X | PKIS | GSK | 18 | 13 | 33 | 38 | 4 | 5 |
| 409 | GW458787A | PKIS | GSK | –4 | 12 | 9 | 11 | –3 | 36 |
| 410 | GW567808A | PKIS | GSK | 15 | 12 | 1 | 15 | 0 | 6 |
| 411 | GW568377A | PKIS | GSK | 3 | 4 | –5 | 4 | 6 | 4 |
| 412 | GW574782A | PKIS | GSK | –8 | 16 | –11 | 12 | –7 | 31 |
| 413 | GW574783B | PKIS | GSK | 11 | 5 | 7 | 11 | 1 | 6 |
| 414 | GW576484X | PKIS | GSK | 9 | 6 | –4 | 23 | –17 | 25 |
| 415 | GW576609A* | PKIS | GSK | 46 | 13 | 47 | 7 | 6 | 9 |
| 416 | GW576924A | PKIS | GSK | –8 | 9 | 29 | 9 | –10 | 20 |
| 417 | GW580496A | PKIS | GSK | 0 | 1 | –3 | 7 | –5 | 14 |
| 418 | GW583373A* | PKIS | GSK | 14 | 23 | 39 | 4 | 3 | 22 |
| 419 | GW615311X | PKIS | GSK | 3 | 6 | 26 | 13 | 5 | 16 |
| 420 | GW616030X* | PKIS | GSK | 30 | 14 | 38 | 22 | 6 | 12 |
| 421 | GW621823A | PKIS | GSK | 11 | 10 | 25 | 0 | –6 | 6 |
| 422 | GW633459A | PKIS | GSK | 9 | 14 | 19 | 16 | 8 | 6 |
| 423 | GSK2220400A* | PKIS | GSK | 11 | 8 | 52 | 27 | 8 | 12 |
| 424 | SB-437013 | PKIS | GSK | –4 | 2 | 8 | 8 | 10 | 9 |
| 425 | SB-630812 | PKIS | GSK | 0 | 5 | –3 | 11 | 19 | 16 |
| 426 | SB-633825 | PKIS | GSK | 14 | 11 | 4 | 10 | –9 | 18 |
| 427 | GW768505A | PKIS | GSK | 5 | 18 | –1 | 15 | –2 | 29 |
| 428 | GW770249A | PKIS | GSK | 10 | 6 | 7 | 3 | 11 | 3 |
| 429 | GW770249X | PKIS | GSK | 9 | 8 | 0 | 8 | –5 | 12 |
| 430 | GW795486X | PKIS | GSK | 6 | 4 | –8 | 10 | –7 | 14 |
| 431 | GW795493X | PKIS | GSK | 16 | 12 | –4 | 10 | 11 | 9 |
| 432 | GW275944X | PKIS | GSK | 2 | 8 | 10 | 22 | –8 | 19 |
| 433 | GW276655X | PKIS | GSK | 2 | 2 | 2 | 8 | –3 | 20 |
| 434 | GW280670X | PKIS | GSK | 20 | 27 | –9 | 3 | –1 | 7 |
| 435 | GW282536X | PKIS | GSK | 1 | 12 | –14 | 14 | –18 | 17 |
| 436 | GW290597X | PKIS | GSK | 6 | 6 | –6 | 9 | 6 | 17 |
| 437 | GW297361X | PKIS | GSK | –5 | 4 | –5 | 7 | –4 | 9 |
| 438 | GW300653X | PKIS | GSK | 3 | 2 | –6 | 14 | –3 | 4 |
| 439 | GW300657X | PKIS | GSK | 2 | 5 | 2 | 7 | –4 | 7 |
| 440 | GW300660X | PKIS | GSK | 2 | 4 | –4 | 15 | 6 | 11 |
| 441 | GW301784X | PKIS | GSK | 18 | 17 | 13 | 11 | –11 | 4 |
| 442 | GW305178X | PKIS | GSK | 2 | 3 | –5 | 11 | 2 | 21 |
| 443 | GW335962X | PKIS | GSK | 7 | 7 | –2 | 4 | –2 | 9 |
| 444 | GW352430A | PKIS | GSK | 5 | 7 | –3 | 9 | –5 | 2 |
| 445 | GW396574X | PKIS | GSK | 1 | 12 | 9 | 42 | –18 | 10 |
| 446 | GW416469X | PKIS | GSK | 6 | 6 | 10 | 15 | –13 | 21 |
| 447 | GW416981X | PKIS | GSK | 10 | 11 | 19 | 7 | 2 | 8 |
| 448 | SB-239272 | PKIS | GSK | 5 | 7 | –8 | 32 | 1 | 37 |
| 449 | SB-242717 | PKIS | GSK | 1 | 3 | –15 | 10 | 2 | 19 |
| 450 | SB-242718 | PKIS | GSK | 0 | 2 | –11 | 13 | –10 | 17 |
| 451 | SB-242719 | PKIS | GSK | –2 | 4 | 3 | 5 | 5 | 14 |
| 452 | SB-242721 | PKIS | GSK | 4 | 18 | 3 | 32 | 10 | 13 |
| 453 | SB-245392 | PKIS | GSK | 2 | 2 | –4 | 7 | 12 | 12 |
| 454 | SB-250715 | PKIS | GSK | 5 | 8 | 18 | 7 | –1 | 20 |
| 455 | SB-251505 | PKIS | GSK | 10 | 9 | 9 | 37 | –1 | 29 |
| 456 | SB-251527 | PKIS | GSK | –5 | 11 | –15 | 1 | 5 | 24 |
| 457 | SB-253226 | PKIS | GSK | 4 | 5 | –7 | 16 | 3 | 10 |
| 458 | SB-253228 | PKIS | GSK | –4 | 11 | 7 | 4 | 3 | 13 |
| 459 | SB-254169 | PKIS | GSK | –5 | 9 | –14 | 11 | –5 | 13 |
| 460 | SB-264865 | PKIS | GSK | 5 | 5 | –2 | 24 | 9 | 8 |
| 461 | SB-264866 | PKIS | GSK | 6 | 5 | 5 | 3 | 4 | 16 |
| 462 | SB-278538 | PKIS | GSK | 6 | 8 | 5 | 12 | 3 | 20 |
| 463 | SB-278539 | PKIS | GSK | 3 | 6 | 2 | 13 | 8 | 12 |
| 464 | SB-284847-BT | PKIS | GSK | 8 | 15 | –4 | 11 | 7 | 7 |
| 465 | SB-285234-W | PKIS | GSK | 19 | 17 | –1 | 12 | 17 | 23 |
| 466 | GSK317354A | PKIS | GSK | 2 | 7 | 0 | 19 | 3 | 14 |
| 467 | SB-210313 | PKIS | GSK | 3 | 5 | –7 | 3 | 12 | 13 |
| 468 | SB-216385 | PKIS | GSK | 1 | 1 | –9 | 7 | –8 | 17 |
| 469 | SB-220455 | PKIS | GSK | 1 | 2 | –2 | 14 | 9 | 20 |
| 470 | SB-221466 | PKIS | GSK | 4 | 11 | –16 | 5 | 8 | 6 |
| 471 | SB-223133 | PKIS | GSK | 2 | 0 | –4 | 14 | 15 | 12 |
| 472 | SB-226879 | PKIS | GSK | 2 | 4 | 1 | 8 | 16 | 10 |
| 473 | SB-236687 | PKIS | GSK | 6 | 3 | 3 | 11 | 17 | 13 |
| 474 | GSK200398A | PKIS | GSK | –7 | 9 | 6 | 0 | 1 | 16 |
| 475 | GSK238583A | PKIS | GSK | 11 | 9 | –6 | 17 | 18 | 12 |
| 476 | GSK259178A | PKIS | GSK | 16 | 19 | 24 | 38 | 2 | 9 |
| 477 | GW869810X | PKIS | GSK | 11 | 9 | –15 | 3 | –9 | 14 |
| 478 | GW784752X | PKIS | GSK | –7 | 7 | 8 | 2 | –1 | 12 |
| 479 | GW813360X | PKIS | GSK | 3 | 5 | 1 | 29 | 5 | 14 |
| 480 | GI261520A | PKIS | GSK | –1 | 6 | 7 | 16 | 10 | 17 |
| 481 | GW680191X* | PKIS | GSK | 57 | 9 | 39 | 31 | 6 | 3 |
| 482 | GW440139A* | PKIS | GSK | 6 | 5 | 37 | 7 | 16 | 28 |
| 483 | GW559768X | PKIS | GSK | –3 | 10 | 1 | 15 | 30 | 13 |
| 484 | SB-476429-A | PKIS | GSK | 1 | 13 | –42 | 16 | 4 | 20 |
| 485 | SB-610251-B | PKIS | GSK | 7 | 6 | –25 | 35 | –2 | 14 |
| 486 | GSK182497A | PKIS | GSK | 5 | 6 | –8 | 6 | –2 | 14 |
| 487 | GSK192082A | PKIS | GSK | 7 | 3 | –6 | 12 | –7 | 11 |
| 488 | GSK238063A | PKIS | GSK | 4 | 2 | 33 | 23 | –1 | 14 |
| 489 | GSK300014A | PKIS | GSK | –1 | 11 | 6 | 8 | 3 | 28 |
| 490 | GSK969786A | PKIS | GSK | –5 | 6 | 5 | 9 | –2 | 16 |
| 491 | GW684626B | PKIS | GSK | 6 | 8 | –18 | 4 | 2 | 22 |
| 492 | GW693881A | PKIS | GSK | 3 | 7 | –5 | 6 | 10 | 6 |
| 493 | GW784684X | PKIS | GSK | 22 | 17 | 22 | 60 | 7 | 37 |
| 494 | SB-675259-M* | PKIS | GSK | –8 | 10 | 52 | 10 | 20 | 33 |
| 495 | SB-678557-A | PKIS | GSK | 1 | 4 | 25 | 10 | –15 | 9 |
| 496 | SB-686709-A | PKIS | GSK | –6 | 11 | 35 | 12 | 7 | 19 |
| 497 | SB-698596-AC | PKIS | GSK | –3 | 5 | 38 | 7 | 19 | 19 |
| 498 | SB-711237 | PKIS | GSK | 1 | 4 | –7 | 10 | –8 | 18 |
| 499 | SB-732881* | PKIS | GSK | –1 | 10 | 37 | 8 | 41 | 14 |
| 500 | SB-732881-H* | PKIS | GSK | 0 | 5 | 41 | 20 | 65 | 16 |
| 501 | SB-739245-AC | PKIS | GSK | –1 | 9 | 7 | 15 | –8 | 12 |
| 502 | SB-743899 | PKIS | GSK | 6 | 9 | 5 | 7 | 2 | 7 |
| 503 | GW441756X | PKIS | GSK | 12 | 16 | 8 | 25 | 8 | 2 |
| 504 | GSK1173862A* | PKIS | GSK | 9 | 7 | 54 | 15 | 5 | 9 |
| 505 | GSK1220512A | PKIS | GSK | 1 | 11 | 22 | 29 | 8 | 8 |
| 506 | GSK1326255A | PKIS | GSK | 1 | 8 | 9 | 2 | 3 | 9 |
| 507 | GSK1392956A | PKIS | GSK | 10 | 21 | –8 | 4 | –1 | 36 |
| 508 | GSK994854A | PKIS | GSK | 7 | 7 | 10 | 7 | 3 | 5 |
| 509 | GW837331X | PKIS | GSK | 12 | 9 | 16 | 6 | –2 | 7 |
| 510 | GW861893X | PKIS | GSK | 10 | 6 | 5 | 11 | –3 | 14 |
| 511 | GW874091X | PKIS | GSK | 6 | 15 | –24 | 12 | 5 | 19 |
| 512 | GW513184X | PKIS | GSK | 10 | 6 | 0 | 2 | –5 | 36 |
| 513 | GW643971X | PKIS | GSK | 7 | 4 | –3 | 12 | –1 | 6 |
| 514 | GW801372X | PKIS | GSK | 6 | 6 | 3 | 15 | 29 | 30 |
| 515 | GW806290X* | PKIS | GSK | 60 | 7 | 70 | 5 | 68 | 5 |
| 516 | GW807982X | PKIS | GSK | 3 | 5 | 15 | 6 | 3 | 9 |
| 517 | GW810372X | PKIS | GSK | 18 | 12 | –6 | 7 | 7 | 8 |
| 518 | GW810576X* | PKIS | GSK | 44 | 33 | 24 | 32 | 47 | 38 |
| 519 | GW811761X | PKIS | GSK | 6 | 10 | –7 | 7 | 7 | 7 |
| 520 | GW819077X | PKIS | GSK | –2 | 8 | 4 | 4 | –8 | 12 |
| 521 | GW827099X | PKIS | GSK | 6 | 4 | 6 | 1 | 0 | 21 |
| 522 | GW827102X | PKIS | GSK | 15 | 17 | 11 | 25 | 6 | 18 |
| 523 | GW827105X | PKIS | GSK | 9 | 5 | –11 | 16 | –3 | 10 |
| 524 | GW827106X | PKIS | GSK | 10 | 5 | 1 | 10 | 3 | 5 |
| 525 | GW827396X | PKIS | GSK | –1 | 9 | –17 | 7 | –12 | 45 |
| 526 | GW828525X | PKIS | GSK | 8 | 7 | 1 | 7 | 3 | 7 |
| 527 | GW828529X | PKIS | GSK | 15 | 21 | 2 | 8 | 8 | 7 |
| 528 | GW829055X | PKIS | GSK | 5 | 7 | 12 | 8 | 14 | 6 |
| 529 | GW829115X | PKIS | GSK | 8 | 13 | –13 | 25 | 0 | 13 |
| 530 | GW829906X | PKIS | GSK | 1 | 2 | 10 | 3 | 9 | 8 |
| 531 | GW832467X | PKIS | GSK | 5 | 7 | 8 | 14 | 8 | 14 |
| 532 | GW833373X | PKIS | GSK | 9 | 13 | –3 | 5 | 17 | 5 |
| 533 | GSK1713088A | PKIS | GSK | 9 | 7 | 5 | 35 | –2 | 12 |
| 534 | GSK1751853A | PKIS | GSK | 6 | 6 | 31 | 15 | 4 | 6 |
| 535 | GSK2213727A* | PKIS | GSK | 3 | 2 | 38 | 10 | 14 | 11 |
| 536 | GSK2219385A | PKIS | GSK | 11 | 6 | 19 | 22 | –8 | 19 |
| 537 | GSK1511931A | PKIS | GSK | 17 | 20 | 13 | 21 | –1 | 8 |
| 538 | GSK1819799A | PKIS | GSK | 2 | 5 | 1 | 13 | –5 | 14 |
| 539 | GSK2110236A* | PKIS | GSK | 4 | 13 | 63 | 1 | –7 | 24 |
| 540 | GSK2163632A* | PKIS | GSK | 10 | 6 | 51 | 32 | –11 | 47 |
| 541 | GSK2186269A* | PKIS | GSK | 72 | 52 | 51 | 70 | 66 | 60 |
| 542 | GW279320X | PKIS | GSK | 3 | 4 | –7 | 10 | 5 | 12 |
| 543 | GW589933X | PKIS | GSK | 5 | 2 | –9 | 16 | 0 | 10 |
| 544 | GW561436X | PKIS | GSK | 5 | 6 | –4 | 26 | 0 | 12 |
| 545 | GW568326X | PKIS | GSK | 8 | 6 | 10 | 21 | –1 | 15 |
| 546 | GW581744X | PKIS | GSK | 8 | 8 | –3 | 7 | 5 | 5 |
| 547 | GW618013A | PKIS | GSK | 6 | 15 | –12 | 16 | 7 | 20 |
| 548 | GW683003X | PKIS | GSK | 15 | 8 | 6 | 6 | –15 | 14 |
| 549 | GW683109X | PKIS | GSK | 9 | 10 | 0 | 11 | –1 | 8 |
| 550 | GW683768X* | PKIS | GSK | –1 | 4 | 46 | 11 | 22 | 14 |
| 551 | GW708336X | PKIS | GSK | 5 | 5 | 1 | 4 | 2 | 6 |
| 552 | GW778894X* | PKIS | GSK | 58 | 5 | 55 | 20 | 73 | 1 |
| 553 | GW779439X* | PKIS | GSK | 59 | 3 | 60 | 29 | 65 | 7 |
| 554 | GW780056X* | PKIS | GSK | 63 | 10 | 46 | 48 | 69 | 6 |
| 555 | GW781673X | PKIS | GSK | 14 | 10 | 0 | 15 | –10 | 14 |
| 556 | GW805758X | PKIS | GSK | 7 | 15 | –9 | 15 | –9 | 3 |
| 557 | GW305074X | PKIS | GSK | 9 | 8 | –2 | 4 | 8 | 10 |
| 558 | GW405841X | PKIS | GSK | –3 | 3 | 0 | 4 | 14 | 18 |
| 559 | GW406108X | PKIS | GSK | 12 | 10 | –2 | 12 | 15 | 14 |
| 560 | GW407323A | PKIS | GSK | 4 | 2 | 7 | 26 | –19 | 40 |
| 561 | GW429374A | PKIS | GSK | 8 | 5 | 8 | 3 | 1 | 12 |
| 562 | SB-614067-R | PKIS | GSK | –11 | 17 | –28 | 18 | –15 | 27 |
| 563 | SB-682330-A | PKIS | GSK | 3 | 11 | –12 | 24 | –5 | 7 |
| 564 | GW434756X | PKIS | GSK | 8 | 10 | –2 | 10 | 5 | 9 |
| 565 | GW296115X | PKIS | GSK | 7 | 7 | –18 | 19 | 21 | 19 |
| 566 | GW284408X | PKIS | GSK | 7 | 6 | 4 | 12 | 0 | 17 |
| 567 | GW569293E | PKIS | GSK | 5 | 7 | –2 | 38 | –1 | 17 |
| 568 | SB-590885-AAD | PKIS | GSK | –2 | 5 | –25 | 12 | 1 | 5 |
| 569 | GW824645A | PKIS2 | GSK | 9 | 15 | 23 | 21 | 7 | 3 |
| 570 | GW659386A | PKIS2 | GSK | 1 | 5 | –27 | 2 | –3 | 31 |
| 571 | GW683134A | PKIS2 | GSK | 1 | 0 | 17 | 7 | 32 | 12 |
| 572 | GW574783B* | PKIS2 | GSK | 59 | 9 | 47 | 2 | 13 | 2 |
| 573 | GW779439X* | PKIS2 | GSK | 80 | 5 | 73 | 11 | 66 | 25 |
| 574 | AH20685XX | PKIS2 | GSK | 22 | 4 | 22 | 1 | 17 | 7 |
| 575 | AH2635 | PKIS2 | GSK | 10 | 6 | 18 | 10 | 9 | 14 |
| 576 | AH5015X | PKIS2 | GSK | 11 | 2 | 4 | 4 | 18 | 7 |
| 577 | GI230329A* | PKIS2 | GSK | 62 | 3 | 43 | 9 | 22 | 14 |
| 578 | GI261656A | PKIS2 | GSK | –4 | 1 | 9 | 5 | 20 | 6 |
| 579 | GI262866A | PKIS2 | GSK | 12 | 2 | 12 | 9 | 19 | 3 |
| 580 | GI98581X | PKIS2 | GSK | 3 | 2 | 21 | 5 | 12 | 14 |
| 581 | GSK1014915A | PKIS2 | GSK | 4 | 7 | –4 | 4 | 12 | 11 |
| 582 | GSK1024304A | PKIS2 | GSK | 33 | 9 | 22 | 10 | 11 | 23 |
| 583 | GSK1024306A | PKIS2 | GSK | 16 | 5 | 12 | 8 | 21 | 2 |
| 584 | GSK1033723A | PKIS2 | GSK | 9 | 5 | 16 | 5 | 10 | 15 |
| 585 | GSK1034945A | PKIS2 | GSK | 14 | 1 | 11 | 3 | 26 | 1 |
| 586 | GSK1122999D | PKIS2 | GSK | 8 | 3 | 14 | 5 | 16 | 13 |
| 587 | GSK1229496A | PKIS2 | GSK | 31 | 6 | 15 | 8 | 25 | 11 |
| 588 | GSK1229782A | PKIS2 | GSK | 21 | 3 | 17 | 6 | 4 | 12 |
| 589 | GSK1229959A* | PKIS2 | GSK | 89 | 2 | 69 | 8 | 84 | 1 |
| 590 | GSK1269851A | PKIS2 | GSK | 7 | 13 | 31 | 4 | 23 | 18 |
| 591 | GSK1287544A* | PKIS2 | GSK | 18 | 2 | 27 | 1 | 46 | 4 |
| 592 | GSK1292139B | PKIS2 | GSK | 8 | 0 | 25 | 30 | 17 | 1 |
| 593 | GSK1307810A* | PKIS2 | GSK | 12 | 1 | 34 | 6 | 49 | 9 |
| 594 | GSK1321561A | PKIS2 | GSK | –8 | 1 | 17 | 4 | 27 | 2 |
| 595 | GSK1321565A | PKIS2 | GSK | 13 | 8 | 30 | 1 | 31 | 4 |
| 596 | GSK1322949A* | PKIS2 | GSK | 38 | 2 | 53 | 3 | 82 | 6 |
| 597 | GSK1323434A | PKIS2 | GSK | 8 | 0 | 21 | 4 | 30 | 2 |
| 598 | GSK1325775A | PKIS2 | GSK | 10 | 2 | 31 | 12 | 35 | 19 |
| 599 | GSK1326180A | PKIS2 | GSK | –35 | 36 | 13 | 5 | 14 | 3 |
| 600 | GSK1379706A | PKIS2 | GSK | 7 | 6 | 20 | 0 | 4 | 19 |
| 601 | GSK1379708A | PKIS2 | GSK | 4 | 8 | 15 | 4 | 12 | 4 |
| 602 | GSK1379710A | PKIS2 | GSK | 9 | 3 | 7 | 13 | 7 | 18 |
| 603 | GSK1379712A | PKIS2 | GSK | 10 | 5 | 17 | 8 | 12 | 4 |
| 604 | GSK1379713A | PKIS2 | GSK | 5 | 5 | 19 | 3 | 3 | 11 |
| 605 | GSK1379714A | PKIS2 | GSK | 10 | 3 | 23 | 1 | 16 | 12 |
| 606 | GSK1379715A | PKIS2 | GSK | 26 | 6 | 27 | 13 | 11 | 8 |
| 607 | GSK1379716A | PKIS2 | GSK | 10 | 6 | 5 | 5 | 22 | 1 |
| 608 | GSK1379717A | PKIS2 | GSK | 15 | 1 | –3 | 10 | 9 | 3 |
| 609 | GSK1379720A | PKIS2 | GSK | 6 | 1 | 8 | 5 | 20 | 3 |
| 610 | GSK1379721A | PKIS2 | GSK | 13 | 2 | –1 | 18 | 20 | 10 |
| 611 | GSK1379722A | PKIS2 | GSK | 9 | 2 | 9 | 3 | 10 | 3 |
| 612 | GSK1379723A | PKIS2 | GSK | 10 | 1 | 11 | 4 | –2 | 16 |
| 613 | GSK1379724A | PKIS2 | GSK | 8 | 2 | 5 | 7 | 16 | 1 |
| 614 | GSK1379725A | PKIS2 | GSK | 7 | 1 | 9 | 3 | 10 | 1 |
| 615 | GSK1379727A | PKIS2 | GSK | 0 | 3 | 12 | 3 | 18 | 1 |
| 616 | GSK1379729A | PKIS2 | GSK | 3 | 5 | 8 | 7 | 18 | 14 |
| 617 | GSK1379730A | PKIS2 | GSK | 4 | 2 | 7 | 1 | 13 | 6 |
| 618 | GSK1379731A | PKIS2 | GSK | 6 | 1 | 9 | 16 | 9 | 9 |
| 619 | GSK1379732A | PKIS2 | GSK | 1 | 2 | 11 | 1 | 11 | 1 |
| 620 | GSK1379735A | PKIS2 | GSK | 4 | 4 | 11 | 4 | 4 | 6 |
| 621 | GSK1379737A* | PKIS2 | GSK | 30 | 1 | 40 | 12 | 18 | 2 |
| 622 | GSK1379738A | PKIS2 | GSK | 14 | 7 | 18 | 1 | 7 | 18 |
| 623 | GSK1379741A | PKIS2 | GSK | 6 | 1 | 11 | 0 | 6 | 4 |
| 624 | GSK1379742A | PKIS2 | GSK | 9 | 0 | 8 | 5 | 14 | 0 |
| 625 | GSK1379745A | PKIS2 | GSK | 5 | 2 | –2 | 14 | 7 | 2 |
| 626 | GSK1379746A | PKIS2 | GSK | 7 | 7 | 17 | 7 | 15 | 2 |
| 627 | GSK1379748A | PKIS2 | GSK | –1 | 15 | 10 | 3 | 20 | 2 |
| 628 | GSK1379751A | PKIS2 | GSK | 6 | 2 | 9 | 1 | 11 | 9 |
| 629 | GSK1379753A | PKIS2 | GSK | –8 | 15 | 5 | 0 | 9 | 0 |
| 630 | GSK1379754A | PKIS2 | GSK | 9 | 1 | 11 | 15 | 5 | 0 |
| 631 | GSK1379757A | PKIS2 | GSK | –22 | 2 | 3 | 15 | 10 | 0 |
| 632 | GSK1379760A | PKIS2 | GSK | 1 | 5 | 14 | 6 | 0 | 2 |
| 633 | GSK1379761A | PKIS2 | GSK | 3 | 6 | 16 | 2 | 16 | 8 |
| 634 | GSK1379762A | PKIS2 | GSK | 2 | 0 | 14 | 3 | 4 | 12 |
| 635 | GSK1379763A | PKIS2 | GSK | 6 | 11 | 15 | 5 | 8 | 12 |
| 636 | GSK1379765A | PKIS2 | GSK | –1 | 1 | 14 | 10 | 7 | 13 |
| 637 | GSK1379766A | PKIS2 | GSK | 16 | 5 | 20 | 2 | 18 | 15 |
| 638 | GSK1379767A | PKIS2 | GSK | 16 | 0 | 17 | 5 | 11 | 4 |
| 639 | GSK1379788A | PKIS2 | GSK | 9 | 1 | –8 | 12 | 18 | 11 |
| 640 | GSK1379800A | PKIS2 | GSK | 12 | 6 | –10 | 20 | 17 | 1 |
| 641 | GSK1379812A | PKIS2 | GSK | 0 | 12 | –11 | 7 | 13 | 2 |
| 642 | GSK1379825A | PKIS2 | GSK | 7 | 9 | –6 | 19 | 14 | 14 |
| 643 | GSK1379859A | PKIS2 | GSK | –24 | 6 | 5 | 5 | 9 | 4 |
| 644 | GSK1379860A | PKIS2 | GSK | 6 | 6 | –3 | 9 | 0 | 0 |
| 645 | GSK1379874A | PKIS2 | GSK | –12 | 0 | –3 | 2 | 7 | 2 |
| 646 | GSK1379878A | PKIS2 | GSK | –1 | 6 | 13 | 8 | 9 | 3 |
| 647 | GSK1379879A | PKIS2 | GSK | –6 | 13 | 4 | 10 | 12 | 2 |
| 648 | GSK1379880A | PKIS2 | GSK | 1 | 3 | 6 | 4 | 10 | 4 |
| 649 | GSK1379882A | PKIS2 | GSK | 2 | 0 | 7 | 6 | 8 | 1 |
| 650 | GSK1379883A | PKIS2 | GSK | 2 | 3 | 8 | 7 | 8 | 7 |
| 651 | GSK1379896A | PKIS2 | GSK | 5 | 2 | 5 | 0 | 11 | 1 |
| 652 | GSK1379899A | PKIS2 | GSK | 3 | 1 | 15 | 3 | 4 | 1 |
| 653 | GSK1379901A | PKIS2 | GSK | 16 | 1 | 25 | 11 | 21 | 5 |
| 654 | GSK1379944A | PKIS2 | GSK | 7 | 2 | 19 | 2 | 7 | 13 |
| 655 | GSK1383280A* | PKIS2 | GSK | 21 | 11 | 25 | 4 | 69 | 5 |
| 656 | GSK1383281A* | PKIS2 | GSK | 20 | 5 | 37 | 3 | 61 | 4 |
| 657 | GSK1389063A* | PKIS2 | GSK | 29 | 3 | 21 | 15 | 56 | 12 |
| 658 | GSK1398460A* | PKIS2 | GSK | 0 | 9 | 33 | 3 | 13 | 13 |
| 659 | GSK1398463A | PKIS2 | GSK | 0 | 24 | 24 | 0 | 8 | 1 |
| 660 | GSK1398467A | PKIS2 | GSK | 19 | 4 | 20 | 4 | –6 | 1 |
| 661 | GSK1398468A | PKIS2 | GSK | 1 | 12 | 9 | 10 | 6 | 0 |
| 662 | GSK1398470A* | PKIS2 | GSK | 16 | 2 | 39 | 1 | –2 | 36 |
| 663 | GSK1398471A | PKIS2 | GSK | –2 | 13 | 21 | 2 | 15 | 2 |
| 664 | GSK1398472A | PKIS2 | GSK | 12 | 1 | 30 | 5 | 6 | 20 |
| 665 | GSK1398473A | PKIS2 | GSK | 11 | 2 | 22 | 12 | 9 | 8 |
| 666 | GSK1398474A | PKIS2 | GSK | 18 | 3 | 31 | 11 | 14 | 2 |
| 667 | GSK1398475A* | PKIS2 | GSK | 24 | 5 | 42 | 4 | 13 | 1 |
| 668 | GSK1398477A* | PKIS2 | GSK | 13 | 5 | 32 | 2 | 24 | 1 |
| 669 | GSK1440913A | PKIS2 | GSK | 0 | 4 | 7 | 5 | 10 | 10 |
| 670 | GSK1487252A | PKIS2 | GSK | 29 | 4 | 24 | 5 | 38 | 10 |
| 671 | GSK1520489A* | PKIS2 | GSK | 62 | 10 | 60 | 13 | 90 | 6 |
| 672 | GSK1535721A | PKIS2 | GSK | 11 | 9 | –13 | 23 | 16 | 2 |
| 673 | GSK1558669A* | PKIS2 | GSK | 17 | 10 | 37 | 2 | 90 | 1 |
| 674 | GSK1576028A* | PKIS2 | GSK | 66 | 7 | 65 | 5 | 68 | 3 |
| 675 | GSK1581427A* | PKIS2 | GSK | 25 | 8 | 28 | 13 | 51 | 1 |
| 676 | GSK1581428A* | PKIS2 | GSK | 36 | 8 | 27 | 17 | 49 | 12 |
| 677 | GSK1627798A* | PKIS2 | GSK | 69 | 4 | 41 | 7 | 65 | 11 |
| 678 | GSK1645872A* | PKIS2 | GSK | 46 | 4 | 25 | 22 | 32 | 1 |
| 679 | GSK1645895A | PKIS2 | GSK | –10 | 12 | 3 | 9 | 15 | 5 |
| 680 | GSK1649598A* | PKIS2 | GSK | 11 | 7 | 12 | 6 | 45 | 10 |
| 681 | GSK1653537A | PKIS2 | GSK | –11 | 4 | 2 | 5 | 12 | 4 |
| 682 | GSK1653539A | PKIS2 | GSK | –10 | 4 | 12 | 3 | 4 | 3 |
| 683 | GSK1660437A* | PKIS2 | GSK | 29 | 1 | 17 | 12 | 43 | 4 |
| 684 | GSK1660450B* | PKIS2 | GSK | 37 | 3 | 19 | 2 | 28 | 7 |
| 685 | GSK1669917A | PKIS2 | GSK | 5 | 4 | 11 | 1 | 8 | 7 |
| 686 | GSK1669921A* | PKIS2 | GSK | 86 | 2 | 60 | 5 | 80 | 5 |
| 687 | GSK1693850A* | PKIS2 | GSK | 82 | 6 | 43 | 10 | 70 | 13 |
| 688 | GSK1723980B* | PKIS2 | GSK | 31 | 5 | –7 | 23 | 51 | 5 |
| 689 | GSK175726A | PKIS2 | GSK | –8 | 14 | –9 | 14 | –2 | 18 |
| 690 | GSK1804250A | PKIS2 | GSK | 2 | 16 | 18 | 28 | 22 | 21 |
| 691 | GSK189015A | PKIS2 | GSK | –14 | 23 | –3 | 18 | –5 | 9 |
| 692 | GSK190937A | PKIS2 | GSK | 2 | 7 | 9 | 20 | –2 | 22 |
| 693 | GSK1917008A | PKIS2 | GSK | –15 | 17 | –3 | 5 | 0 | 5 |
| 694 | GSK198271A* | PKIS2 | GSK | 67 | 1 | 28 | 9 | 11 | 3 |
| 695 | GSK2008607A* | PKIS2 | GSK | –6 | 16 | 9 | 6 | 50 | 5 |
| 696 | GSK204559A | PKIS2 | GSK | –1 | 3 | 17 | 12 | –2 | 6 |
| 697 | GSK204607A | PKIS2 | GSK | 3 | 1 | 12 | 2 | 11 | 4 |
| 698 | GSK204919A | PKIS2 | GSK | 4 | 6 | 25 | 10 | 15 | 1 |
| 699 | GSK205189A | PKIS2 | GSK | 8 | 4 | 17 | 5 | 10 | 1 |
| 700 | GSK2137462A | PKIS2 | GSK | 0 | 0 | –6 | 28 | 13 | 7 |
| 701 | GSK2177277A* | PKIS2 | GSK | 2 | 0 | 21 | 2 | 56 | 3 |
| 702 | GSK2181306A | PKIS2 | GSK | 14 | 8 | 11 | 4 | 5 | 4 |
| 703 | GSK2188764A | PKIS2 | GSK | 0 | 5 | 3 | 14 | 10 | 21 |
| 704 | GSK2189892A | PKIS2 | GSK | 15 | 11 | –7 | 24 | 14 | 17 |
| 705 | GSK2192730A | PKIS2 | GSK | 5 | 8 | –7 | 15 | 7 | 3 |
| 706 | GSK2193613A | PKIS2 | GSK | 11 | 7 | –9 | 20 | –5 | 14 |
| 707 | GSK2197149A | PKIS2 | GSK | –6 | 7 | –9 | 8 | 13 | 0 |
| 708 | GSK2206003A | PKIS2 | GSK | 3 | 2 | 5 | 12 | –6 | 22 |
| 709 | GSK2219329A | PKIS2 | GSK | –18 | 21 | –6 | 6 | 8 | 2 |
| 710 | GSK2221681A | PKIS2 | GSK | –4 | 4 | 7 | 9 | –3 | 19 |
| 711 | GSK2224810A | PKIS2 | GSK | –13 | 9 | –6 | 1 | 10 | 2 |
| 712 | GSK2225749A | PKIS2 | GSK | –5 | 4 | 21 | 11 | 15 | 15 |
| 713 | GSK2227430A | PKIS2 | GSK | 4 | 1 | 13 | 7 | 14 | 5 |
| 714 | GSK2228768A | PKIS2 | GSK | 3 | 2 | 18 | 9 | 10 | 13 |
| 715 | GSK223675A | PKIS2 | GSK | 7 | 4 | 30 | 1 | 22 | 4 |
| 716 | GSK223810A | PKIS2 | GSK | 7 | 0 | 15 | 0 | 4 | 11 |
| 717 | GSK2250882A | PKIS2 | GSK | 10 | 2 | 16 | 2 | 8 | 1 |
| 718 | GSK2258759A | PKIS2 | GSK | 14 | 10 | 11 | 1 | 10 | 7 |
| 719 | GSK2269905A | PKIS2 | GSK | 13 | 12 | 2 | 12 | 35 | 0 |
| 720 | GSK2276055A | PKIS2 | GSK | 28 | 5 | 6 | 6 | 9 | 13 |
| 721 | GSK2283293A | PKIS2 | GSK | –2 | 13 | –3 | 5 | 8 | 10 |
| 722 | GSK2286062A | PKIS2 | GSK | –3 | 12 | –3 | 8 | –21 | 19 |
| 723 | GSK2286096A | PKIS2 | GSK | 3 | 18 | –9 | 22 | 7 | 4 |
| 724 | GSK2286295A | PKIS2 | GSK | 11 | 3 | –3 | 15 | 1 | 10 |
| 725 | GSK2286775B | PKIS2 | GSK | –9 | 7 | –14 | 17 | 8 | 2 |
| 726 | GSK2288359A | PKIS2 | GSK | –11 | 0 | 0 | 20 | –20 | 12 |
| 727 | GSK2289044B | PKIS2 | GSK | 3 | 5 | 0 | 12 | 24 | 4 |
| 728 | GSK2291363A | PKIS2 | GSK | 10 | 4 | 15 | 12 | 20 | 18 |
| 729 | GSK2296823A | PKIS2 | GSK | 15 | 5 | 9 | 4 | 35 | 0 |
| 730 | GSK2297099A | PKIS2 | GSK | 14 | 1 | 7 | 11 | 19 | 28 |
| 731 | GSK2297428A | PKIS2 | GSK | 10 | 4 | 14 | 9 | 17 | 5 |
| 732 | GSK2297430A | PKIS2 | GSK | 13 | 2 | 15 | 9 | 20 | 11 |
| 733 | GSK2297542A | PKIS2 | GSK | 21 | 5 | 9 | 2 | 31 | 9 |
| 734 | GSK2297543A | PKIS2 | GSK | 20 | 0 | 14 | 9 | 18 | 7 |
| 735 | GSK2298859A | PKIS2 | GSK | 17 | 7 | –11 | 10 | 25 | 2 |
| 736 | GSK2299009A | PKIS2 | GSK | 4 | 5 | –12 | 20 | –11 | 9 |
| 737 | GSK2306394A | PKIS2 | GSK | 1 | 18 | –5 | 5 | 17 | 5 |
| 738 | GSK2328680A | PKIS2 | GSK | 3 | 1 | –6 | 33 | 9 | 6 |
| 739 | GSK2333389A | PKIS2 | GSK | 8 | 8 | –7 | 11 | 26 | 2 |
| 740 | GSK2334006A | PKIS2 | GSK | 1 | 2 | –12 | 12 | –1 | 20 |
| 741 | GSK2336394A | PKIS2 | GSK | –13 | 23 | –2 | 6 | 11 | 13 |
| 742 | GSK2342769A | PKIS2 | GSK | 10 | 7 | –12 | 8 | 9 | 25 |
| 743 | GSK2344444A | PKIS2 | GSK | 22 | 5 | –4 | 5 | 38 | 2 |
| 744 | GSK2347225A | PKIS2 | GSK | 11 | 1 | 0 | 15 | 11 | 19 |
| 745 | GSK2358994A | PKIS2 | GSK | 10 | 4 | –3 | 2 | 29 | 1 |
| 746 | GSK2363608B | PKIS2 | GSK | 17 | 5 | 18 | 1 | 28 | 13 |
| 747 | GSK2373690A* | PKIS2 | GSK | 31 | 3 | 17 | 4 | 45 | 4 |
| 748 | GSK2373693A | PKIS2 | GSK | 13 | 7 | 14 | 6 | 23 | 5 |
| 749 | GSK2373723A* | PKIS2 | GSK | 46 | 0 | 22 | 10 | 49 | 4 |
| 750 | GSK2375584A | PKIS2 | GSK | 30 | 2 | 23 | 0 | 33 | 5 |
| 751 | GSK2376236A | PKIS2 | GSK | 11 | 10 | –4 | 5 | 26 | 16 |
| 752 | GSK2576924A | PKIS2 | GSK | 12 | 6 | –14 | 6 | 9 | 7 |
| 753 | GSK257997A | PKIS2 | GSK | –7 | 20 | 9 | 20 | 0 | 15 |
| 754 | GSK2587663A | PKIS2 | GSK | –2 | 8 | 0 | 14 | –17 | 17 |
| 755 | GSK2592465A | PKIS2 | GSK | –4 | 24 | –7 | 20 | –11 | 3 |
| 756 | GSK2593067A | PKIS2 | GSK | –18 | 1 | –9 | 0 | –28 | 1 |
| 757 | GSK2593074A | PKIS2 | GSK | –11 | 9 | –11 | 19 | 2 | 6 |
| 758 | GSK260205A | PKIS2 | GSK | 7 | 5 | 5 | 13 | 9 | 0 |
| 759 | GSK2603346A | PKIS2 | GSK | –17 | 8 | 1 | 3 | 2 | 1 |
| 760 | GSK2603358A | PKIS2 | GSK | –10 | 16 | 14 | 2 | –12 | 24 |
| 761 | GSK2606414A | PKIS2 | GSK | 6 | 0 | 7 | 9 | 12 | 5 |
| 762 | GSK2606590A | PKIS2 | GSK | 4 | 6 | 25 | 6 | 14 | 7 |
| 763 | GSK2608885A | PKIS2 | GSK | 0 | 3 | 18 | 6 | 13 | 5 |
| 764 | GSK2608899A | PKIS2 | GSK | 3 | 0 | 19 | 5 | 9 | 7 |
| 765 | GSK2634140A | PKIS2 | GSK | 6 | 5 | 18 | 7 | 15 | 2 |
| 766 | GSK2634758A | PKIS2 | GSK | 14 | 4 | 17 | 8 | 3 | 6 |
| 767 | GSK2635225A | PKIS2 | GSK | –10 | 12 | –8 | 11 | 6 | 3 |
| 768 | GSK2645446A | PKIS2 | GSK | –5 | 17 | –22 | 19 | –9 | 9 |
| 769 | GSK292658A* | PKIS2 | GSK | 51 | 21 | 26 | 5 | 22 | 11 |
| 770 | GSK299495A* | PKIS2 | GSK | 63 | 5 | 27 | 27 | 19 | 19 |
| 771 | GSK301329A | PKIS2 | GSK | –4 | 14 | –5 | 2 | 5 | 12 |
| 772 | GSK301362A | PKIS2 | GSK | –5 | 2 | –2 | 17 | –32 | 21 |
| 773 | GSK306886A | PKIS2 | GSK | –10 | 21 | –7 | 4 | 1 | 9 |
| 774 | GSK312879A | PKIS2 | GSK | –2 | 1 | 5 | 0 | –17 | 15 |
| 775 | GSK323521A* | PKIS2 | GSK | 76 | 16 | 63 | 3 | 59 | 6 |
| 776 | GSK323543A* | PKIS2 | GSK | 84 | 0 | 81 | 6 | 40 | 27 |
| 777 | GSK326180A* | PKIS2 | GSK | 66 | 8 | 35 | 3 | 31 | 7 |
| 778 | GSK327238A* | PKIS2 | GSK | 83 | 2 | 80 | 2 | 62 | 9 |
| 779 | GSK336313A | PKIS2 | GSK | 0 | 5 | 7 | 4 | 8 | 3 |
| 780 | GSK336735A* | PKIS2 | GSK | 74 | 0 | 58 | 3 | 48 | 5 |
| 781 | GSK346294A* | PKIS2 | GSK | 19 | 7 | 18 | 2 | 43 | 19 |
| 782 | GSK350559A* | PKIS2 | GSK | 13 | 1 | 51 | 9 | 18 | 6 |
| 783 | GSK357952A* | PKIS2 | GSK | 41 | 14 | 19 | 8 | 20 | 14 |
| 784 | GSK361061A* | PKIS2 | GSK | 55 | 1 | 19 | 8 | 20 | 7 |
| 785 | GSK361065A | PKIS2 | GSK | –10 | 18 | –5 | 13 | –5 | 7 |
| 786 | GSK364507A | PKIS2 | GSK | –4 | 6 | –3 | 11 | –25 | 16 |
| 787 | GSK398099A | PKIS2 | GSK | –7 | 14 | –8 | 14 | –13 | 1 |
| 788 | GSK429286A | PKIS2 | GSK | –19 | 3 | 4 | 17 | –9 | 2 |
| 789 | GSK448459A* | PKIS2 | GSK | 11 | 6 | 23 | 4 | 55 | 31 |
| 790 | GSK479719A* | PKIS2 | GSK | 41 | 0 | 2 | 13 | 22 | 60 |
| 791 | GSK483724A* | PKIS2 | GSK | 44 | 7 | 33 | 4 | 65 | 13 |
| 792 | GSK507274A | PKIS2 | GSK | 3 | 13 | 3 | 14 | –3 | 18 |
| 793 | GSK507358A | PKIS2 | GSK | –15 | 2 | 11 | 6 | 8 | 6 |
| 794 | GSK534911A | PKIS2 | GSK | –4 | 4 | 32 | 8 | 30 | 6 |
| 795 | GSK534913A | PKIS2 | GSK | 0 | 1 | 28 | 10 | 27 | 0 |
| 796 | GSK562689A | PKIS2 | GSK | 20 | 4 | 22 | 15 | 22 | 12 |
| 797 | GSK580432A* | PKIS2 | GSK | 53 | 5 | 23 | 1 | 81 | 1 |
| 798 | GSK581271A | PKIS2 | GSK | 19 | 3 | 17 | 5 | 4 | 3 |
| 799 | GSK641502A* | PKIS2 | GSK | 61 | 49 | 22 | 1 | 71 | 2 |
| 800 | GSK683281A | PKIS2 | GSK | 6 | 6 | –5 | 30 | –16 | 9 |
| 801 | GSK846226A | PKIS2 | GSK | –12 | 25 | –8 | 10 | 8 | 3 |
| 802 | GSK902056A | PKIS2 | GSK | 3 | 6 | –1 | 19 | –15 | 27 |
| 803 | GSK907232A* | PKIS2 | GSK | 35 | 14 | 18 | 9 | 71 | 3 |
| 804 | GSK955403A | PKIS2 | GSK | –10 | 5 | –12 | 29 | –24 | 11 |
| 805 | GSK977617A | PKIS2 | GSK | –19 | 14 | 3 | 8 | 7 | 5 |
| 806 | GSK977620A | PKIS2 | GSK | –2 | 3 | 12 | 15 | –7 | 13 |
| 807 | GSK986310C | PKIS2 | GSK | –17 | 11 | –3 | 0 | 10 | 4 |
| 808 | GSK993273A | PKIS2 | GSK | 5 | 2 | 4 | 12 | –6 | 26 |
| 809 | GW271431X | PKIS2 | GSK | 0 | 7 | 21 | 7 | 14 | 1 |
| 810 | GW272142A | PKIS2 | GSK | –2 | 0 | 12 | 6 | 2 | 5 |
| 811 | GW273749A | PKIS2 | GSK | 4 | 6 | 19 | 1 | 13 | 2 |
| 812 | GW275568A | PKIS2 | GSK | –1 | 1 | 13 | 2 | –10 | 14 |
| 813 | GW281179X | PKIS2 | GSK | 22 | 9 | 19 | 9 | 21 | 8 |
| 814 | GW282450A | PKIS2 | GSK | 11 | 4 | 10 | 5 | 0 | 11 |
| 815 | GW284543A | PKIS2 | GSK | –9 | 17 | –10 | 6 | 8 | 14 |
| 816 | GW320571X | PKIS2 | GSK | 9 | 2 | –12 | 3 | –9 | 25 |
| 817 | GW345098X | PKIS2 | GSK | –4 | 15 | 1 | 11 | 2 | 3 |
| 818 | GW407034X | PKIS2 | GSK | 1 | 10 | –4 | 3 | –22 | 12 |
| 819 | GW412617A | PKIS2 | GSK | –12 | 18 | –10 | 13 | –11 | 4 |
| 820 | GW424170A | PKIS2 | GSK | –12 | 1 | –16 | 0 | –9 | 36 |
| 821 | GW440132A | PKIS2 | GSK | –12 | 11 | 4 | 10 | 0 | 1 |
| 822 | GW440135A | PKIS2 | GSK | –12 | 1 | –4 | 6 | –25 | 17 |
| 823 | GW440137A | PKIS2 | GSK | –10 | 3 | 2 | 6 | 8 | 10 |
| 824 | GW440138A* | PKIS2 | GSK | 21 | 14 | 39 | 1 | 26 | 30 |
| 825 | GW440146A | PKIS2 | GSK | 8 | 4 | 16 | 15 | 15 | 21 |
| 826 | GW440148A* | PKIS2 | GSK | 36 | 33 | 17 | 5 | 61 | 56 |
| 827 | GW457859A | PKIS2 | GSK | –3 | 1 | 8 | 11 | 10 | 4 |
| 828 | GW461484A | PKIS2 | GSK | 4 | 1 | 8 | 3 | 3 | 6 |
| 829 | GW461487A | PKIS2 | GSK | 16 | 2 | 7 | 1 | –1 | 0 |
| 830 | GW466413A | PKIS2 | GSK | –17 | 22 | –21 | 8 | –6 | 11 |
| 831 | GW468513X | PKIS2 | GSK | 1 | 5 | –22 | 10 | 8 | 6 |
| 832 | GW475620X | PKIS2 | GSK | –10 | 20 | –12 | 6 | 6 | 6 |
| 833 | GW482059X | PKIS2 | GSK | –7 | 3 | –22 | 14 | –24 | 11 |
| 834 | GW493036X | PKIS2 | GSK | 11 | 18 | 1 | 1 | –2 | 9 |
| 835 | GW494601A | PKIS2 | GSK | –9 | 0 | 2 | 18 | –27 | 10 |
| 836 | GW494610A | PKIS2 | GSK | –23 | 2 | –6 | 0 | 19 | 1 |
| 837 | GW494702A | PKIS2 | GSK | –11 | 4 | –16 | 27 | –27 | 25 |
| 838 | GW497681X | PKIS2 | GSK | –31 | 9 | –7 | 5 | –9 | 11 |
| 839 | GW514784X | PKIS2 | GSK | –3 | 4 | –1 | 6 | –11 | 20 |
| 840 | GW514786X | PKIS2 | GSK | 5 | 4 | 7 | 2 | 13 | 2 |
| 841 | GW515532X | PKIS2 | GSK | 2 | 3 | 15 | 11 | 12 | 8 |
| 842 | GW525701A* | PKIS2 | GSK | 33 | 7 | 50 | 3 | 75 | 0 |
| 843 | GW548057X | PKIS2 | GSK | 0 | 6 | 19 | 2 | 7 | 1 |
| 844 | GW551191X | PKIS2 | GSK | –2 | 6 | 6 | 6 | 9 | 2 |
| 845 | GW552771X | PKIS2 | GSK | 7 | 4 | 9 | 0 | 2 | 14 |
| 846 | GW554060X | PKIS2 | GSK | 1 | 8 | –11 | 9 | –1 | 17 |
| 847 | GW557777X | PKIS2 | GSK | –12 | 5 | 7 | 7 | 37 | 3 |
| 848 | GW560106X | PKIS2 | GSK | –8 | 17 | –1 | 7 | 8 | 4 |
| 849 | GW560109X | PKIS2 | GSK | –14 | 6 | –5 | 11 | –6 | 14 |
| 850 | GW560116X | PKIS2 | GSK | –18 | 22 | –10 | 11 | 5 | 11 |
| 851 | GW560459X | PKIS2 | GSK | –14 | 1 | –16 | 0 | –35 | 12 |
| 852 | GW567140X | PKIS2 | GSK | –17 | 12 | –4 | 0 | –10 | 3 |
| 853 | GW567142A | PKIS2 | GSK | –11 | 6 | –6 | 4 | –15 | 17 |
| 854 | GW567143X | PKIS2 | GSK | –19 | 10 | –6 | 3 | –3 | 5 |
| 855 | GW567145X | PKIS2 | GSK | –14 | 15 | 3 | 12 | –9 | 31 |
| 856 | GW567148X | PKIS2 | GSK | –8 | 1 | 1 | 4 | 14 | 2 |
| 857 | GW569716A* | PKIS2 | GSK | 50 | 0 | 40 | 9 | 13 | 4 |
| 858 | GW576604X | PKIS2 | GSK | –1 | 1 | 20 | 12 | 13 | 2 |
| 859 | GW577382X | PKIS2 | GSK | –8 | 3 | –6 | 2 | 1 | 4 |
| 860 | GW578342X | PKIS2 | GSK | 8 | 4 | 1 | 7 | –1 | 4 |
| 861 | GW579362A | PKIS2 | GSK | 3 | 24 | 19 | 6 | 20 | 8 |
| 862 | GW582764A* | PKIS2 | GSK | 44 | 14 | 6 | 4 | 21 | 17 |
| 863 | GW582868A | PKIS2 | GSK | –8 | 10 | –4 | 2 | 10 | 5 |
| 864 | GW583340C* | PKIS2 | GSK | 44 | 3 | 24 | 3 | –13 | 10 |
| 865 | GW591947A | PKIS2 | GSK | –12 | 18 | –8 | 3 | –5 | 7 |
| 866 | GW595885X | PKIS2 | GSK | –3 | 4 | –1 | 18 | –19 | 26 |
| 867 | GW599550X | PKIS2 | GSK | –22 | 8 | –1 | 13 | –5 | 4 |
| 868 | GW608005X | PKIS2 | GSK | 2 | 2 | –12 | 30 | –22 | 21 |
| 869 | GW621581X | PKIS2 | GSK | –9 | 4 | 0 | 8 | 0 | 0 |
| 870 | GW622475X | PKIS2 | GSK | –7 | 6 | –5 | 21 | 10 | 11 |
| 871 | GW630813X | PKIS2 | GSK | –2 | 15 | 6 | 1 | 13 | 2 |
| 872 | GW630823A | PKIS2 | GSK | –1 | 4 | 3 | 8 | 14 | 2 |
| 873 | GW635815X | PKIS2 | GSK | 2 | 5 | 15 | 0 | 15 | 2 |
| 874 | GW639905A | PKIS2 | GSK | 2 | 6 | –7 | 42 | 1 | 4 |
| 875 | GW654607A | PKIS2 | GSK | 1 | 3 | 7 | 8 | 17 | 5 |
| 876 | GW659008A | PKIS2 | GSK | 13 | 7 | 10 | 5 | –6 | 9 |
| 877 | GW659009A | PKIS2 | GSK | –2 | 5 | –6 | 6 | –1 | 10 |
| 878 | GW663929X | PKIS2 | GSK | 0 | 14 | 4 | 6 | 11 | 10 |
| 879 | GW664114X | PKIS2 | GSK | 2 | 0 | 14 | 5 | 13 | 11 |
| 880 | GW679395X | PKIS2 | GSK | 3 | 16 | 0 | 7 | –12 | 2 |
| 881 | GW679396X | PKIS2 | GSK | –10 | 1 | –12 | 0 | –24 | 16 |
| 882 | GW679662X | PKIS2 | GSK | –8 | 1 | –10 | 13 | –11 | 1 |
| 883 | GW680061X | PKIS2 | GSK | –6 | 11 | 2 | 3 | –21 | 32 |
| 884 | GW680338X | PKIS2 | GSK | –19 | 5 | 9 | 1 | –2 | 3 |
| 885 | GW680903X | PKIS2 | GSK | 0 | 24 | 13 | 22 | 22 | 62 |
| 886 | GW681170A | PKIS2 | GSK | –6 | 2 | 6 | 2 | –1 | 1 |
| 887 | GW681251X | PKIS2 | GSK | –7 | 5 | 8 | 14 | 11 | 8 |
| 888 | GW682569X | PKIS2 | GSK | 10 | 2 | 23 | 9 | 18 | 3 |
| 889 | GW684083X | PKIS2 | GSK | 10 | 9 | 22 | 1 | 25 | 28 |
| 890 | GW684088X | PKIS2 | GSK | –2 | 7 | 18 | 0 | 38 | 49 |
| 891 | GW684374X | PKIS2 | GSK | 7 | 10 | 4 | 1 | 23 | 40 |
| 892 | GW684941X | PKIS2 | GSK | 23 | 29 | 21 | 1 | 24 | 35 |
| 893 | GW689066X | PKIS2 | GSK | 8 | 5 | 9 | 5 | 15 | 25 |
| 894 | GW692089A | PKIS2 | GSK | –4 | 17 | 0 | 12 | 14 | 25 |
| 895 | GW693028X | PKIS2 | GSK | 3 | 5 | 19 | 7 | 21 | 21 |
| 896 | GW693542X | PKIS2 | GSK | 12 | 1 | 16 | 5 | –4 | 10 |
| 897 | GW694077X | PKIS2 | GSK | 23 | 9 | 20 | 3 | 43 | 27 |
| 898 | GW696155X | PKIS2 | GSK | 23 | 22 | 24 | 4 | 27 | 8 |
| 899 | GW697465A | PKIS2 | GSK | 6 | 6 | 6 | 5 | 8 | 8 |
| 900 | GW697999A | PKIS2 | GSK | 9 | 15 | 13 | 2 | 16 | 1 |
| 901 | GW701424A* | PKIS2 | GSK | 35 | 39 | 25 | 2 | 36 | 36 |
| 902 | GW702865X | PKIS2 | GSK | –9 | 3 | 0 | 1 | 1 | 3 |
| 903 | GW707818B* | PKIS2 | GSK | 26 | 2 | 34 | 9 | 9 | 8 |
| 904 | GW709199X | PKIS2 | GSK | –7 | 20 | 10 | 4 | 9 | 10 |
| 905 | GW709213X | PKIS2 | GSK | 4 | 28 | –3 | 6 | 40 | 37 |
| 906 | GW767488X* | PKIS2 | GSK | 41 | 46 | –4 | 12 | 37 | 49 |
| 907 | GW768504A | PKIS2 | GSK | 5 | 10 | 6 | 12 | 5 | 10 |
| 908 | GW775604X | PKIS2 | GSK | 9 | 4 | 27 | 24 | 0 | 4 |
| 909 | GW775610X | PKIS2 | GSK | 3 | 0 | 1 | 8 | 1 | 1 |
| 910 | GW776245A | PKIS2 | GSK | 9 | 0 | 21 | 6 | 10 | 3 |
| 911 | GW777257X* | PKIS2 | GSK | 21 | 9 | 36 | 3 | 34 | 12 |
| 912 | GW781483X* | PKIS2 | GSK | 31 | 10 | 43 | 0 | 45 | 13 |
| 913 | GW784041A* | PKIS2 | GSK | 5 | 34 | 35 | 2 | 36 | 10 |
| 914 | GW787226A | PKIS2 | GSK | 14 | 42 | 6 | 16 | 22 | 23 |
| 915 | GW789449X | PKIS2 | GSK | 9 | 37 | 2 | 29 | 29 | 9 |
| 916 | GW792479X | PKIS2 | GSK | 6 | 33 | 12 | 12 | 30 | 18 |
| 917 | GW800172X | PKIS2 | GSK | 9 | 11 | 9 | 5 | 27 | 32 |
| 918 | GW809893X* | PKIS2 | GSK | 27 | 56 | 35 | 4 | 61 | 15 |
| 919 | GW810083X | PKIS2 | GSK | 3 | 14 | 15 | 5 | 22 | 18 |
| 920 | GW810437X | PKIS2 | GSK | 2 | 5 | 18 | 1 | 15 | 24 |
| 921 | GW810445X | PKIS2 | GSK | –4 | 19 | 16 | 2 | –3 | 13 |
| 922 | GW810578X* | PKIS2 | GSK | 72 | 9 | 30 | 17 | 40 | 34 |
| 923 | GW811603A | PKIS2 | GSK | –6 | 3 | –2 | 1 | 4 | 19 |
| 924 | GW812171X | PKIS2 | GSK | 7 | 9 | 17 | 8 | 3 | 17 |
| 925 | GW813244A | PKIS2 | GSK | –6 | 1 | 3 | 1 | –1 | 9 |
| 926 | GW813349X | PKIS2 | GSK | 0 | 1 | 2 | 1 | 9 | 22 |
| 927 | GW818933X | PKIS2 | GSK | 3 | 3 | 6 | 1 | 9 | 18 |
| 928 | GW818941X | PKIS2 | GSK | 3 | 9 | 9 | 8 | 16 | 27 |
| 929 | GW819776X | PKIS2 | GSK | 5 | 2 | 3 | 10 | –4 | 11 |
| 930 | GW823670X | PKIS2 | GSK | –11 | 3 | –5 | 28 | –2 | 12 |
| 931 | GW827654A | PKIS2 | GSK | –9 | 5 | 2 | 8 | 16 | 3 |
| 932 | GW828205X | PKIS2 | GSK | –11 | 7 | 3 | 6 | 2 | 13 |
| 933 | GW828206X | PKIS2 | GSK | –6 | 9 | 4 | 0 | 12 | 5 |
| 934 | GW829058X | PKIS2 | GSK | 2 | 14 | 16 | 7 | 10 | 1 |
| 935 | GW829116X | PKIS2 | GSK | 29 | 13 | –8 | 5 | 13 | 26 |
| 936 | GW829350X | PKIS2 | GSK | –2 | 6 | –3 | 8 | 5 | 2 |
| 937 | GW829351X | PKIS2 | GSK | 3 | 1 | –3 | 6 | –1 | 8 |
| 938 | GW830707A | PKIS2 | GSK | 7 | 18 | 21 | 23 | 13 | 3 |
| 939 | GW830899A | PKIS2 | GSK | 6 | 3 | 13 | 0 | 9 | 2 |
| 940 | GW835314X | PKIS2 | GSK | –3 | 25 | 18 | 17 | 16 | 13 |
| 941 | GW839464X | PKIS2 | GSK | 8 | 30 | 8 | 6 | 24 | 17 |
| 942 | GW854278X | PKIS2 | GSK | –14 | 31 | 21 | 1 | 18 | 32 |
| 943 | GW855857X* | PKIS2 | GSK | –9 | 37 | 34 | 3 | 49 | 34 |
| 944 | GW856795X | PKIS2 | GSK | –7 | 50 | 10 | 3 | 15 | 24 |
| 945 | GW856805X | PKIS2 | GSK | –16 | 74 | 12 | 2 | 23 | 14 |
| 946 | GW857175X | PKIS2 | GSK | 17 | 30 | 30 | 27 | 35 | 41 |
| 947 | GW867253X | PKIS2 | GSK | 9 | 41 | 7 | 28 | 24 | 15 |
| 948 | GW867587X | PKIS2 | GSK | 13 | 9 | 27 | 1 | 24 | 21 |
| 949 | GW867588X | PKIS2 | GSK | –13 | 22 | 21 | 2 | 7 | 3 |
| 950 | GW868318X | PKIS2 | GSK | –9 | 4 | –9 | 6 | 19 | 22 |
| 951 | GW869516X | PKIS2 | GSK | 20 | 18 | 21 | 5 | 26 | 19 |
| 952 | GW869640X* | PKIS2 | GSK | –10 | 10 | 33 | 6 | 11 | 13 |
| 953 | GW869641X | PKIS2 | GSK | 3 | 6 | 15 | 23 | 2 | 18 |
| 954 | GW869979X | PKIS2 | GSK | –2 | 3 | –3 | 7 | 10 | 23 |
| 955 | GW872411X* | PKIS2 | GSK | 22 | 1 | 32 | 5 | 16 | 9 |
| 956 | GW873004X | PKIS2 | GSK | 1 | 15 | 2 | 1 | 7 | 6 |
| 957 | GW876019X | PKIS2 | GSK | 11 | 5 | 28 | 16 | 0 | 14 |
| 958 | GW876731X* | PKIS2 | GSK | 37 | 17 | 41 | 5 | 58 | 13 |
| 959 | SB-202620 | PKIS2 | GSK | –5 | 12 | 0 | 7 | 4 | 11 |
| 960 | SB-210486 | PKIS2 | GSK | –1 | 18 | 5 | 0 | –9 | 13 |
| 961 | SB-211742 | PKIS2 | GSK | –4 | 11 | –5 | 5 | –7 | 10 |
| 962 | SB-211743 | PKIS2 | GSK | 14 | 15 | 24 | 23 | 14 | 3 |
| 963 | SB-213663 | PKIS2 | GSK | –26 | 12 | 4 | 1 | 8 | 0 |
| 964 | SB-217146-A | PKIS2 | GSK | –9 | 23 | 0 | 8 | 5 | 10 |
| 965 | SB-217360 | PKIS2 | GSK | –12 | 15 | –1 | 5 | 9 | 1 |
| 966 | SB-217780 | PKIS2 | GSK | –6 | 16 | –2 | 3 | 23 | 13 |
| 967 | SB-219952 | PKIS2 | GSK | –4 | 21 | –4 | 5 | 5 | 13 |
| 968 | SB-219980 | PKIS2 | GSK | 4 | 15 | –10 | 2 | 8 | 3 |
| 969 | SB-222516 | PKIS2 | GSK | 2 | 12 | –1 | 7 | 6 | 9 |
| 970 | SB-222517 | PKIS2 | GSK | –4 | 16 | 10 | 20 | –5 | 10 |
| 971 | SB-222903 | PKIS2 | GSK | –13 | 27 | 4 | 4 | 1 | 10 |
| 972 | SB-223132 | PKIS2 | GSK | –10 | 20 | –5 | 8 | –10 | 8 |
| 973 | SB-226605 | PKIS2 | GSK | 1 | 36 | 6 | 1 | 28 | 18 |
| 974 | SB-229482 | PKIS2 | GSK | –28 | 46 | 12 | 0 | 4 | 13 |
| 975 | SB-236560 | PKIS2 | GSK | –26 | 51 | –2 | 9 | 25 | 6 |
| 976 | SB-238039-R | PKIS2 | GSK | 30 | 15 | 11 | 1 | 33 | 27 |
| 977 | SB-245391 | PKIS2 | GSK | –5 | 28 | 6 | 15 | 1 | 12 |
| 978 | SB-249175 | PKIS2 | GSK | –11 | 30 | –2 | 10 | 0 | 13 |
| 979 | SB-282852 | PKIS2 | GSK | –1 | 28 | 1 | 6 | 8 | 15 |
| 980 | SB-282975-A | PKIS2 | GSK | 9 | 5 | 6 | 1 | 14 | 19 |
| 981 | SB-284851-BT | PKIS2 | GSK | –9 | 37 | 0 | 14 | 2 | 9 |
| 982 | SB-284852-BT | PKIS2 | GSK | –5 | 31 | –9 | 11 | 11 | 14 |
| 983 | SB-300079 | PKIS2 | GSK | 6 | 8 | 12 | 1 | 1 | 4 |
| 984 | SB-317651 | PKIS2 | GSK | –3 | 10 | 21 | 7 | 16 | 20 |
| 985 | SB-317658 | PKIS2 | GSK | –9 | 1 | –13 | 10 | –6 | 16 |
| 986 | SB-317661 | PKIS2 | GSK | 5 | 9 | 7 | 7 | 5 | 10 |
| 987 | SB-326892 | PKIS2 | GSK | 15 | 1 | –4 | 3 | –3 | 15 |
| 988 | SB-331032 | PKIS2 | GSK | 2 | 7 | –1 | 1 | 5 | 17 |
| 989 | SB-333613 | PKIS2 | GSK | 11 | 2 | 10 | 5 | –2 | 12 |
| 990 | SB-334860 | PKIS2 | GSK | 9 | 4 | 7 | 2 | 9 | 14 |
| 991 | SB-334865 | PKIS2 | GSK | 1 | 8 | –5 | 16 | –14 | 6 |
| 992 | SB-340867 | PKIS2 | GSK | 0 | 11 | –9 | 2 | –2 | 32 |
| 993 | SB-341528 | PKIS2 | GSK | –7 | 3 | –2 | 5 | –11 | 11 |
| 994 | SB-341556 | PKIS2 | GSK | 8 | 4 | 18 | 17 | 1 | 3 |
| 995 | SB-342409 | PKIS2 | GSK | –20 | 4 | 7 | 1 | 12 | 3 |
| 996 | SB-342411 | PKIS2 | GSK | –13 | 18 | 0 | 7 | 1 | 6 |
| 997 | SB-360737 | PKIS2 | GSK | –7 | 6 | –8 | 10 | –9 | 12 |
| 998 | SB-373598 | PKIS2 | GSK | 3 | 4 | –1 | 5 | –3 | 11 |
| 999 | SB-376715 | PKIS2 | GSK | –18 | 28 | –10 | 1 | –3 | 2 |
| 1000 | SB-381891 | PKIS2 | GSK | –5 | 10 | –5 | 1 | 8 | 1 |
| 1001 | SB-381904 | PKIS2 | GSK | 2 | 13 | 1 | 10 | 6 | 11 |
| 1002 | SB-386023-B | PKIS2 | GSK | –13 | 14 | –8 | 13 | –8 | 3 |
| 1003 | SB-390526 | PKIS2 | GSK | 0 | 7 | –3 | 0 | –15 | 19 |
| 1004 | SB-390530 | PKIS2 | GSK | 7 | 4 | –3 | 7 | –13 | 14 |
| 1005 | SB-390532 | PKIS2 | GSK | –1 | 24 | 3 | 3 | 18 | 26 |
| 1006 | SB-390534 | PKIS2 | GSK | –31 | 35 | –5 | 13 | 5 | 16 |
| 1007 | SB-390765 | PKIS2 | GSK | –31 | 40 | 0 | 17 | 0 | 12 |
| 1008 | SB-390766 | PKIS2 | GSK | –9 | 20 | 3 | 12 | 16 | 26 |
| 1009 | SB-390767 | PKIS2 | GSK | –22 | 21 | –11 | 18 | 13 | 9 |
| 1010 | SB-390769 | PKIS2 | GSK | –7 | 20 | 5 | 4 | –10 | 15 |
| 1011 | SB-390770 | PKIS2 | GSK | 0 | 14 | 2 | 12 | 0 | 13 |
| 1012 | SB-390771 | PKIS2 | GSK | 15 | 7 | 10 | 1 | 28 | 23 |
| 1013 | SB-404290 | PKIS2 | GSK | –14 | 0 | 0 | 5 | –5 | 4 |
| 1014 | SB-404321 | PKIS2 | GSK | –4 | 17 | –23 | 15 | –12 | 4 |
| 1015 | SB-405367 | PKIS2 | GSK | 13 | 3 | 18 | 2 | –3 | 11 |
| 1016 | SB-408010 | PKIS2 | GSK | –11 | 12 | 7 | 28 | 12 | 17 |
| 1017 | SB-428218-A | PKIS2 | GSK | –1 | 0 | –11 | 13 | –10 | 13 |
| 1018 | SB-477794-AAA | PKIS2 | GSK | –1 | 41 | –4 | 6 | 22 | 11 |
| 1019 | SB-517081 | PKIS2 | GSK | 6 | 5 | –5 | 1 | –9 | 12 |
| 1020 | SB-517389 | PKIS2 | GSK | –1 | 11 | –3 | 15 | –6 | 11 |
| 1021 | SB-548492 | PKIS2 | GSK | 7 | 5 | 6 | 1 | –6 | 10 |
| 1022 | SB-589132 | PKIS2 | GSK | 1 | 3 | 5 | 2 | –6 | 9 |
| 1023 | SB-601273* | PKIS2 | GSK | 49 | 46 | 12 | 12 | 44 | 52 |
| 1024 | SB-601436 | PKIS2 | GSK | –2 | 10 | –7 | 2 | –10 | 8 |
| 1025 | SB-610250 | PKIS2 | GSK | –2 | 14 | –14 | 7 | –8 | 8 |
| 1026 | SB-625086-M | PKIS2 | GSK | 9 | 6 | 17 | 15 | 8 | 4 |
| 1027 | SB-627772-A | PKIS2 | GSK | –15 | 1 | 3 | 10 | 14 | 3 |
| 1028 | SB-642057 | PKIS2 | GSK | –7 | 5 | 17 | 13 | 5 | 17 |
| 1029 | SB-642124-AAA | PKIS2 | GSK | –6 | 3 | –6 | 2 | –8 | 0 |
| 1030 | SB-660566 | PKIS2 | GSK | –19 | 12 | –8 | 0 | –5 | 1 |
| 1031 | SB-684387-B | PKIS2 | GSK | –21 | 27 | –11 | 4 | –11 | 19 |
| 1032 | SB-693162 | PKIS2 | GSK | 1 | 12 | –9 | 1 | 3 | 2 |
| 1033 | SB-693578 | PKIS2 | GSK | 3 | 7 | –5 | 1 | 3 | 7 |
| 1034 | SB-707548-A | PKIS2 | GSK | 13 | 10 | 25 | 3 | –1 | 10 |
| 1035 | SB-708998 | PKIS2 | GSK | 3 | 8 | –3 | 6 | –8 | 31 |
| 1036 | SB-708999 | PKIS2 | GSK | 3 | 12 | 0 | 1 | –20 | 7 |
| 1037 | SB-710363 | PKIS2 | GSK | 5 | 28 | 14 | 1 | 27 | 12 |
| 1038 | SB-710397-B | PKIS2 | GSK | –24 | 34 | –2 | 6 | 12 | 12 |
| 1039 | SB-710903 | PKIS2 | GSK | –31 | 40 | –21 | 19 | 11 | 2 |
| 1040 | SB-711239 | PKIS2 | GSK | –23 | 26 | –16 | 27 | 5 | 18 |
| 1041 | SB-711805 | PKIS2 | GSK | –17 | 28 | –6 | 21 | –4 | 9 |
| 1042 | SB-711880 | PKIS2 | GSK | –19 | 26 | –2 | 14 | –7 | 15 |
| 1043 | SB-731254-M | PKIS2 | GSK | 14 | 4 | 2 | 3 | 5 | 19 |
| 1044 | SB-731284 | PKIS2 | GSK | 7 | 6 | 2 | 3 | 5 | 26 |
| 1045 | SB-731579 | PKIS2 | GSK | –19 | 34 | 9 | 22 | 22 | 25 |
| 1046 | SB-732932 | PKIS2 | GSK | –17 | 17 | –7 | 9 | –12 | 14 |
| 1047 | SB-733371 | PKIS2 | GSK | 5 | 7 | 12 | 1 | –5 | 11 |
| 1048 | SB-733416 | PKIS2 | GSK | –4 | 14 | 0 | 1 | 10 | 17 |
| 1049 | SB-733887 | PKIS2 | GSK | –27 | 3 | –16 | 25 | –16 | 22 |
| 1050 | SB-733894 | PKIS2 | GSK | –16 | 2 | –4 | 1 | –11 | 9 |
| 1051 | SB-734909 | PKIS2 | GSK | –14 | 2 | –11 | 0 | –22 | 30 |
| 1052 | SB-735216 | PKIS2 | GSK | 3 | 4 | –2 | 1 | –13 | 14 |
| 1053 | SB-735297 | PKIS2 | GSK | 6 | 6 | 1 | 7 | –7 | 9 |
| 1054 | SB-735464 | PKIS2 | GSK | 5 | 6 | 12 | 2 | 12 | 25 |
| 1055 | SB-736398 | PKIS2 | GSK | –3 | 3 | 1 | 1 | –8 | 8 |
| 1056 | SB-736715 | PKIS2 | GSK | –5 | 4 | –5 | 4 | 6 | 18 |
| 1057 | SB-737447 | PKIS2 | GSK | –7 | 10 | –9 | 11 | –2 | 15 |
| 1058 | SB-737856 | PKIS2 | GSK | 12 | 8 | 9 | 9 | 2 | 3 |
| 1059 | SB-738004 | PKIS2 | GSK | –4 | 4 | 10 | 27 | 26 | 2 |
| 1060 | SB-738481 | PKIS2 | GSK | –23 | 3 | 6 | 16 | –11 | 7 |
| 1061 | SB-742034-AC | PKIS2 | GSK | –15 | 1 | –1 | 11 | –20 | 26 |
| 1062 | SB-742251 | PKIS2 | GSK | –9 | 12 | –11 | 1 | –14 | 2 |
| 1063 | SB-742352-AC | PKIS2 | GSK | –19 | 35 | –8 | 2 | –10 | 5 |
| 1064 | SB-742609 | PKIS2 | GSK | 2 | 10 | –10 | 0 | 4 | 6 |
| 1065 | SB-743341 | PKIS2 | GSK | 0 | 10 | –1 | 6 | –1 | 6 |
| 1066 | SB-747651-A | PKIS2 | GSK | 25 | 12 | 24 | 21 | 21 | 7 |
| 1067 | SB-750250-M | PKIS2 | GSK | 0 | 13 | –4 | 1 | –8 | 14 |
| 1068 | SKF-104365 | PKIS2 | GSK | 9 | 7 | 32 | 7 | 10 | 4 |
| 1069 | SKF-104493-B2 | PKIS2 | GSK | 7 | 19 | 7 | 4 | 21 | 22 |
| 1070 | SKF-105561 | PKIS2 | GSK | –33 | 40 | 3 | 8 | 1 | 12 |
| 1071 | SKF-105942 | PKIS2 | GSK | –31 | 26 | –18 | 18 | –15 | 8 |
| 1072 | SKF-106164-A2 | PKIS2 | GSK | –15 | 18 | –11 | 35 | –5 | 6 |
| 1073 | SKF-12778 | PKIS2 | GSK | –13 | 27 | –21 | 20 | –2 | 10 |
| 1074 | SKF-18267 | PKIS2 | GSK | –14 | 21 | –9 | 3 | 14 | 17 |
| 1075 | SKF-18355 | PKIS2 | GSK | 7 | 6 | 2 | 10 | 0 | 14 |
| 1076 | SKF-31736 | PKIS2 | GSK | 19 | 21 | –1 | 9 | 22 | 25 |
| 1077 | SKF-96418 | PKIS2 | GSK | –24 | 34 | 2 | 1 | –4 | 9 |
| 1078 | SKF-97184 | PKIS2 | GSK | –27 | 58 | 10 | 14 | –5 | 5 |
| 1079 | SKF-97236 | PKIS2 | GSK | 11 | 7 | 14 | 2 | 10 | 14 |
| 1080 | SKF-97255 | PKIS2 | GSK | –27 | 18 | 7 | 12 | –5 | 12 |
| 1081 | SKF-97263 | PKIS2 | GSK | –12 | 9 | –18 | 3 | –13 | 19 |
| 1082 | SKF-97293 | PKIS2 | GSK | –4 | 6 | –5 | 27 | 4 | 15 |
| 1083 | SKF-97359 | PKIS2 | GSK | 1 | 7 | –7 | 4 | –12 | 11 |
| 1084 | SKF-97416 | PKIS2 | GSK | –4 | 8 | 3 | 8 | –5 | 10 |
| 1085 | SKF-97510 | PKIS2 | GSK | 4 | 0 | 1 | 1 | –7 | 8 |
| 1086 | SKF-97560 | PKIS2 | GSK | 7 | 2 | 4 | 2 | 10 | 24 |
| 1087 | SKF-97620 | PKIS2 | GSK | 1 | 5 | 1 | 0 | 5 | 8 |
| 1088 | SKF-97623 | PKIS2 | GSK | 4 | 1 | –3 | 5 | 10 | 18 |
| 1089 | GI1261590A* | PKIS2 | GSK | 38 | 2 | 27 | 6 | 20 | 2 |
| 1090 | F9005, Fulfenamic acid | AKR1B10 | Sigma | ND | ND | ND | ND | ND | ND |
| 1091 | D6899, Diclofenac sodium salt | AKR1B10 | Sigma | ND | ND | ND | ND | ND | ND |
| 1092 | S8139, Sulindac | AKR1B10 | Sigma | ND | ND | ND | ND | ND | ND |
| 1093 | C2755, Cortisone | AKR1B10 | Sigma | ND | ND | ND | ND | ND | ND |
| 1094 | M4267, Mefenamic acid | AKR1B10 | Sigma | ND | ND | ND | ND | ND | ND |
| 1095 | B6938, BisdemethoxycurcUmin | AKR1B10 | Sigma | ND | ND | ND | ND | ND | ND |
| 1096 | P7265, PGA1 | AKR1B10 | Sigma | ND | ND | ND | ND | ND | ND |
| 1097 | O5504, Oleanolic acid | AKR1B10 | Sigma | ND | ND | ND | ND | ND | ND |

BT, batch reference; mean M%I, mean maximum percentage inhibition; SD, standard deviation; ND, not done.

*Inhibitory hits (*n =* 154) above the selected thresholds (see main text).

^#^Identical compounds of different batches. AKR1B10 inhibitors were tested in an EC_50_ format only, so that no single-point screening data are available for these compounds.
