# Supplementary material for: EGFR inhibitors identified as a potential treatment for chordoma in a focused compound screen
Source: J Pathol. 2016 May 31;239(3):320–34. doi: 10.1002/path.4729 (PMC4922416; doi:10.1002/path.4729)
Supplement: Supplementary file 10 — Table S3. Biochemical selectivity data for EGFR, ERBB2 and ERBB4 of selected EGFR/ERBB inhibitors (n = 14) [file PATH-239-320-s007.docx]

**Suppl. Table 3. Biochemical selectivity data for EGFR, ERBB2, and ERBB4 of selected EGFR/ERBB inhibitors (n=14)**

| **Cpd** | **Parent Compound** | **Chemical Substituent** | **Biochemical Potency** | | |
| --- | --- | --- | --- | --- | --- |
| **ID** |  |  | **EGFR IC_50_ [µM]** | **ERBB2 IC_50_ [µM]** | **ERBB4 IC_50_ [µM]** |
| GW282449A | GW282449 | Quinazoline large | 0.0011 | 0.070 | 0.00051 |
| GW583373A | GW583373 |  | 0.0011 | 0.039 | 0.0082 |
| GW582764A | GW582764 |  | 0.0047 | 0.907 | 0.044 |
| GW616030X | GW616030 |  | 0.0014 | 0.03 | 0.014 |
| GW583340C | GW583340 |  | 0.0097 | 0.082 | 0.0235 |
| GW569716A | GW569716 |  | 0.0256 | 0.079 | 0.00383 |
| GW582764A | GW582764 |  | 0.0407 | 0.442 | 0.0859 |
| GW459135A (erlotinib) | GW459135 | Quinazoline small | 0.00022 | 0.474 | 0.317 |
| GI261607A | GI261607 |  | 0.00035 | 0.062 | 0.105 |
| GW461104A | GW461104 |  | 0.0005 | 0.039 | 0.197 |
| GW459125X (gefitinib) | GW459125 |  | 0.0024 | 0.750 | 0.322 |
| GW680191X | GW680191 |  | 0.00002 | 0.0064 | 0.105 |
| GI230329A | GI230329 |  | 0.00018 | 0.0273 | 0.0558 |
| GSK3413714A (sapitinib) | GSK3413714 |  | 0.00051 | 0.0089 | 0.011 |

nM µM

**Footnote to Suppl. Table 3:** With kind permission from GSK. ERBB3 was not tested as no test kit was available. Quinazoline large: large substituent on aniline in 4-position of the quinazoline ring system. Quinazoline small: small substituent on aniline in 4-position of the quinazoline ring system.
