# Supplementary material for: EGFR inhibitors identified as a potential treatment for chordoma in a focused compound screen
Source: J Pathol. 2016 May 31;239(3):320–34. doi: 10.1002/path.4729 (PMC4922416; doi:10.1002/path.4729)
Supplement: Supplementary file 11 — Table S4. Antibodies and conditions used for western blot analysis [file PATH-239-320-s003.docx]

**Suppl. Table 4. Antibodies and conditions used for western blot analysis**

| **Protein** | **kDA** | **Supplier** | **Cat. Number** | **Species** | **Clonality** | **Dilution** |
| --- | --- | --- | --- | --- | --- | --- |
| **(Clone)** |  |  |  |  |  |  |
| Akt | 60 | Cell Signaling | 9272 | Rabbit | polyclonal | 1:1000 |
| Beta Actin (AC-15) | 42 | Sigma-Aldrich | A5441 | Mouse | monoclonal | 1:5000 |
| Brachyury (A-4) | 49 | Santa Cruz | Sc-374321 | Mouse | monoclonal | 1:500 |
| cMET (D1C2) | 140 | Cell Signaling | 8198 | Rabbit | monoclonal | 1:500 |
| EGFR | 175 | Cell Signaling | 2232 | Rabbit | polyclonal | 1:500 |
| HGF | 83 | Abcam | 83760 | Rabbit | polyclonal | 1:500 |
| p44/42 MAPK (ERK1/2) (3A7) | 44/42 | Cell Signaling | 9107 | Mouse | monoclonal | 1:1000 |
| p-Akt (Ser473) (193H12) | 60 | Cell Signaling | 4058 | Rabbit | monoclonal | 1:1000 |
| p-EGFR (Tyr1068) (D7A5) | 175 | Cell Signalling | 3777 | Rabbit | monoclonal | 1:500 |
| p-EGFR (Tyr1173) | 170 | Merck Millipore | 04-341 | Rabbit | monoclonal | 1:500 |
| p-MET (Tyr1234/1235) (D26) | 145 | Cell Signaling | 3077 | Rabbit | monoclonal | 1:500 |
| p-p44/42 MAPK (ERK1/2) (Thr202/Tyr204) (D13.14.4E) | 44/42 | Cell Signaling | 4370 | Rabbit | monoclonal | 1:500 |
| p-STAT3 (Tyr705) (D3A7) | 79/86 | Cell Signaling | 9145 | Rabbit | monoclonal | 1:500 |
| PTEN (6H2.1) | 54 | Merck Millipore | 04-035 | Mouse | monoclonal | 1:1000 |
| p-YAP (Ser127) (D9W2I) | 65 | Cell Signaling | 13008 | Rabbit | monoclonal | 1:500 |
| STAT3 (124H6) | 79/86 | Cell Signaling | 9139 | Mouse | monoclonal | 1:500 |
| YAP (D8H1X) | 65 | Cell Signaling | 14074 | Rabbit | monoclonal | 1:500 |

**Footnote to Suppl. Table 4:** The antibodies were stored, prepared, and used according to the manufacturers‘ instructions.
