# Supplementary material for: EGFR inhibitors identified as a potential treatment for chordoma in a focused compound screen
Source: J Pathol. 2016 May 31;239(3):320–34. doi: 10.1002/path.4729 (PMC4922416; doi:10.1002/path.4729)
Supplement: Supplementary file 12 — Table S5. Inhibitory hit rates across all compound libraries included in the single‐point focused compound screen [file PATH-239-320-s006.docx]

**Suppl. Table 5. Inhibitory hit rates across all compound libraries included in the single concentration focused compound screen**

| **Library** | **Library Compounds** | **Library Hits** | **Library Hit Rate** |
| --- | --- | --- | --- |
| **ID** | **n** | **n** | **%** |
| Anticancer | 43 | 23 | 53.49 |
| Calbiochem | 160 | 23 | 14.38 |
| GSK PKIS | 365 | 37 | 10.14 |
| GSK PKIS2 | 521 | 71 | 13.63 |
| AKR1B10 | 8 | 0 | 0.00 |
| **All Libraries** | **1097** | **154** | **14.04** |
