# Supplementary material for: EGFR inhibitors identified as a potential treatment for chordoma in a focused compound screen
Source: J Pathol. 2016 May 31;239(3):320–34. doi: 10.1002/path.4729 (PMC4922416; doi:10.1002/path.4729)
Supplement: Supplementary file 14 — Table S7. FISH data of the cell lines included in the study [file PATH-239-320-s005.docx]

**Suppl. Table 7. FISH data of the cell lines included in the study**

| **Cell Line** | **FISH Data** | | |
| --- | --- | --- | --- |
| **ID** | ***EGFR*** | ***ERBB2/HER2*** | ***MET*** |
| **U-CH1** | ***Polysomy***  70% 3-5G + 3-5R | ***Polysomy***  30% 3-5G + 3-5R | ***Polysomy***  70% 3-5G + 3-5R |
| **U-CH2** | ***Polysomy***  70% 3-6G + 3-6R | ***Polysomy***  30% 3-5G + 3-5R | ***Polysomy***  80% 3-7G + 3-7R |
| **U-CH7** | ***Disomy***  2G + 2R | ***Disomy***  2G + 2R | ***Disomy***  2G + 2R |
| **U-CH10** | ***Polysomy***  90% 3-9R + 3-9G | ***Polysomy***  90% 3-13R + 3-13G | ***Polysomy***  90% 3-6R + 3-6G |
| **JHC7** | ***Polysomy***  40% 3-7R + 3-7G | ***Polysomy***  40% 3-7G + 3-7R | ***Polysomy***  40% 3-8G + 3-8R |
| **MUG-Chor1** | ***Polysomy***  80% 3-6G + 3-6R | ***Polysomy***  70% 3-4G + 3-4R | ***Polysomy***  80% 3-5G + 3-5R |
| **UM-Chor1** | ***Polysomy***  90% 3-5R + 3-5G | ***Polysomy***  90% 3-4R + 3-4G | ***Polysomy***  90% 3-6R + 3-4G (R>G) |

**Footnote to Suppl. Table 7:** EGFR/CEP 7: EGFR in red colour (R), centromeric 7 in green colour (G). ERBB2/CEP 17: ERBB2 in green colour (G), centromeric 17 in red colour (R). C-MET/CEN 7: C-MET in green colour (G), centromeric 7 in red colour (R).
